# Lateral septum GABAergic neurons mediate the effects of dexmedetomidine on allodynia and sleep in a male mouse model of neuropathic pain

**DOI:** 10.1038/s41467-026-74217-3

**Published:** 2026-06-19

**Authors:** Haiting Fan, Yingying Su, Jie Deng, Zhengkai Liang, Meiying Chen, Kaibin Wu, Peiwen Tang, Chen Wang, Deyi Kong, Ting Xu, Feixue Liang, Wenjun Xin, Yan Wu, Xia Feng

**Affiliations:** 1 Department of Anesthesiology, The First Affiliated Hospital, Sun Yat-sen University, Guangzhou, China; 2 Guangdong Province Key Laboratory of Brain Function and Disease, Department of Physiology and Pain Research Center, Zhongshan Medical School, Sun Yat-sen University, 74 Zhongshan Road 2, Guangzhou, China; 3 Guangdong-Hong Kong-Macao Greater Bay Area Center for Brain Science and Brain-Inspired Intelligence, School of Biomedical Engineering, Southern Medical University, Guangzhou, China; 4 Guangdong Basic Research Center of Excellence for Integrated Traditional and Western Medicine for Qingzhi Diseases, Southern Medical University, Guangzhou, China; 5 School of Basic Medical Sciences, Guangzhou Medical University, Guangzhou, Guangdong China; 6 Key Laboratory for Molecular Genetic Mechanisms and Intervention Research on High Altitude Disease of Tibet, Autonomous Region, School of Medicine, Xizang Minzu University, Xian Yang, Shanxi China

**Keywords:** Chronic pain, Neural circuits, Sleep

## Abstract

Chronic pain and sleep disorders frequently co-occur and exacerbate each other, yet the neural mechanisms underlying this comorbidity remain poorly understood. Here, we found that hyperactivity of GABAergic neurons in the dorsal division of the lateral septum (LS^GABA^) drives both pain-like behavior and sleep disruption induced by spared nerve injury (SNI) in male mice. We showed that systemic or local application of dexmedetomidine (DEX), with known sedative and analgesic properties, reduces LS^GABA^ hyperactivity via α_2_A adrenergic receptor activation, alleviating pain-like behavior and sleep disruption. LS^GABA^ can project onto the glutamatergic and GABAergic neurons in the lateral preoptic area (LPO), respectively. The LS^GABA^→LPO^Glu^ circuit and the LS^GABA^ → LPO^GABA^ circuit contribute to mechanical allodynia and sleep disruption, respectively. Furthermore, LS α_2_A adrenergic receptor plays an important role in DEX-regulated activity of LPO^GABA^ and LPO^Glu^. Taken together, these findings reveal a shared neural substrate for chronic pain and sleep disorders and establish DEX as a potential treatment for this comorbidity through selective modulation of LS^GABA^-driven circuits.

## Abstract (web summary)

The authors find that dexmedetomidine reduces hyperactivity of lateral septum GABAergic neurons, alleviating pain and sleep disruption induced by spared nerve injury in male mice via the downstream circuits in the lateral preoptic area.

## Introduction

Chronic pain and sleep disorders frequently co-occur and mutually exacerbate one another, substantially complicating treatment. Approximately 50% of chronic pain patients experience sleep disturbances, whereas up to 88% of individuals with sleep disorders report concomitant pain^1,2^. This comorbidity not only impairs memory but also increases the risk of depression, profoundly reducing quality of life^3–5^. Despite its clinical significance, effective strategies for prevention and treatment remain limited, largely due to an incomplete understanding of the underlying neural mechanisms.

The lateral septum (LS) is a key hub for regulating affective behaviors and is traditionally divided into dorsal (LSD), intermediate (LSI), and ventral (LSV) divisions, each characterized by distinct afferent and efferent connections^6^. LS is predominantly composed of GABAergic neurons, with only a sparse population of glutamatergic neurons in its most ventral region^7^. Previous studies have shown that descending LS GABAergic projections to multiple brain regions—including the nucleus accumbens (NAc), vertical and horizontal limbs of the diagonal band (VDB, HDB), dorsomedial hypothalamus (DM), lateral preoptic area (LPO), ventromedial hypothalamus (VMH), lateral hypothalamus (LH), hippocampus (HPC), ventral tegmental area (VTA), and periaqueductal gray (PAG)—play critical roles in modulating sensory and emotional behaviors^8–11^. However, the specific projection patterns of LSD neurons and their functional contributions to chronic pain–sleep disorder comorbidity remain unclear.

Dexmedetomidine (DEX), a selective α_2_-adrenergic receptor agonist, exhibits sedative, sympatholytic, and analgesic properties, and has been effectively applied for postoperative analgesia in procedures such as cesarean delivery and hysterectomy^12–14^. Its sedative effect is primarily mediated via α_2_ adrenoceptors on locus coeruleus norepinephrine neurons, which suppress excitatory norepinephrine release throughout the brain through a Gi-coupled mechanism^15,16^. Additionally, DEX inhibits nociceptive transmission via presynaptic α_2_ receptors in the spinal dorsal horn^17^. Nevertheless, chronic pain–sleep disorder comorbidity involves multiple neurotransmitter systems, numerous brain regions, and extensive neural circuits, and whether DEX alleviates comorbidity by modulating LSD activity and its associated circuits remains unknown.

In the present study, using a spared nerve injury (SNI) model of chronic pain–sleep disruption comorbidity, we demonstrate a pivotal role of LSD GABAergic neurons (LSD^GABA^) in regulating both pain and sleep disruption. We further dissect the functional organization of the LSD^GABA^ circuit in this comorbidity and show that DEX ameliorates symptoms by modulating LSD^GABA^ circuit activity.

## Results

### Dexmedetomidine alleviates the comorbidity symptoms of chronic pain/sleep disruption in mice following SNI

**Fig. 1:**
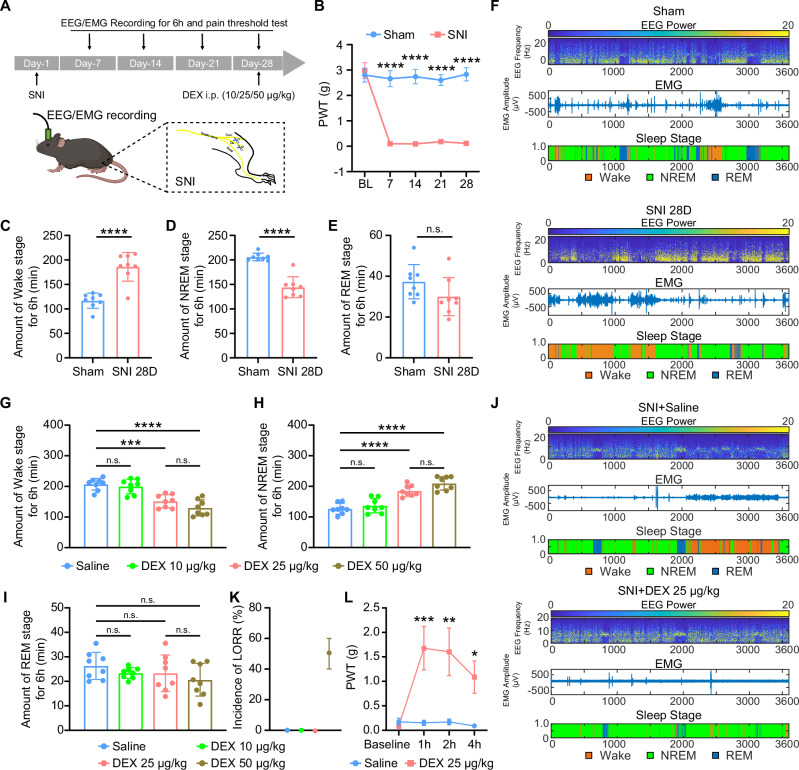
Dexmedetomidine alleviates allodynia and sleep disruption behavior in SNI-induced comorbidity mice. **A** Schematic of SNI (left hind limb) and timeline of EEG/EMG recording and PWT tests (Created in BioRender. Liang, Z. (2026) https://BioRender.com/h8b4ut4). **B** Time course of SNI-induced PWT (Sham, *n* = 6; SNI, *n* = 6; Two-way ANOVA with Sidak’s multiple comparisons, *F*(4,50) = 16.56; baseline, adjusted *p* = 0.9825; days 7–28, adjusted ****p < 0.0001). **C**–**E** Changes in 6-h wake, NREM, and REM duration in Sham and SNI28 groups (Sham, *n* = 8; SNI, *n* = 8; unpaired *t*-test; Wake *t*(14) = 5.901, ****p < 0.0001; NREM *t*(14) = 7.718, ****p < 0.0001; REM *t*(14) = 1.646, *p* = 0.1221). **F** Representative EEG power spectra and EMG traces across sleep–wake states (orange: wake, green: NREM, blue: REM). **G**–**I** Dexmedetomidine effects on 6-h wake, NREM, and REM in SNI mice (*n* = 8 per group; one-way ANOVA with Tukey’s multiple comparisons; Wake *F*(3,28) = 21.56; Saline vs. DEX 10 μg/kg, (n.s.)*p* = 0.9286; Saline vs. DEX 25 μg/kg, ***p = 0.0002; Saline vs. DEX 50 μg/kg, ****p < 0.0001; DEX 25 vs. 50 μg/kg, (n.s.)*p* = 0.2457; NREM *F*(3,28) = 30.02; Saline vs. DEX 10 μg/kg, (n.s.)*p* = 0.7654; Saline vs. DEX 25 μg/kg, ****p < 0.0001; Saline vs. DEX 50 μg/kg, ****p < 0.0001; DEX 25 vs. 50 μg/kg, (n.s.)*p* = 0.0986; REM *F*(3,28) = 1.279; Saline vs. DEX 10 μg/kg, (n.s.)*p* = 0.7389; Saline vs. DEX 25 μg/kg, (n.s.)*p* = 0.7320; Saline vs. DEX 50 μg/kg, (n.s.)*p* = 0.2280; DEX 25 vs. 50 μg/kg, (n.s.)*p* = 0.7918). **J** Representative EEG/EMG traces for saline and DEX groups. **K** LORR percentages during 15-min observation post-injection (*n* = 10/group). **L** Time course of PWT changes after DEX 25 μg/kg or saline in SNI mice (Saline, *n* = 6; DEX, *n* = 6; Two-way ANOVA with Sidak’s multiple comparisons; baseline, adjusted *p* = 0.9989; 1 h, ***adjusted *p* = 0.0008; 2 h, **adjusted *p* = 0.0017; 4 h, *adjusted *p* = 0.0425). Data were presented as mean values ± SD. All tests were two-sided. Source data are provided as a Source Data file. EEG electroencephalogram, EMG electromyogram, DEX dexmedetomidine, NREM nonrapid eye movement sleep, REM rapid eye movement, SNI spared nerve injury, LORR loss of righting reflex, PWT paw withdrawal threshold, i.p. intraperitoneal injection.

Studies have shown that chronic pain is often accompanied by sleep disorders^18^. Using continuous electroencephalogram/electromyogram (EEG/EMG) recordings, we systematically characterized vigilance states of wakefulness, nonrapid eye movement (NREM), and rapid eye movement (REM) sleep in SNI-induced neuropathic pain mice at postoperative days 7, 14, 21, and 28 based on a 6-h recording session (Fig. 1A). SNI mice exhibited sustained mechanical hypersensitivity from day 7 onward (Fig. 1B) and significant sleep disturbances during days 21–28, characterized by increased wakefulness and reduced NREM and REM sleep duration (Fig. 1C–F and Supplementary Fig. 1A–C). These results indicated that SNI induced chronic pain/sleep disruption comorbidity in mice.

To evaluate dexmedetomidine’s therapeutic potential for chronic pain/sleep disruption comorbidity, we administered three doses of dexmedetomidine (10, 25, or 50 μg/kg) via intraperitoneal (i.p.) injection in SNI-induced comorbidity mice, followed by 6 h of EEG/EMG recording to assess sleep–wake architecture. The 25 and 50 μg/kg doses significantly improved sleep architecture by reducing wakefulness and prolonging sleep durations at day 28 post-SNI, whereas 10 μg/kg showed no effect (Fig. 1G–J). In addition, although the 50 μg/kg dose enhanced NREM duration versus 25 μg/kg, both doses similarly normalized wake and REM times (Fig. 1G–J). Importantly, evidence has shown that the loss of righting reflex (LORR) is an important index for the sedative state^19^, we found that the percentage of LORR of 50 μg/kg group was higher than the 25 μg/kg group (Fig. 1K), suggesting 50 μg/kg dose has a potential sedative effect. Accordingly, we selected 25 μg/kg for subsequent experiments. Moreover, 25 μg/kg dexmedetomidine significantly alleviated mechanical allodynia (Fig. 1L) without impairing motor function, including the total distance and center distance in the open field test (Supplementary Fig. 1H–K) and total distance and arm opening distance in the elevated plus maze test (Supplementary Fig. 1L–O). Notably, intraperitoneal administration of neither morphine (5 mg/kg) nor meloxicam (10 mg/kg) improved sleep disturbances despite their analgesic effects (Supplementary Fig. 1D–G). To determine whether dexmedetomidine alters baseline sleep–wake patterns or nociceptive thresholds in the absence of pathology, we examined its effects in sham-operated control mice. Compared with saline-treated sham controls, dexmedetomidine did not significantly change the total duration of wakefulness, NREM sleep, or REM sleep (Supplementary Fig. 2A–D). In addition, paw withdrawal thresholds to mechanical stimulation were not significantly different between dexmedetomidine- and saline-treated sham mice (Supplementary Fig. 2E). These findings demonstrated dexmedetomidine’s unique ability to concurrently address both pain and sleep disruption of SNI-induced comorbidity in mice.

### The comorbidity behavior affects the activity of LSD GABAergic neurons in mice

**Fig. 2:**
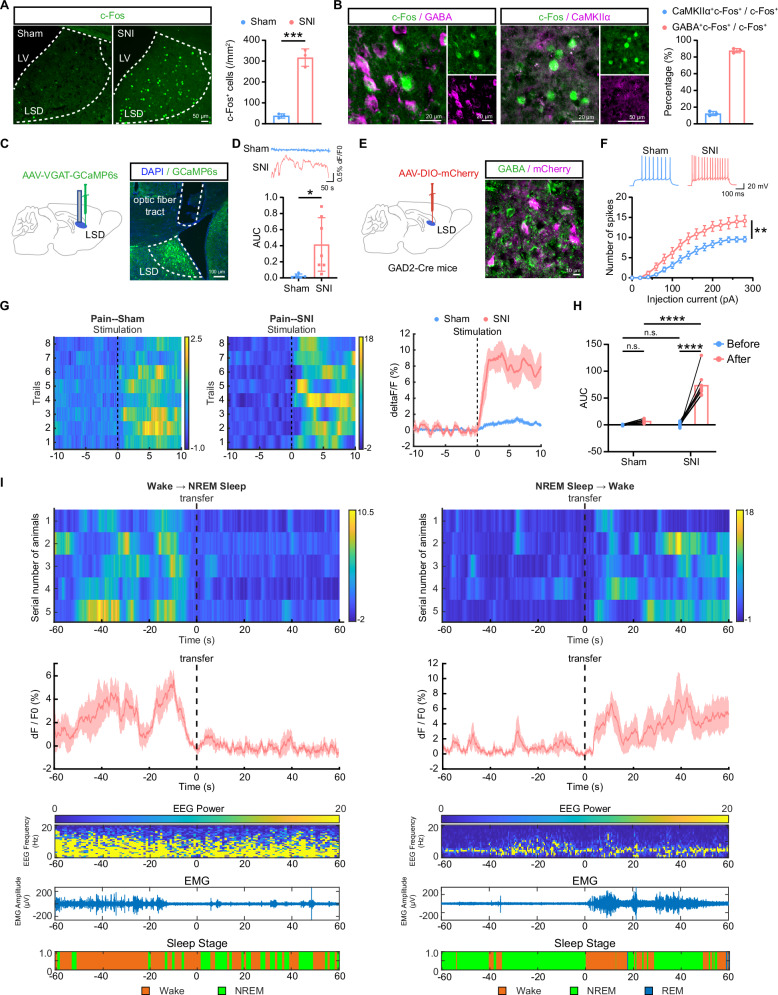
GABAergic neurons in LSD are activated in comorbidity mice with chronic pain and sleep disruption. **A** Left: representative images of c-Fos in LSD regions from different groups; right: quantification of c-Fos-positive cells (scale bars, 50 µm; *n* = 3 mice per group; unpaired *t*-test, *t*(4) = 11.29, ***p = 0.0004). **B** Left: c-Fos-labeled neurons colocalized with glutamate (magenta) or GABAergic (magenta) immunofluorescence in LSD; right: percentage of glutamate+ or GABA^+^ cells among c-Fos-positive (green) neurons (scale bars, 20 µm; *n* = 3 per group). **C** Schematic of virus injection and fiber photometry setup; right: unilateral AAV-VGAT-GCaMP6s targeting in LSD (scale bar, 100 µm). **D** Top: example fiber photometry traces; bottom: AUC quantification from LSD^GABA^ neurons during EEG/EMG-defined wakefulness, NREM and REM excluded (Sham, *n* = 5; SNI, *n* = 7; unpaired *t*-test, *t*(10) = 2.593, *p = 0.0268). **E** Left: Schematic of AAV-DIO-mCherry injection into LSD in GAD2-Cre mice; right: representative immunofluorescence showing colocalization with GABA-positive neurons (scale bar, 10 µm). **F** Top: representative action potential traces; bottom: quantification of firing from LSD^GABA^ neurons (Sham, *n* = 17 cells; SNI, *n* = 16 cells; ordinary two-way ANOVA, *F*(1,31) = 7.716, **p = 0.0092). **G** Left: heatmaps of LSD^GABA^ neurons in Sham and SNI mice during von Frey (2 g) punctate stimulation (Sham, *n* = 8; SNI, *n* = 8); right: average Ca²⁺ transients. **H** AUC quantification of evoked Ca²⁺ responses; two-way repeated-measures ANOVA with Sidak’s multiple comparisons, *F*(1,14) = 45.85, *p* < 0.0001; before stimulation (n.s.) adjusted *p* = 0.9752; after stimulation in SNI, ****adjusted *p* < 0.0001; before-after change SNI, ****adjusted *p* < 0.0001; Sham, (n.s.) adjusted *p* = 0.4614. **I** Heatmaps and average Ca²⁺ transients of LSD^GABA^ neurons during wake-to-NREM and NREM-to-wake transitions in SNI mice (*n* = 5). Color coding: orange indicates wakefulness; green indicates NREM sleep; blue indicates REM sleep. Data were mean ± SD. All tests were two-sided. Source data are provided as a Source Data file.

To elusive the neuronal mechanism underlying dexmedetomidine-relived chronic pain/sleep disruption comorbidity, we explored the expression of c-Fos in various brain regions in mice. Immunofluorescence staining revealed enhanced c-Fos signaling in multiple pain- and sleep-related brain regions 28 days after spared nerve injury (SNI), including the anterior cingulate cortex (ACC), lateral hypothalamus (LH), paraventricular thalamic nucleus (PV), periaqueductal gray (PAG), posterior hypothalamus (PH), and dorsal lateral septum (LSD) (Fig. 2A and Supplementary Fig. 3A, B). Studies showed that LSD is also involved in the process of the sleep–wake circle^19^, we explored the type of c-Fos-expressing cells in comorbidity mice LSD. The double staining results showed that 87.6% c-Fos positive cells were colocalized with GABAergic neurons in LSD (Fig. 2B). Next, we injected the AAV-VGAT-GCaMP6s into the LSD to specific recorded the calcium signal of LSD GABAergic neurons (LSD^GABA^) (Fig. 2C). Compared with the sham group, Ca^2+^ signal in LSD^GABA^ was significantly enhanced in the chronic pain/sleep disruption comorbidity mice (Fig. 2D). Furthermore, whole-cell patch-clamp recordings showed that the number of action potentials evoked by depolarizing currents was obviously increased in the LSD^GABA^ in SNI-induced comorbidity mice relative to the corresponding control group (Fig. 2E, F). The results indicate the increases of GABAergic neuronal activity within the LSD region following nerve injury. Furthermore, in the sham and SNI mice with injecting AAV-VGAT-GCaMP6s into the LSD, the LSD^GABA^ calcium signal increased following pain stimulation using the 2 g von Frey filament (Fig. 2G). AUC quantification of the evoked response further showed a stronger post-stimulus Ca²⁺ increase in SNI mice than in sham controls (Fig. 2H). In addition, during EEG/EMG-defined sleep–wake transitions in SNI mice, LSD^GABA^ Ca²⁺ activity decreased across wake-to-NREM transitions and increased across NREM-to-wake transitions (Fig. 2I and Supplementary Fig. 3F, G). Moreover, similar results were obtained in the sham mice following the change of sleep state (Supplementary Fig. 3C–E). These results indicate that the comorbidity behavior affects the activity of LSD^GABA^ in mice.

### LSD^GABA^ contributed to the comorbidity of chronic pain and sleep disruption

**Fig. 3:**
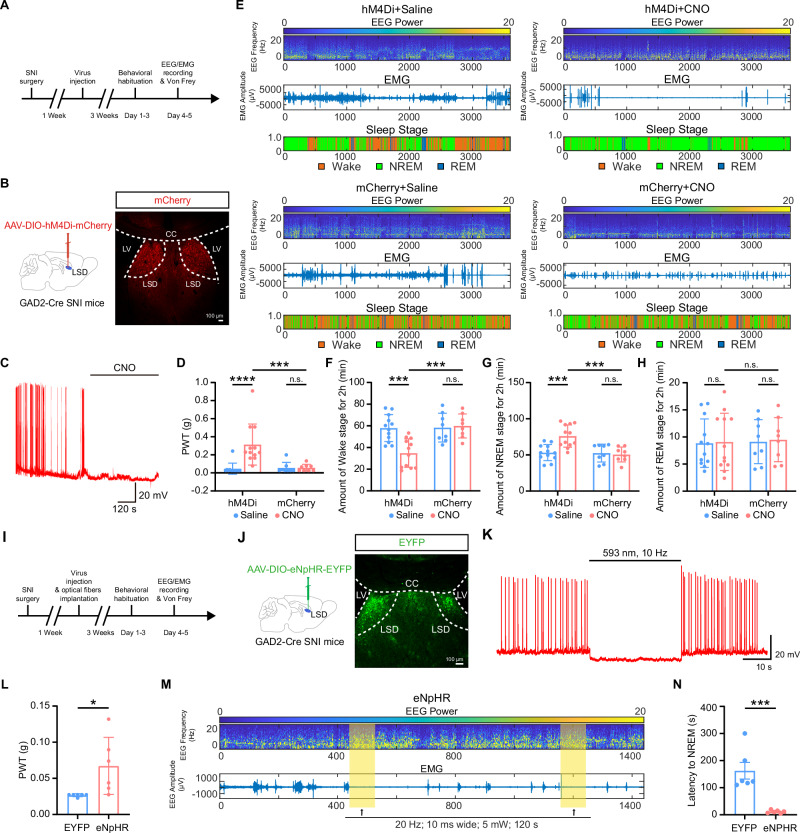
LSD^GABA^ neurons modulate the comorbidity of chronic pain and sleep disruption. **A** Timeline of behavioral assessments after chemogenetic inhibition of LSD^GABA^ neurons. **B** hM4Di injection schematic in LSD of GAD2-Cre mice and representative expression. Scale bars, 100 µm. **C** Whole-cell recordings showing CNO-induced inhibition of LSD^GABA^ neurons after intra-LSD AAV-DIO-hM4Di-mCherry injection. **D**–**H** PWT (**D**) and sleep–wake measures during 2 h (**F**–**H**) after chemogenetic inhibition, including representative EEG/EMG traces and hypnograms (**E**). (hM4Di + Saline, *n* = 12; hM4Di + CNO, *n* = 12; mCherry + Saline, *n* = 8; mCherry+CNO, *n* = 8, Two-way ANOVA with Sidak’s test: PWT, interaction *F*(1,36) = 9.433, *p* = 0.0040; hM4Di Saline vs CNO, ****p < 0.0001; mCherry Saline vs CNO, (n.s.) *p* = 0.9998; hM4Di vs mCherry under Saline, *p* = 0.9852; under CNO, ***p = 0.0003. Wake: interaction *F*(1,36) = 9.256, *p* = 0.0044; hM4Di Saline vs CNO, ***p = 0.0001; mCherry Saline vs CNO, (n.s.) *p* = 0.9609; hM4Di vs mCherry under Saline, *p* = 0.9974; under CNO, ***p = 0.0002. NREM: interaction *F*(1,36) = 9.231, *p* = 0.0044; hM4Di Saline vs CNO, ***p = 0.0002; mCherry Saline vs CNO, (n.s.) *p* = 0.9421; hM4Di vs mCherry under Saline, *p* = 0.9920; under CNO, ***p = 0.0002. REM: interaction *F*(1,36) = 0.0017, *p* = 0.9670; hM4Di Saline vs CNO, (n.s.) *p* = 0.9893; mCherry Saline vs CNO, (n.s.) *p* = 0.9840; hM4Di vs mCherry under Saline, *p* = 0.9889; under CNO, (n.s.) *p* = 0.9770). **I** Timeline of optogenetic inhibition of LSD^GABA^ neurons. **J** Schematic and representative eNpHR expression in LSD. Scale bars, 100 µm. **K** Whole-cell recordings showing yellow light-induced inhibition of eNpHR neurons. **L** PWT after yellow light stimulation (EYFP, *n* = 6; eNpHR, *n* = 6; unpaired t-test, *t*(10) = 2.506, *p = 0.0311). **M** Representative EEG/EMG traces during optogenetic inhibition (593 nm, 20 Hz, 10 ms, 5 mW, 120 s, 10 min intervals). **N** Latency to NREM after optical silencing during wakefulness (EYFP, *n* = 6; eNpHR, *n* = 6; unpaired *t*-test, *t*(10) = 4.970, ***p = 0.0006). Data were mean ± SD. All tests two-sided. Source data are provided as a Source Data file.

To establish the role of LSD^GABA^ in the comorbidity, we first detected the change in comorbidity behavior of chronic pain and sleep disruption by inhibiting the excitability of LSD^GABA^ neurons in SNI mice. In GAD2-Cre mice receiving bilateral stereotaxic injections of AAV-DIO-hM4Di-mCherry into the LSD, the obvious mCherry-positive signal in the local area was observed, indicating the accuracy and effectiveness of the virus injection (Fig. 3A, B). Electrophysiological records showed that incubation of clozapine-N-oxide (CNO, 5 μM) in slices significantly reduced the increased number of LSD^GABA^ action potentials induced by SNI on day 28, further confirming the specific regulatory effect of virus (Fig. 3C). The behavioral results showed that the inhibition of LSD^GABA^ with CNO (1 mg/kg, i.p.) significant attenuated the mechanical allodynia on day 28 post-SNI (Fig. 3D). Importantly, the results of EEG and EMG showed that CNO treatment (1 mg/kg, i.p.) decreased the wakefulness and increased the period of NREM in mice with 28-day of SNI relative to the saline group (Fig. 3E–H). To control for potential off-target actions of CNO, we included mCherry-only control cohorts (mCherry+saline and mCherry+CNO), and confirmed that CNO alone did not alter Wake/NREM/REM or PWT, supporting the specificity of the hM4Di-mediated effects (Fig. 3D, F–H). Furthermore, in GAD2-Cre mice with injection of AAV-DIO-eNpHR-eYFP, the optogenetic results showed that yellow light stimulation (593 nm, 5 mW) inhibited the activity of LSD^GABA^ and attenuated the mechanical allodynia following SNI (Fig. 3I–L). Moreover, inhibition of the LSD^GABA^ activity by using yellow light stimulation shortened the latency of transiting from the awake state to NREM (Fig. 3M, N).

Furthermore, optogenetic activation showed that blue light stimulation of ChR2-expressing LSD^GABA^ neurons increased neuronal firing (Supplementary Fig. 4A, B) and induced mechanical allodynia (Supplementary Fig. 4C), while also promoting NREM-to-wake transitions in naïve mice (Supplementary Fig. 4D, E). Consistently, optogenetic stimulation increased wakefulness and concomitantly reduced NREM and REM sleep during light delivery (Supplementary Fig. 4F–H).

Moreover, we also observed comorbid phenotypes by increasing the excitability of LSD^GABA^ neurons in naïve mice via chemogenetic activation. In GAD2-Cre naïve mice with intra-LSD injection of AAV-DIO-hM3Dq-mCherry (Supplementary Fig. 4I, J), CNO treatment increased LSD^GABA^ activity (Supplementary Fig. 4K) and induced mechanical allodynia (Supplementary Fig. 4L). EEG/EMG recordings further showed that chemogenetic activation of LSD^GABA^ increased wakefulness and reduced sleep time (Supplementary Fig. 4M–O). Likewise, we included mCherry-only control cohorts (mCherry + saline and mCherry + CNO) and confirmed that CNO alone did not affect Wake/NREM/REM or PWT, supporting DREADD-dependent specificity (Supplementary Fig. 4L–P). Together, these results provide evidence that elevated LSD^GABA^ excitability is sufficient to drive the comorbidity of chronic pain and sleep disruption.

### Dexmedetomidine inhibits LSD^GABA^ neuronal hyperactivity in SNI mice

**Fig. 4:**
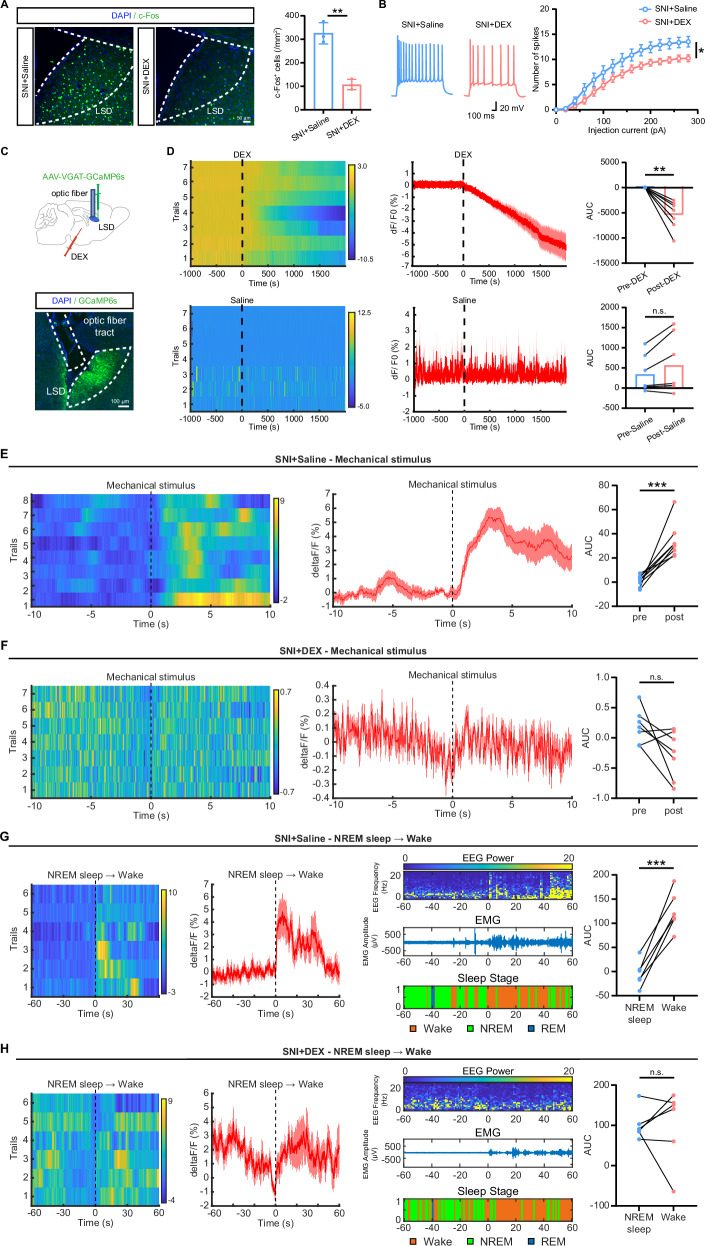
Dexmedetomidine inhibits the hyperactivity of LSD^GABA^ neurons in SNI mice. **A** Left: representative c-Fos immunofluorescence images in SNI mice following saline or DEX administration. Right: quantification of c-Fos^+^ cells in the LSD (SNI + Saline, *n* = 3; SNI + DEX, *n* = 3; unpaired *t*-test, *t*(4) = 7.509, **p = 0.0017). Scale bar, 50 µm. **B** Left: representative traces of action potential firing recorded from LSD^GABA^ neurons in SNI mice treated with saline or DEX. Right: summarized data of action potential firing in LSD^GABA^ neurons (Saline, *n* = 17 cells; DEX, *n* = 17 cells; Two-way RM ANOVA, *F*(1,32) = 4.779, *p = 0.0362). **C** Schematic diagram (upper) and representative image (lower) showing GCaMP6s virus injection into the LSD and DEX administration. Scale bar, 100 µm. **D** Heatmaps, averaged Ca²⁺ transients, and AUC analysis of LSD^GABA^ neurons before and after DEX or saline administration (Pre-DEX vs. Post-DEX, *n* = 7, paired *t*-test, *t*(6) = 5.023, ** *p* = 0.0024; pre-saline vs. post-saline, *n* = 7, paired *t*- test, *t*(6) = 2.152, *p* = 0.0749). **E** Heatmap, averaged Ca²⁺ responses, and AUC analysis of LSD^GABA^ neurons before and after mechanical stimulation in the SNI+Saline group (*n* = 8, paired *t*-test, *t*(7) = 5.472, ***p = 0.0009). **F** Heatmap, averaged Ca²⁺ responses, and AUC analysis of LSD^GABA^ neurons before and after mechanical stimulation in the SNI + DEX group (*n* = 7, paired *t*-test, *t*(6) = 2.272, *p* = 0.0635). **G** Heatmap, averaged Ca²⁺ responses, representative sleep-stage trace, and AUC analysis of LSD^GABA^ neurons during the transition from NREM sleep to wakefulness in the SNI + saline group (*n* = 6, paired *t*-test, *t*(5) = 9.029, ***p = 0.0003). **H** Heatmap, averaged Ca²⁺ responses, representative sleep-stage trace, and AUC analysis of LSD^GABA^ neurons during the transition from NREM sleep to wakefulness in the SNI + DEX group (*n* = 6, paired *t*- test, *t*(5) = 0.07036, *p* = 0.9466). Data were mean ± SD. All tests were two-sided. Source data are provided as a Source Data file.

Next, we examined whether dexmedetomidine affects the abnormal activity of LSD^GABA^ neurons in SNI mice. First, intra-LSD injection of dexmedetomidine reduced the SNI-induced upregulation of c-Fos in the LSD (Fig. 4A). Electrophysiological recordings further showed that dexmedetomidine decreased the enhanced action potential firing of LSD^GABA^ neurons in SNI mice (Fig. 4B). Fiber photometry analysis showed that dexmedetomidine also reduced the elevated Ca²⁺ activity of LSD^GABA^ neurons in SNI mice (Fig. 4C, D). To further characterize the effects of dexmedetomidine on the activity dynamics of LSD^GABA^ neurons, we analyzed Ca²⁺ signals during mechanical stimulation and the transition from NREM sleep to wakefulness. During mechanical stimulation, saline-treated SNI mice exhibited an increase in Ca²⁺ signals, and the AUC analysis also showed a clear difference before and after stimulation (Fig. 4E). In contrast, no increase in Ca²⁺ signals after mechanical stimulation was observed in SNI mice treated with dexmedetomidine (Fig. 4F). During the transition from NREM sleep to wakefulness, saline-treated SNI mice showed an increase in Ca²⁺ signals, and the AUC analysis revealed a significant difference between the two states (Fig. 4G). By contrast, no obvious change in Ca²⁺ signals between NREM sleep and wakefulness was observed in dexmedetomidine-treated SNI mice (Fig. 4H). Together, these findings indicate that dexmedetomidine inhibits the hyperactivity of LSD^GABA^ neurons in SNI mice.

### Dexmedetomidine relieves chronic pain/ sleep disruption comorbidity via α_2_A receptor-mediated inhibition of LSD^GABA^ hyperactivity

**Fig. 5:**
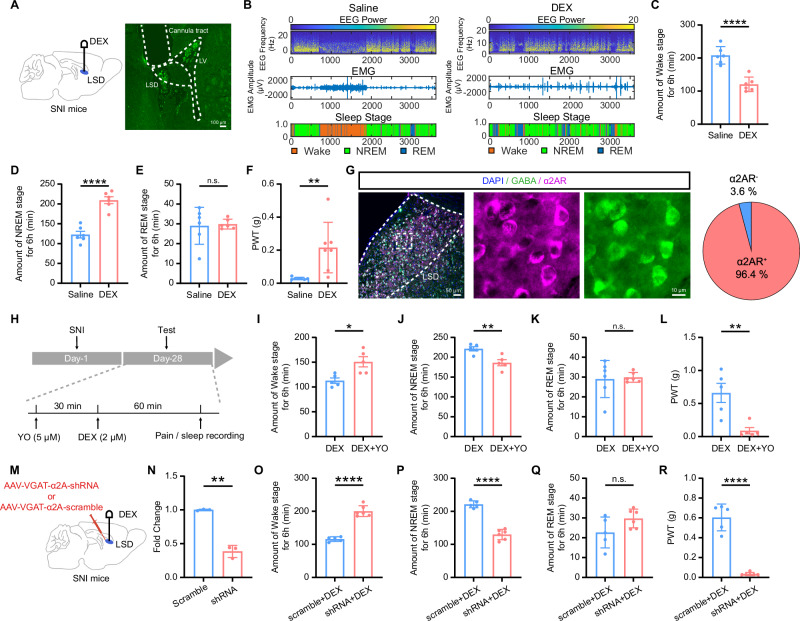
Dexmedetomidine ameliorates the comorbidity of chronic pain and sleep disruption through the α_2_A adrenergic receptor. **A** Schematic and representative image of catheter-based DEX administration into LSD. Scale bars, 100 µm. **B** Relative EEG power spectra and EMG traces across sleep–wake states after intra-LSD-saline or DEX microinjection (2 μM). **C**–**E** Wake, NREM, and REM duration after intra-LSD-saline or DEX microinjection (Saline, *n* = 6; DEX, *n* = 6; unpaired *t*- test: Wake, *t*(10) = 6.252, ****p < 0.0001; NREM, *t*(10) = 7.223, ****p < 0.0001; REM, *t*(10) = 0.2196, *p* = 0.8306). **F** PWT after intra-LSD-saline or DEX microinjection (Saline, *n* = 7; DEX, *n* = 7; unpaired *t*- test, *t*(12) = 3.245, **p = 0.0070). **G** α2A receptor (magenta) expression in LSD^GABA^ neurons (green). Left: representative immunofluorescence images (scale bars, 50 and 10 µm); right: pie chart of colocalization analysis (*n* = 5 mice). **H** Timeline of yohimbine injection. **I**–**K** Wake, NREM, and REM duration after DEX or DEX + YO administration (DEX, *n* = 5; DEX + YO, *n* = 5; unpaired *t*-test: Wake, *t*(8) = 3.268, *p = 0.0114; NREM, *t*(8) = 3.734, **p = 0.0058; REM, *t*(8) = 0.4963, *p* = 0.6330). **L** PWT after DEX or DEX + YO administration (DEX, *n* = 5; DEX + YO, *n* = 5; unpaired *t*-test, *t*(8) = 3.753, **p = 0.0056). **M** Schematic of catheter-based AAV-VGAT-α2A-shRNA or AAV-VGAT-α2A-scramble administration into LSD. **N** qPCR confirmed reduced Adra2a mRNA in the α2A-shRNA group compared with scramble controls (*n* = 3; unpaired *t*-test, *t*(2.056) = 11.84, **p = 0.0064). **O**–**Q** Wake, NREM, and REM duration after DEX administration in scramble and α2A-shRNA groups (scramble, *n* = 5; shRNA, *n* = 6; unpaired *t*-test: Wake, *t*(9) = 10.64, ****p < 0.0001; NREM, *t*(9) = 11.13, ****p < 0.0001; REM, *t*(9) = 1.852, *p* = 0.0971). **R** PWT after DEX administration in scramble and α2A-shRNA groups (scramble, *n* = 5; shRNA, *n* = 6; unpaired *t*- test, *t*(9) = 10.39, ****p < 0.0001). Data were mean ± SD. All tests were two-sided. Source data are provided as a Source Data file.

To determine whether the LSD mediates the beneficial effects of dexmedetomidine on chronic pain/ sleep disruption comorbidity. Importantly, we first implanted cannulas into the LSD for local drug administration (Fig. 5A). Intra-LSD injection of dexmedetomidine alleviated mechanical allodynia, increased NREM sleep time, and decreased wake time in SNI mice (Fig. 5B–F), indicating that the LSD is a key site mediating the analgesic and sleep-promoting effects of dexmedetomidine. It is well known that dexmedetomidine is a highly selective α_2_A adrenergic receptor agonist, and we observed robust α_2_A receptor expression in the LSD, with 96.4% of LSD GABAergic neurons colocalizing with α_2_A receptors (Fig. 5G). We further confirmed through pharmacology and specific knockdown whether dexmedetomidine alleviates comorbidity behavior through α_2_A adrenergic receptor. The results showed that pre-injection of α_2_ adrenergic receptor antagonist yohimbine significantly inhibited the improving effect of dexmedetomidine on mechanical allodynia and sleep disruption (Fig. 5H–L). Moreover, special knockdown of α_2_A adrenergic receptor by intra-LSD injection AAV-VGAT-α_2_A-shRNA prevented the alleviating the effect of dexmedetomidine on comorbidities (Fig. 5M–R). These results suggested that dexmedetomidine relieves the comorbidity of chronic pain/ sleep disruption by decreasing the hyperactivity of LSD^GABA^ in mice.

### The LSD^GABA^→LPO^Glu^ circuit contributed to the mechanical allodynia in comorbidity mice

**Fig. 6:**
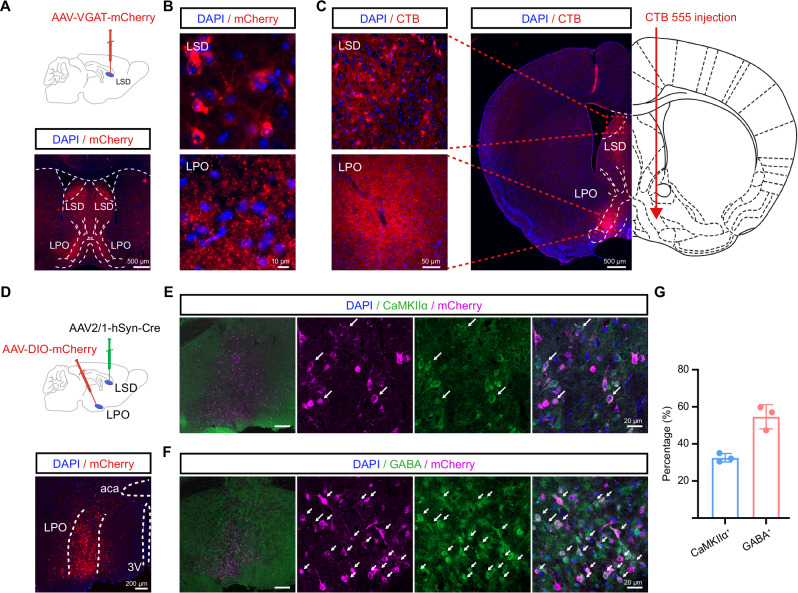
LSD^GABA^ neurons projecting to LPO GABAergic and glutamatergic neurons. **A** Schematic (upper) and representative imaging (lower) of AAV-VGAT-mCherry virus expression in LSD. Scale bars: 500 µm. **B** Representative imaging of mCherry^+^ expression in LSD and terminal projections in LPO (images are representative of three independent experiments with similar results), scale bar: 10 μm. **C** Schematic and representative images of CTB-555 retrograde tracer injection in LPO (scale bar: 500 μm). Top: retrogradely labeled CTB^+^ soma in LSD (scale bar: 50 μm). Bottom: CTB^+^ terminals in LPO (scale bar: 50 μm). The experiment was independently repeated three times with comparable results. **D** Schematic and representative images of AAV2/1-hSyn-Cre virus expression in LSD and AAV-DIO-mCherry in LPO. Scale bars, 200 µm. **E**, **F** Showing mCherry-labeled neurons (magenta) colocalized with glutamatergic (**E**, top, green) or GABAergic (**F**, bottom, green) immunofluorescence within the LPO. Scale bars: 200 µm (left), 20 µm (right). White arrows indicate colocalization. Representative images are from three independent repeats. **G** Percentage of mCherry^+^ neurons colocalized with glutamatergic or GABAergic markers (*n* = 3 mice). Data were mean ± SD. Source data are provided as a Source Data file.

To explore the neuronal mechanism in SNI-induced chronic pain/sleep disruption comorbidity in mice, we first injected the anterograde virus (AAV2/9-VGAT-mCherry) into the LSD. Three weeks later, the fluorescence expression of mCherry^+^ fibers were observed in many regions, including the lateral preoptic area (LPO), vertical diagonal band (VDB), horizontal diagonal band (HDB), lateral hypothalamus (LH), prefrontal cortex (PFC), hippocampus (HP), and lateral interstitial nucleus of the posterior limb of the anterior commissure (IPACL) (Fig. 6A, B and Supplementary Fig. 5A, B). As an evolutionarily conserved nucleus within the hypothalamus, the LPO modulates multiple behavioral outputs and core temperature homeostasis in physiological conditions^20^. CTB retrograde tracing in the LPO further confirmed the existence of direct anatomical projections from the LSD to LPO (Fig. 6C). To clarify the type of LPO neurons which received the LSD projection, we injected the anterograde trans-synaptic virus (AAV2/1-hSyn-Cre) into the LSD and the AAV-DIO-mCherry into the LPO (Fig. 6D). The immunofluorescence staining showed that 58.06% mCherry-positive cells were expressed in the GABAergic neurons and 32.53% of that in glutamatergic neurons in LPO (Fig. 6E–G), suggesting that the LSD^GABA^ neurons fibers innervate the LPO^GABA^ neurons and LPO^Glu^ neurons.

**Fig. 7:**
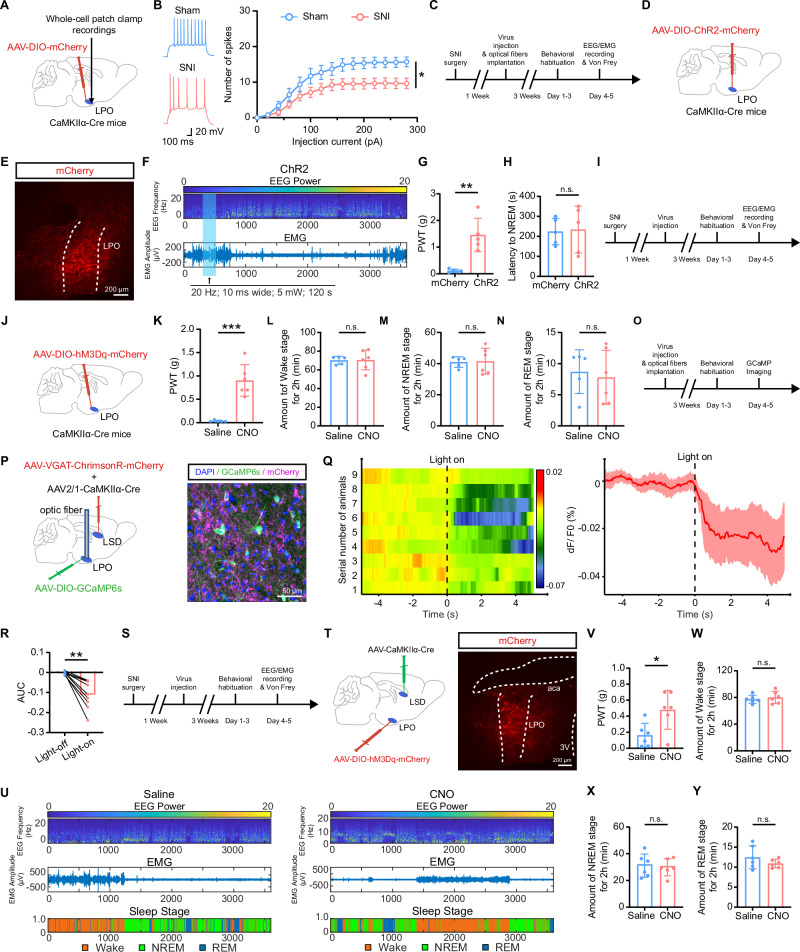
The LSD^GABA^→LPO^Glu^ circuit modulates chronic pain in comorbidity mice without affecting sleep. **A** Schematic of AAV-DIO-mCherry injection into LPO in CaMKIIα-Cre mice. **B** Left: representative firing trace; right: quantification of action potential firing from LPOGlu neurons (Sham, *n* = 14 cells; SNI, *n* = 11 cells; ordinary two-way ANOVA, *F*(1,23) = 5.587, *p = 0.0269). **C** Timeline for optogenetic activation of LPO CaMKIIα neurons. **D**, **E** Schematic and representative immunofluorescence of AAV-DIO-ChR2-mCherry expression in LPO. Scale bar, 200 μm. **F** Relative EEG power spectra and EMG traces across sleep–wake states after blue light stimulation. **G**, **H** PWT and latency to NREM after blue light stimulation (*n* = 5; unpaired *t*-test: PWT, *t*(8) = 4.774, **p = 0.0014; NREM latency, *t*(8) = 0.1294, *p* = 0.9003). **I**, **J** Timeline and schematic of chemogenetic activation of LPO CaMKIIα neurons by AAV-DIO-hM3Dq-mCherry injection into LPO. **K**–**N** PWT and sleep–wake durations after saline or CNO injection (Saline, *n* = 5; CNO, *n* = 6; unpaired *t*-test: PWT, *t*(9) = 5.691, ***p = 0.0003; Wake, *t*(9) = 0.06622, *p* = 0.9487; NREM, *t*(9) = 0.1398, *p* = 0.8919; REM, *t*(9) = 0.3709, *p* = 0.7193). **O** Timeline of calcium signal recording. **P** Left: schematic of viral injections in LSD and LPO with fiber implantation; right: representative fluorescence images showing LSD^GABA^ terminals labeled by mCherry (magenta) and LPO neurons labeled by GCaMP6s (green). Scale bars, 50 μm. **Q**, **R** Heatmap and line graph showing reduced calcium activity after red light stimulation of LSD^GABA^–LPO^Glu^ connections, with AUC quantification (*n* = 9; paired *t*-test, *t*(8) = 4.859, **p = 0.0013). **S** Behavioral timeline. **T** Schematic and representative image of AAV2/1-CaMKIIα-Cre injection in LSD and AAV-DIO-hM3Dq-mCherry expression in LPO. Scale bar, 200 μm. **U** Relative EEG power spectra and EMG traces. **V**–**Y** PWT and sleep–wake durations after CNO or saline injection during activation of LSD^GABA^→LPO^Glu^ projections (*n* = 6; unpaired *t*-test: PWT, *t*(10) = 2.734, *p = 0.0210; Wake, *t*(10) = 0.6026, *p* = 0.5602; NREM, *t*(10) = 0.2789, *p* = 0.7860; REM, *t*(10) = 1.253, *p* = 0.2387). Data were mean ± SD. All tests were two-sided. Source data are provided as a Source Data file.

Next, we first explored the role of LPO^Glu^ in the comorbidity. In CaMKIIα-Cre mice with injection of AAV-DIO-mCherry into LPO, the electrophysiological results showed that the number of evoked action potential of CaMKIIα-positive neurons significantly decreased in comorbidity mice (Fig. 7A, B). Next, we manipulated LPO^Glu^ activity using optogenetic activation approach, in which the obvious mCherry-positive signal in LPO indicated the effectiveness of virus transfection (Fig. 7C–E). The results showed that blue light stimulation (473 nm, 5 mW) of LPO^Glu^ neurons reversed SNI-induced mechanical allodynia, while it did not affect the sleep disruption in comorbidity mice (Fig. 7F–H). The chemogenetic activation of LPO^Glu^ neurons also showed that CNO administration attenuated the mechanical allodynia, but did not affect the sleep disruption in CaMKIIα-Cre mice with injection of AAV-DIO-hM3Dq-mCherry on day 28 following SNI (Fig. 7I–N). Moreover, yellow light stimulation (593 nm, 5 mW) induced the mechanical allodynia, but did not change the sleep behavior in CaMKIIα-Cre naïve mice with injection of AAV-DIO-eNpHR-mCherry into LPO (Supplementary Fig. 5C–E). In addition, in CaMKIIα-Cre naïve mice with injection of intra-LPO AAV-DIO-hM4Di-mCherry, CNO treatment significantly reduced the mechanical withdrawal threshold, but not affect the sleep status in naïve mice (Supplementary Fig. 5F–J). The above results indicated that the decreased LPO^Glu^ neurons activity contributed to the painful behavior in comorbidity mice.

To clarify the role of LSD^GABA^→LPO^Glu^ circuit in the comorbidity, we injected AAV-VGAT-ChrimsonR-mCherry and AAV2/1-CaMKIIα-Cre into the LSD and AAV-DIO-GCaMP6s into LPO (Fig. 7O, P). The results of optical fiber photometer recordings showed that activation of LSD^GABA^ terminals decreased the Ca^2+^ signals of LPO^Glu^ neurons (Fig. 7Q, R), suggesting the functional connection of LSD^GABA^→LPO^Glu^ circuit. Besides, in LPO^Glu^ neurons receiving inputs from LSD^GABA^ projections, SNI mice showed a reduced paired-pulse ratio (PPR) of optogenetically evoked IPSCs compared with sham controls, consistent with altered presynaptic release properties (Supplementary Fig. 7A, B). In addition, the IPSC input-output (I–O) curve was shifted downward in the SNI group, indicating weakened synaptic strength of LSD^GABA^ inputs onto these LPO^Glu^ neurons (Supplementary Fig. 7C). Next, we microinjected AAV2/1-CaMKIIα-Cre into LSD and delivered Cre-dependent chemogenetics and optogenetics viruses into LPO to selectively manipulate LPO^Glu^ neurons receiving LSD^GABA^ projection (Fig. 7S, T and Supplementary Fig. 5K). Activation of LSD^GABA^-projected LPO^Glu^ neurons with intraperitoneal injection of CNO or blue light stimulation (473 nm) alleviated mechanical allodynia in comorbidity mice, but did not affect the sleep status (Fig. 7U–Y and Supplementary Fig. 5L–N). Consistently, inhibition of LSD-projected LPO^Glu^ neurons by using the means of optogenetics (yellow lights) induced the mechanical allodynia in naïve mice, but did not affect the sleep status (Supplementary Fig. 5O–R). These results showed that LSD^GABA^→LPO^Glu^ circuit participated in the painful behavior in comorbidity mice.

### The LSD^GABA^ → LPO^GABA^ circuit contributed to the sleep disruption in comorbidity mice

**Fig. 8:**
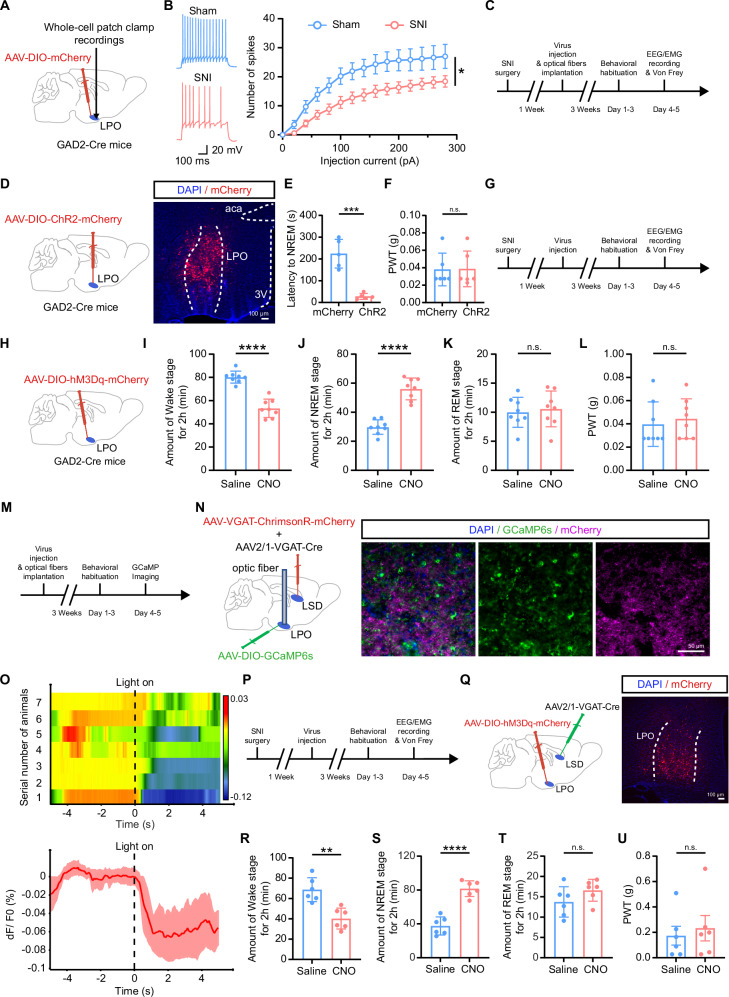
The LSD^GABA^ → LPO^GABA^ circuit modulates sleep disruption in comorbidity mice without altering pain. **A** Schematic of AAV-DIO-mCherry injection into LPO in GAD2-Cre mice. **B** Left: representative firing trace; right: quantification of action potential firing from LPO^GABA^ neurons (Sham, *n* = 13 cells; SNI, *n* = 12 cells; ordinary two-way ANOVA, *F*(1,23) = 7.579, *p = 0.0113). **C** Timeline for optogenetic activation of LPO^GABA^ neurons. **D** Schematic and representative image of AAV-DIO-ChR2-mCherry expression in LPO of GAD2-Cre mice. Scale bars, 100 µm. **E**, **F** Effects of blue light activation of LPO^GABA^ neurons on NREM latency and PWT (unpaired *t*-test for NREM latency, *n* = 5, *t*(8) = 6.527, ***p = 0.0002; Mann–Whitney test for PWT, *n* = 6, *U* = 14, *p* = 0.5909). **G**, **H** Timeline and schematic of chemogenetic activation of LPO^GABA^ neurons by AAV-DIO-hM3Dq-mCherry injection into LPO. **I**–**L** Wake, NREM, REM durations and PWT after saline or CNO injection (Saline, *n* = 8; CNO, *n* = 8; unpaired *t*-test: Wake, *t*(14) = 8.029, ****p < 0.0001; NREM, *t*(14) = 8.250, ****p < 0.0001; REM, *t*(14) = 0.4055, *p* = 0.6913; PWT, *t*(14) = 0.5170, *p* = 0.6133). **M** Timeline of calcium signal recording. **N** Left: schematic of viral injections in LSD and LPO with fiber implantation; right: representative fluorescence images showing LSD^GABA^ terminals labeled by mCherry (magenta) and LPO neurons labeled by GCaMP6s (green). Scale bars, 50 μm. **O** Heatmap and average Ca²⁺ transients showing reduced calcium activity after red light stimulation of LSD^GABA^–LPO^GABA^ connections (*n* = 7 mice). **P** Behavioral timeline. **Q** Left: schematic of AAV2/1-VGAT-Cre injection in LSD and AAV-DIO-hM3Dq-mCherry injection in LPO; right: representative image. Scale bar, 100 μm. **R**–**U** Wake, NREM, REM durations, and PWT after CNO or saline injection during activation of the LSD^GABA^ → LPO^GABA^ circuit (*n* = 6; unpaired *t*-test: Wake, *t*(10) = 4.449, **p = 0.0012; NREM, *t*(10) = 7.625, ****p < 0.0001; REM, *t*(10) = 1.516, *p* = 0.1605; PWT, *t*(10) = 0.4746, *p* = 0.6453). Data were mean ± SD. All tests were two-sided. Source data are provided as a Source Data file.

Next, we examined the role of LPO^GABA^ in the comorbidity behavior. The results showed that the number of action potential of LPO^GABA^ (mCherry-positive cells) was lower in comorbidity mice than that in the sham mice (Fig. 8A, B). Moreover, we injected the AAV-DIO-ChR2-mCherry into LPO of GAD2-Cre mice, the results of optogenetic approach showed that activation of LPO^GABA^ prompt the transmit from the wakefulness to the sleep status, but not affect the mechanical allodynia (Fig. 8C–F). Similarly, we injected the Cre-dependent chemogenetic virus (AAV-DIO-hM3Dq-mCherry) into LPO of GAD2-Cre mice (Fig. 8G, H). CNO treatment significantly increased the NREM sleep time and decreased the wake time, but did not affect the mechanical withdrawal threshold in comorbidity mice (Fig. 8I–L). Furthermore, inhibition of LPO^GABA^ by using the means of chemogenetics and optogenetics (yellow lights) decreased the sleep time, but not affect the withdrawal threshold, in naïve mice (Supplementary Fig. 6A–G). The results suggested that the decreased excitability of LPO^GABA^ contributed to the sleep disruption in the comorbidity mice.

To further identified the role of LSD^GABA^ → LPO^GABA^ circuit in the comorbidity, we injected AAV-VGAT-ChrimsonR-mCherry and AAV2/1-VGAT-Cre into the LSD and AAV-DiO-GCaMP6s into LPO (Fig. 8M, N). The results of optical fiber photometer recordings showed that activation of LSD^GABA^ terminals decreased the Ca^2+^ signals of LPO^GABA^ neurons (Fig. 8O). These data suggest the functional connection of LSD^GABA^ → LPO^GABA^ circuit. In LPO^GABA^ neurons receiving inputs from LSD^GABA^ projections, SNI mice exhibited a lower PPR of optogenetically evoked IPSCs relative to sham controls (Supplementary Fig. 7D, E), suggesting changes in presynaptic release dynamics. Moreover, the IPSC input-output (I–O) relationship was reduced in the SNI group, consistent with an overall attenuation of synaptic transmission strength from LSD^GABA^ terminals onto these LPO^GABA^ neurons (Supplementary Fig. 7F). Behavioral experiments demonstrated that chemogenetic activation (via CNO) of the LSD^GABA^ → LPO^GABA^ circuit by injecting AAV2/1-VGAT-Cre into LSD and AAV-DIO-hM3Dq-mCherry into LPO, significantly improved sleep disturbances without altering mechanical withdrawal thresholds in comorbid mice (Fig. 8P–U). Similarly, optogenetics methods also confirmed that the activation of LSD^GABA^ → LPO^GABA^ circuit also promotes the transition from wakefulness to sleep (Supplementary Fig. 6K–M). Moreover, inhibition of LSD^GABA^ → LPO^GABA^ activity by the optogenetic methods lead to the sleep disruption, but not affect the withdrawal threshold, in naïve mice following injected AAV2/1-VGAT-Cre into LSD and AAV-DIO-eNpHR-mCherry into LPO (Supplementary Fig. 6H–J). These results suggested that LSD^GABA^ → LPO^GABA^ circuit participated in the sleep disruption in comorbidity mice.

### Dexmedetomidine alters the activity of LPO^GABA^ and LPO^Glu^ by acting on the LSD α_2_A adrenergic receptor

**Fig. 9:**
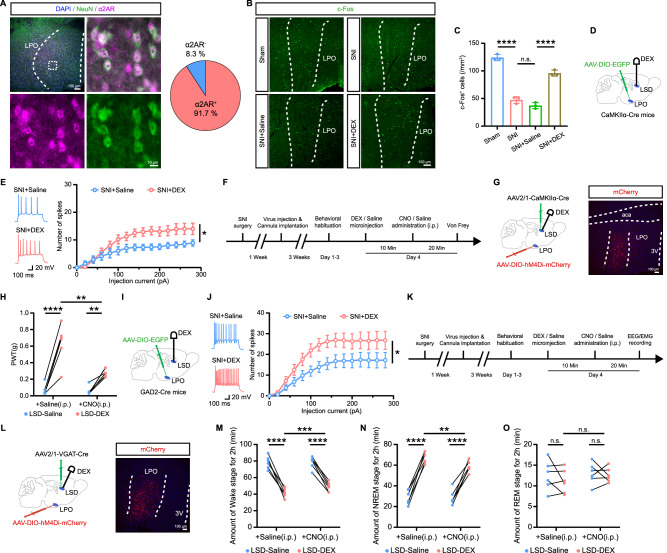
Dexmedetomidine alters the activity of LPO^GABA^ and LPO^Glu^ by acting on the LSD α_2_A adrenergic receptor. **A** α_2_A receptor (magenta) immunofluorescence colocalization with LPO neurons (green); 91.7% colocalized. Scale bars: 100 µm (left) and 10 µm (right). **B**, **C** Representative c-Fos in LPO from Sham, SNI, SNI+Saline, SNI + DEX; quantification (scale bar, 100 µm; *n* = 3 per group; one-way ANOVA with Tukey’s test, *F*(3,8) = 153.6; Sham vs. SNI, ****p < 0.0001; SNI vs. SNI + Saline, *p* = 0.1772; SNI + Saline vs. SNI + DEX, ****p < 0.0001). **D**, **E** AAV-DIO-EGFP into LPO of CaMKIIα-Cre after SNI; representative traces and quantification of LPO^Glu^ APs after saline or DEX (SNI + Saline, *n* = 10; SNI + DEX, *n* = 11; ordinary two-way ANOVA, *F*(1,19) = 7.623, *p = 0.0124). **F** Behavioral timeline. **G** Viral strategy and representative images for LSD^GABA^→LPO^Glu^ targeting (AAV2/1-CaMKIIα-Cre in LSD, AAV-DIO-hM4Di-mCherry in LPO, LSD cannula for DEX). Scale bar, 100 µm. **H** PWT after intra-LSD-saline or DEX followed by saline or CNO (*n* = 6; two-way ANOVA with Sidak’s test: interaction *F*(1,10) = 10.29, *p* = 0.0094; LSD-DEX saline vs. CNO, **p = 0.0017; LSD-saline vs. LSD-DEX under saline, ****p < 0.0001; under CNO, **p = 0.0092). **I**, **J** AAV-DIO-EGFP into LPO of GAD2-Cre after SNI; representative traces and LPO^GABA^ action potentials quantification after saline or DEX (SNI+Saline, *n* = 14; SNI + DEX, *n* = 14; ordinary two-way ANOVA, *F*(1,26) = 5.306, *p = 0.0295). **K** Behavioral timeline. **L** LSD^GABA^ → LPO^GABA^ targeting strategy; AAV2/1-VGAT-Cre into LSD, AAV-DIO-hM4Di-mCherry in LPO, LSD cannula for DEX. Scale bar, 100 µm. **M**–**O** Wake, NREM, REM after intra-LSD-saline or DEX followed by saline or CNO (*n* = 6; two-way ANOVA with Sidak’s test. Wake: interaction *F*(1,10) = 14.86, *p* = 0.0032; LSD-DEX saline vs. CNO, ***p = 0.0006; LSD-saline vs. LSD-DEX under saline, ****p < 0.0001; under CNO, ****p < 0.0001. NREM: interaction *F*(1,10) = 6.507, *p* = 0.0288; LSD-DEX saline vs. CNO, **p = 0.0071; LSD-saline vs. LSD-DEX under saline, ****p < 0.0001; under CNO, ****p < 0.0001. REM: interaction *F*(1,10) = 0.1666, *p* = 0.6918; LSD-saline saline vs. CNO, *p* = 0.9489; LSD-DEX saline vs. CNO, *p* = 0.6442; LSD-saline vs. LSD-DEX under saline, *p* = 0.6376; under CNO, *p* = 0.9636). Data were mean ± SD. All tests two-sided. Source data are provided as a Source Data file.

Both peer research and our results indicate that α_2_A adrenergic receptors are also expressed in LPO^21^, with quantitative analysis showing that 91.7% of LPO neurons colocalize with α_2_A receptors (Fig. 9A). To clarify that dexmedetomidine mediates comorbidity by regulating LPO activity through the α_2_A adrenergic receptor in LSD, we first examined c-Fos expression in the LPO across four experimental groups (Sham, SNI, SNI + Saline, and SNI + DEX). The results showed that SNI reduced LPO c-Fos expression compared with sham controls, whereas dexmedetomidine treatment increased LPO c-Fos expression relative to the saline-treated SNI group (Fig. 9B, C). Moreover, whole-cell patch-clamp recordings in GAD2-Cre and CaMKIIα-Cre mice injected with AAV-DIO-EGFP in LPO revealed that intra-LSD dexmedetomidine administration significantly enhanced firing rates in both LPO^GABA^ and LPO^Glu^ (Fig. 9D, E, I, J). To investigate whether the analgesic effect of dexmedetomidine involves the LSD^GABA^→LPO^Glu^ circuit, we injected AAV2/1-CaMKIIα-Cre into the LSD and Cre-dependent AAV-DIO-hM4Di-mCherry into the LPO of SNI mice. After intra-LSD dexmedetomidine alleviated mechanical allodynia, CNO was administered to inhibit downstream LPO glutamatergic neurons. This manipulation reduced the mechanical withdrawal threshold, indicating attenuation of the analgesic effect of dexmedetomidine (Fig. 9F–H). We next investigated whether the sleep-promoting effect of dexmedetomidine involves the LSD^GABA^ → LPO^GABA^ circuit. In SNI mice injected with AAV2/1-VGAT-Cre into the LSD and Cre-dependent AAV-DIO-hM4Di-mCherry into the LPO, CNO was administered after intra-LSD dexmedetomidine had improved sleep disturbance. Following CNO administration, wakefulness increased, and NREM sleep decreased, indicating attenuation of the sleep-promoting effect of dexmedetomidine (Fig. 9K–O). These results suggest that dexmedetomidine improves pain and sleep disruption through the LSD^GABA^→LPO^Glu^ and LSD^GABA^ → LPO^GABA^ circuits, respectively (Supplementary Fig. 8).

## Discussion

More than half of chronic pain patients complain of insufficient sleep, yet effective treatments remain elusive due to poorly understood neural mechanisms. Our study reveals that hyperexcitability of LSD^GABA^ neurons drives comorbid chronic pain and sleep disruption in SNI mice, characterized by mechanical allodynia and disrupted sleep architecture (increased wakefulness, decreased NREM and REM sleep) at 21–28 days post-SNI. Importantly, application of a sub-sedative dose of dexmedetomidine alleviates these comorbid symptoms by reducing LSD^GABA^ excitability via α_2_A adrenergic receptors. Moreover, we dissected the neural circuit of LSD^GABA^ and found that LSD^GABA^ can project to the LPO^Glu^ and LPO^GABA^. Dexmedetomidine alters the activity of LSD^GABA^→LPO^Glu^ circuit and the LSD^GABA^ → LPO^GABA^ circuit by acting on the LSD α_2_A adrenergic receptor to improve the chronic pain and sleep disruption, respectively. Taken together, our findings suggested that the comorbidity behavior-relieving effects of dexmedetomidine are indeed mediated by the LSD^GABA^→LPO^Glu^ circuit and the LSD^GABA^ → LPO^GABA^ circuit via the LSD^GABA^ α_2_A adrenergic receptor.

The lateral septum (LS), a basal forebrain structure, integrates abundant cortical and subcortical inputs and projects to multiple downstream regions to shape appropriate behavioral responses. Histologically, the LS can be divided into dorsal, intermediate, and ventral subdivisions (LSD, LSI, and LSV, respectively)^22,23^. Previous studies have implicated the LS in a wide range of functions, including emotional, motivational, and spatial behaviors^24^. Importantly, different LS subregions may participate in distinct behavioral processes. For example, the LSD has been linked to reward processing^25–27^, reward-driven behaviors such as feeding^28–31^ and memory-related processes^9,32^.

Consistent with previous evidence that GABAergic neurons constitute the predominant neuronal population in the LS^33^, our findings further highlight LSD^GABA^ neurons as a key contributor to chronic pain/sleep disruption comorbidity. We found that nerve injury markedly increased c-Fos expression and action potential firing in LSD^GABA^ neurons (Fig. 2A, E–F), indicating that these neurons become hyperactive under comorbid conditions. In addition, loss- and gain-of-function experiments using chemogenetic and optogenetic approaches showed that altered activity of LSD^GABA^ neurons is both necessary and sufficient to drive mechanical allodynia and sleep disruption (Fig. 3 and Supplementary Fig. 4). Previous studies have shown that α_2_ adrenergic receptors are expressed in several brain regions, including the locus coeruleus, hypothalamus, and prefrontal cortex^34–36^. Among these regions, the locus coeruleus has been considered a major site through which α_2_ adrenergic receptors mediate the sedative actions of dexmedetomidine. In the present study, we found that LSD^GABA^ neurons also contain α_2_ adrenergic receptors (Fig. 5G), suggesting that the LSD may represent an additional brain site influenced by dexmedetomidine. Although dexmedetomidine has been shown to exert analgesic or sleep-promoting effects in separate experimental models^37–39^, chronic pain/sleep disruption comorbidity represents a more complex state, and whether dexmedetomidine can improve both components of this comorbidity has not been fully clarified. In the present study, we found that intraperitoneal injection of sub-sedative doses of dexmedetomidine can alleviate the comorbidity behavior (Fig. 1G–L). Importantly, intra-LSD injection of dexmedetomidine can inhibit the LSD^GABA^ neurons hyperactivity and alleviated the comorbidity behavior (Figs. 4 and 5A–F). Furthermore, pre-injection of α_2_ adrenergic receptor antagonist yohimbine or special knockdown of α_2_A adrenergic receptor by intra-LSD injection of AAV-VGAT-α_2_A-shRNA both prevented the alleviating effect of dexmedetomidine on the comorbidity behavior (Fig. 5H–R). These findings suggest that dexmedetomidine improves chronic pain/sleep disruption comorbidity, at least in part, by reducing α_2_A adrenergic receptor-associated hyperactivity of LSD^GABA^ neurons. Notably, although morphine (5 mg/kg, i.p.) and meloxicam (1 mg/kg, i.p.) effectively alleviated mechanical allodynia, neither improved sleep disturbances in the comorbidity model (Supplementary Fig. 1D–G). This contrast highlights the dual ameliorative effect of dexmedetomidine on pain and sleep disruption and suggests that chronic pain and comorbid sleep disruption may involve partially distinct neural mechanisms.

The execution of complex behaviors relies on coordinated activity across distributed neural networks rather than on isolated brain regions^40^. The LS is anatomically positioned as a key limbic–hypothalamic interface, receiving hippocampal inputs and projecting to multiple downstream targets involved in affective, motivational, homeostatic, and arousal-related regulation, including hypothalamic, preoptic, mesolimbic, and brainstem-associated structures^10,22,41^. Importantly, this output organization is not uniform across LS subregions. The LSD preferentially communicates with reward- and homeostasis-related regions, including the VTA, NAC, LHA, MHA, and LPO^7,42^, whereas the LSI and LSV mainly project to the MPO and other medial hypothalamic regions^10,43^. Given this subregional and projection-specific organization, LSD-originating circuits may provide a structural basis for distinct behavioral regulation. Although the LS → LHA circuit has been reported to regulate pain–anxiety comorbidity^44^, whether and how LSD-originating circuits contribute to chronic pain and sleep disruption comorbidity remains unclear.

The LPO represents a particularly relevant downstream target because it contains both GABAergic neurons (LPO^GABA^) and glutamatergic neurons (LPO^Glu^)^45^. Previous work has shown that LPO^Glu^ neurons projecting to the VTA participate in the processing and relay of nociceptive information^46^. Consistent with this concept, our results showed that LPO^Glu^ neurons contributed to mechanical allodynia but did not significantly affect sleep architecture (Fig. 7). Furthermore, combining the virus trace, the Ca^2+^ signal assay and electrophysiology, we identified two projections from LSD^GABA^→LPO^Glu^ and LSD^GABA^ → LPO^GABA^. Activation of LSD^GABA^→LPO^Glu^ by chemogenetic and optogenetic methods prevented the mechanical allodynia, and inhibition of LSD^GABA^→LPO^Glu^ decreased the mechanical withdrawal threshold in naïve mice (Fig. 7 and Supplementary Fig. 5). These findings suggest that the circuit of LSD^GABA^→LPO^Glu^ mediated the painful behavior in the setting of comorbidity induced by nerve injury. As for LPO^GABA^ and the circuit of LSD^GABA^ → LPO^GABA^, we first found that nerve injury can decrease the excitability of LPO^GABA^. In addition, the activity of LPO^GABA^ was manipulated through chemical and optogenetic techniques to regulate sleep status, but it did not affect mechanical hyperalgesia. Furthermore, the role of the LSD^GABA^ → LPO^GABA^ circuit were also identified in comorbidity behavior. The loss and gain of the circuit function confirmed that the role of LSD^GABA^ → LPO^GABA^ circuit participated in the sleep disruption rather than the chronic pain in the comorbidity (Fig. 8 and Supplementary Fig. 6). Importantly, dexmedetomidine application prevented the LSD c-Fos upregulation and the LPO c-Fos downregulation induced by SNI, and intra-LSD injection of dexmedetomidine enhanced the activity of LPO^GABA^ and LPO^Glu^. Moreover, intra-LSD injection of dexmedetomidine reversed the mechanical allodynia induced by the inhibition of LPO^Glu^ by chemogenetic methods. Similarly, microinjection DEX into the LSD improved the chemogenetic-mediated sleep disruption (Fig. 9).

In summary, our study dissected two LSD-originating neural circuits. In the rodent model of SNI, the circuit of LSD^GABA^→LPO^Glu^ mediated the painful behavior of comorbidity symptoms induced by nerve injury. Meanwhile, the LSD^GABA^ → LPO^GABA^ circuit participated in the sleep disruption in comorbid mice. Importantly, DEX improved comorbidity behaviors through two loops, the LSD^GABA^**→**LPO^Glu^ circuit and the LSD^GABA^ → LPO^GABA^ circuit, respectively.

## Methods

### Animals

About 7–8 weeks of age male mice, C57BL/6J wild-type mice, were purchased from the Institute of Experimental Animals of Sun Yat-sen University. The GAD2-Cre mice and CaMKIIα-Cre mice were graciously provided by colleagues in Southern Medical University. The genotype of the mice was verified. The animals were housed at the SPF laboratory animal room on a 12-h light-dark cycle (7:00 a.m. to 7:00 p. m.) with adequate food and water in a stable environment (temperature, 23−25 °C, humidity 30−70%), and all behavioral experiments were performed during the light cycle. All procedures were performed in accordance with standard ethical guidelines in accordance with the National Institutes of Health Guide for the Care and Use of Laboratory Animals and were approved by the Animal Care and Use Committee of Sun Yat-sen University (approval number SYSU-IACUC-2024-000851). All Efforts were made to minimize animal suffering and to reduce the number of mice used according to the “3R principle”.

### Spared nerve injury (SNI)

The spared nerve injury (SNI) model was used to establish a comorbidity model of chronic pain and sleep disruption. The spared nerve injury (SNI) model is one of the most widely used and reliable models for studying neuropathic pain^47^. In brief, mice were anesthetized using 2% isoflurane and placed on a thermostatically controlled heating pad to maintain normothermia. Anesthesia was maintained with 1% isoflurane throughout the surgical procedure. A skin incision was made on the lateral aspect of the left thigh, and the underlying musculature was bluntly dissected to reveal the sciatic nerve and its three distal branches: the tibial, common peroneal, and sural nerves. The tibial and common peroneal nerves were tightly ligated with 4-0 silk sutures along a 2 mm segment and subsequently transected between the ligation points. Care was taken to leave the sural nerve undisturbed. After repositioning the nerve stumps, the muscle and skin layers were closed separately with sutures. In sham-operated mice, the same surgical exposure was performed without nerve ligation or transection.

### Electroencephalogram/electromyogram (EEG/EMG) recording

Mice were anesthetized with 1% sodium pentobarbital and placed in a stereotaxic frame. Body temperature was maintained using a heating pad. After scalp disinfection and midline incision, the skull was exposed, and electrode positions were marked (±1.0 mm AP from lambda, ±1.5 mm ML). Burr holes were drilled without damaging the dura. The EEG electrode board was fixed with screws, EMG wires were inserted into bilateralneck muscles, and the assembly was secured with tissue adhesive and dental cement. Mice recovered for one week before recording. EEG/EMG recordings were performed for 6 h during the light phase (09:00–15:00; ZT2–ZT8; lights on at 07:00) under a 12 h/12 h light/dark cycle.

Prior to data collection, mice were habituated to the recording environment for three days. EEG and EMG signals were acquired via a six-channel head-stage (2 EEG, 2 EMG, 1 reference over the contralateral cerebellum, and 1 ground) connected to the Intan RHD2000 system at 1.0 kS/s.

Data were processed in MATLAB. Signals were down-sampled to 500 Hz and low-pass filtered. Power spectral density (0–30 Hz) was computed using a 1-s Hamming window with 0.5-s steps via FFT and normalized to the peak value in each epoch. EEG power in the delta (0.5–4 Hz) and theta (4–8 Hz) bands, and EMG power in the 100–500 Hz range, were used to classify vigilance states in 4-s epochs: NREM sleep: high delta, low EMG, low theta/delta ratio, REM sleep: high theta/delta ratio, low delta, low EMG, Wakefulness: high EMG, high theta/delta ratio, low delta^48,49^.

### Stereotaxic surgery and viral injection

Mice were anesthetized with 1% sodium pentobarbital and secured in a stereotaxic frame. After scalp exposure and skull leveling based on bregma, target brain regions were identified using the Paxinos and Franklin atlas (LSD: AP + 0.50 mm, ML ± 0.35 mm, DV − 2.75 mm; LPO: AP − 0.02 mm, ML ± 0.98 mm, DV − 5.30 mm). Burr holes were drilled above the target sites.

For virus injection, 150–250 nL was delivered at 25 nL/min using a glass micropipette; the pipette was left in place for 10 min before withdrawal. Optical fibers or guide cannulas were then implanted at the target sites (same or 0.1 mm above injection depth) and fixed with skull screws and dental cement. Mice recovered for at least 1 week before behavioral or recording experiments. All viral vectors used in this study, including serotype, promoter, and source, are detailed in Supplementary Table 1.

### Optogenetic manipulation

To achieve region-specific optogenetic modulation, Cre-dependent viral vectors (AAV-DIO-eNpHR-mCherry, AAV-DIO-ChR2-mCherry, or AAV-DIO-ChrimsonR-mCherry) were injected into the target brain regions, followed by implantation of optical fibers and EEG/EMG electrodes. After full recovery, mice were connected to EEG/EMG recording cables and optical patch cords (1 m long, 200 μm core diameter) and habituated to the setup in the behavioral chamber for at least 3 consecutive days.

For light delivery, the optical fibers were connected to laser sources emitting blue (473 nm), yellow (593 nm), or red (635 nm) light. Light pulse trains (20 Hz) with pulse widths of 10 ms (blue/yellow) or 5 ms (red) were delivered with calibrated power at the fiber tip (2–5 mW), depending on the opsin used. For activation of GABAergic neurons in the dorsal lateral septum, 473-nm pulses were applied during stable NREM or REM sleep (30 s per trial, at 10–15 min intervals) to promote wakefulness, whereas 593-nm pulses were delivered during stable wakefulness (120 s per trial, at 20–30 min intervals) to induce NREM-like sleep. Red light (635 nm, 10 mW, 5 ms pulses, 20 Hz, and 20 s) was used as required for other opsins. During stimulation, EEG/EMG signals were continuously monitored in real time. EEG/EMG signals were scored into wake, NREM, or REM in 4-s epochs. For stimulation-aligned analyses, each trial was segmented into baseline (−120–0 s), laser (0–30 s), and post (30–150 s) windows, and state occupancy was calculated as the percentage of epochs in each window. Following optogenetic intervention, pain-related or sleep-related behavioral assessments were conducted.

### Fiber photometry

GCaMP6s was injected into the target brain region, followed by optical fiber implantation (200 μm, 0.37 NA, Inper, Hangzhou, China). Mice were habituated to the fiber patch cable in a behavioral chamber for 3 days prior to recording. For combined EEG/EMG experiments, mice were also adapted to the headstage wiring.

Recordings were conducted using a fiber photometry system (Inper, Hangzhou, China) equipped with dual-wavelength LEDs (470 nm, 25 μW; 410 nm, 15 μW). Signals were acquired at 60 frames per second with a 15 ms exposure time. A 10-min baseline was first recorded under spontaneous conditions, followed by calcium signal acquisition during pain stimulation or sleep–wake transitions.

Fluorescence signals were analyzed using custom MATLAB scripts. Raw signals were baseline-corrected, denoised, and low-pass filtered. The 410 nm signal served as an isosbestic reference to correct the 470 nm signal. ΔF/F was calculated as (F − F₀)/F₀, where F is the signal at each time point, and F₀ is the baseline fluorescence. Event-aligned ΔF/F values were averaged across trials and animals, and visualized using heatmaps or mean trace plots. Shaded areas indicate SD.

### Combined optogenetics and GCaMP imaging

To investigate the functional connectivity of the LSD^GABA^ → LPO^GABA^/ LPO^Glu^ circuit, we employed a multi-virus strategy. AAV-VGAT-ChrimsonR-mCherry and a monosynaptic anterograde tracer (AAV2/1-VGAT-Cre or AAV2/1-CaMKIIα-Cre) were injected into the LSD, while a Cre-dependent calcium indicator (AAV-DIO-GCaMP6s) was injected into the LPO, where an optical fiber was also implanted. After three weeks of viral expression, mice were habituated to the recording setup for 3 days (30 min/day).

During the experiment, mice were placed in a transparent chamber and connected to a dual-function optogenetic-calcium imaging system(Inper, Hangzhou, China). Calcium signals were recorded using dual-wavelength excitation (470 nm, 25 μW; 410 nm, 15 μW) at 60 Hz with 15 ms exposure. Red light stimulation (635 nm, 20 Hz, 5 ms pulse width, 10 mW, 20 s) was delivered through the optical fiber to activate ChrimsonR-expressing terminals in the LPO, while GCaMP6s fluorescence dynamics of LPO^GABA^ or LPO^Glu^ neurons receiving LSD^GABA^ input were recorded simultaneously.

### Chemogenetic manipulation

To manipulate specific neuronal populations in the LSD–LPO circuit, various chemogenetic strategies were employed. AAV-DIO-hM3Dq-mCherry or AAV-DIO-hM4Di-mCherry was injected into the LSD of GAD2-Cre mice or the LPO of CaMKIIα-Cre or GAD2-Cre mice. Mice were randomly assigned to receive clozapine-*N*-oxide (CNO; 1 mg/kg, i.p.) or an equivalent volume of saline. Pain and sleep-related behavioral assessments were conducted 20 min after injection.

To selectively inhibit LPO neurons receiving LSD input, AAV2/1-VGAT-Cre or AAV2/1- CaMKIIα-Cre was injected into the LSD and bilateral AAV-DIO-hM4Di-mCherry or AAV-DIO-hM3Dq-mCherry in the LPO.

To examine whether dexmedetomidine improves chronic pain–associated sleep disturbances via the LSD–LPO circuit, C57BL/6J mice received AAV2/1-VGAT-Cre or AAV2/1-CaMKIIα-Cre into the LSD, and AAV-DIO-hM4Di-mCherry into the LPO. In both the LSD^GABA^ → LPO^GABA^ and LSD^GABA^→LPO^Glu^ groups, dexmedetomidine(2 μM, 200nl)  was microinjected into the LSD, followed 10 min later by CNO (1mg/kg, i.p.). Pain and sleep behaviors were subsequently evaluated to assess whether dexmedetomidine reverses the inhibitory effect of CNO by activating LPO^GABA^ or LPO^Glu^ neurons that receive LSD^GABA^ projections.

### Behavioral testing

Behavioral assessments were conducted after experimental treatments.

### Paw withdrawal threshold test (PWT)

Mechanical sensitivity was assessed using the up-down method^50^. Mice were habituated for 3 days (30 min/day) in mesh-floor chambers. On the test day, after 30 min acclimation, von Frey filaments (2.44–4.31) were applied to the lateral plantar surface (sural nerve area) for 3–4 s with ≥10 s intervals. Testing started with the 3.61 filament, and responses were recorded as positive (paw withdrawal, licking, or shaking) or negative. The 50% paw withdrawal threshold (PWT) was calculated using the formula: PWT (g) = (10^(*X*^_*f*_^+ *kδ*)^)/1000, where *X*_*f*_ is the log of the final filament, *de* is a value from standard tables, and *δ* is the mean log interval. Testing was done during the light phase (07:00–19:00) in a quiet environment. For experiments involving dexmedetomidine, PWT testing was conducted 1 h after administration and restricted to periods when mice were awake and behaviorally responsive.

### Open field test (OFT)

Mice were placed in a 50 × 50 × 40 cm open box with 16 equal squares under uniform lighting (50–100 lux). After 30 min habituation, each mouse was placed in the center, and behavior was recorded for 6 minutes (first minute acclimation, last 5 min for analysis).

Parameters included total distance, center time, and center entries. Tests were run between 9:00–11:00 a.m. under constant conditions (22 ± 1 °C, 50 ± 5% humidity, <50 dB noise). The chamber was cleaned with 75% ethanol between trials.

### Elevated plus maze (EPM)

The maze had a central platform (5 × 5 cm), two open arms, and two closed arms (35 × 5 cm, with 10 cm walls), elevated 55 cm above the floor. Mice were placed in the center facing an open arm, and activity was recorded for 6 minutes (last 5 min analyzed). The maze was cleaned with 75% ethanol between sessions to remove scent traces.

### Loss of righting reflex (LORR)

The sedative effect of dexmedetomidine was evaluated using the loss of righting reflex (LORR), a well-established behavioral surrogate for anesthetic-induced loss of consciousness in rodents, which has been shown to correlate closely with unconsciousness in humans^51,52^. Male C57BL/6J mice (8–10 weeks old) received intraperitoneal (i.p.) injections of dexmedetomidine at doses of 10, 25, or 50 μg/kg. Immediately after injection, each animal was placed into a rotating cylindrical chamber (rotation speed: 3 rpm). LORR was defined as the animal rolling onto its back and failing to attempt self-righting for at least 60 s. Animals were monitored continuously for a 15-min observation period following injection.

### Immunohistochemistry

Mice were anesthetized with 1% sodium pentobarbital and perfused transcardially with 0.9% saline, followed by 4% paraformaldehyde (PFA). Brains were post-fixed in 4% PFA overnight and dehydrated in 30% sucrose for 48 h. After embedding in OCT, coronal sections (20 μm) were prepared using a cryostat (LEICA CM1950) and collected in PBS for immunostaining.

Sections were washed in PBS, blocked at room temperature for 1 h, and incubated with primary antibodies overnight at 4 °C. After washing, sections were incubated with secondary antibodies for 1 h, washed again, and mounted. Fluorescent images were acquired using a fluorescence microscope equipped with a digital camera (Eclipse Ni-E, Nikon, Japan). Primary antibodies included: c-Fos (rabbit, 1:400; Cell Signaling Technology, #2250), CaMKIIα (mouse, 1:100; Abcam, ab22609), GABA (rabbit, 1:800; Sigma-Aldrich, A2052), and α_2_A-adrenergic receptor (rabbit, 1:200; Invitrogen, YG371526). Secondary antibodies included: Alexa Fluor 488 (against mouse, rabbit, or rat; 1:200; Jackson ImmunoResearch) and Cy3 (against mouse, rabbit, or rat; 1:400; Jackson ImmunoResearch). Detailed information on the antibodies used in this study is provided in Supplementary Table 2.

### Patch-clamp electrophysiology

Whole-cell patch-clamp recordings were performed using protocols adapted from previous studies from our laboratory^53,54^. Mice were deeply anesthetized with isoflurane and rapidly decapitated. Coronal brain slices containing the target regions were cut at a thickness of 300 μm using a vibrating microtome (VT1200s, Leica). Slicing was performed in ice-cold oxygenated dissection solution containing 2 mM KCl, 12 mM MgSO_4_, 1.3 mM NaH_2_PO_4_, 26 mM NaHCO_3_, 0.2 mM CaCl_2_, 10 mM dextrose, and 220 mM sucrose, continuously bubbled with 95% O_2_ and 5% CO_2_. After sectioning, slices were immediately incubated in artificial cerebrospinal fluid containing 126 mM NaCl, 3 mM KCl, 1.25 mM NaH_2_PO4, 2 mM CaCl_2_, 26 mM NaHCO_3_, 1 mM MgSO_4_, and 10 mM dextrose at 35 °C for 30 min before recording.

For whole-cell recordings, slices were transferred to a recording chamber and continuously perfused with oxygenated artificial cerebrospinal fluid. Recording pipettes with a resistance of 5–8 MΩ were filled with a potassium gluconate-based internal solution containing 130 mM potassium gluconate, 10 mM HEPES, 10 mM KCl, 10 mM Na-phosphocreatine, 0.5 mM Na-guanosine 5′-triphosphate (GTP), 0.2 mM EGTA, and 4 mM Mg-adenosine 5′-triphosphate (ATP). The pH was adjusted to 7.2–7.4, and osmolality was maintained at 280–290 mOsmL. Recordings were initiated after a stabilization period of at least 3 min and were performed at 28–30 °C. To assess neuronal excitability, action potentials were evoked by depolarizing current injections from 0 to 200 pA in 20 pA increments, with each step lasting 500 ms. Offline analysis was performed using Clampfit 10.4 (Axon Instruments Inc., USA).

The functional effects of hM4Di and hM3Dq were evaluated in fluorescently labeled LSD neurons using current-clamp recordings. Hyperpolarizing test pulses of −20 pA were applied every 20 s to track input resistance. Once the baseline membrane potential was stable, CNO (5 μM) was perfused for 5 min. Changes in membrane potential and input resistance were analyzed by comparing the final minute before CNO perfusion with the 4–5 min period after drug application.

Photostimulation was directed to the target region via the microscope, with light intensity set to the minimum level that reliably evoked postsynaptic responses. To confirm ChR2 function, mCherry-positive ChR2-expressing neurons in GAD2-Cre mice were visually identified and stimulated with blue-light pulses at 10 Hz with a pulse width of 5 ms. In selected experiments, eNpHR function was examined using continuous yellow-light illumination at 593 nm.

Optically evoked IPSCs were recorded under a whole-cell voltage-clamp configuration. Patch pipettes with a resistance of 2–4 MΩ were filled with a cesium-based internal solution containing 110 mM Cs_2_SO4, 0.5 mM CaCl_2_, 5 mM TEA-Cl, 2 mM MgCl_2_, 5 mM HEPES, 5 mM EGTA, and 5 mM ATP. The pH was adjusted to 7.2–7.3, and the osmolarity was 310 mOsmL. IPSCs were recorded at a holding potential of 0 mV during 473-nm photostimulation of ChR2-expressing LSD^GABA^ terminals in LPO^GABA^ or LPO^Glu^ slices.

Paired-pulse stimulation was used to evaluate short-term presynaptic release properties of LSD^GABA^ inputs. Optically evoked IPSCs were recorded at a holding potential of 0 mV with an interstimulus interval of 200 ms. The paired-pulse ratio was calculated as the mean amplitude of the second IPSC divided by that of the first IPSC (IPSC2/IPSC1).

Data were analyzed only from recordings with a series resistance <20 MΩ and an input resistance >100 MΩ. Recordings were discarded when input resistance varied by >15% or series resistance changed by >10% during acquisition.

### Statistical analysis

Animals were assigned to experimental or control groups by randomization. Behavioral testing and data analysis were conducted by investigators blinded to group allocation. Statistical analyses and graph preparation were performed using GraphPad Prism 8.2 (GraphPad Software Inc.). Data distribution and equality of variance were evaluated before selecting parametric tests, unless otherwise stated. Two-group comparisons were analyzed using paired or unpaired two-tailed Student’s *t*-tests, as appropriate. For experiments involving more than two groups or two independent variables, one-way or two-way analysis of variance (ANOVA) was used, followed by appropriate post hoc multiple-comparison tests. Data were shown as mean ± SD. Statistical significance was defined as *p* < 0.05, with significance levels denoted as *p < 0.05, **p < 0.01, ***p < 0.001, and ****p < 0.0001.

### Reporting summary

Further information on research design is available in the Nature Portfolio Reporting Summary linked to this article.

## Supplementary information

Supplementary Information

Reporting Summary

Transparent Peer Review file

## Source data

Source Data

## Data Availability

The data supporting the findings of this study are available in the article, supplementary information, and accompanying Source Data file. Source data are provided with this paper.
