# Supplementary material for: Lateral septum GABAergic neurons mediate the effects of dexmedetomidine on allodynia and sleep in a male mouse model of neuropathic pain
Source: Nat Commun. 2026 Jun 19;17:7725. doi: 10.1038/s41467-026-74217-3 (PMC13434266; doi:10.1038/s41467-026-74217-3)
Supplement: Supplementary file 1 [file 41467_2026_74217_MOESM1_ESM.pdf]

Supplementary Information

Title: Lateral septum GABAergic neurons mediate the effects of dexmedetomidine on allodynia and sleep in a male mouse model of neuropathic pain

Haiting Fan[1,#], Yingying Su[2,#], Jie Deng[1,#], Zhengkai Liang[1,#],Meiying Chen[2], Kaibin Wu[3], Peiwen Tang[3], Chen Wang[1], Deyi Kong[3], Ting Xu[2], Feixue Liang[3,6*],Wenjun Xin[2,4,5*], Yan Wu[1,*], Xia Feng[1,*]

1 Department of Anesthesiology, The First Affiliated Hospital, Sun Yat-sen University, Guangzhou 510080, China.
2 Guangdong Province Key Laboratory of Brain Function and Disease, Department of Physiology and Pain Research Center, Zhongshan Medical School, Sun Yat-sen University, 74 Zhongshan Road 2, Guangzhou 510080, China.
3 Guangdong-Hong Kong-Macao Greater Bay Area Center for Brain Science and Brain-Inspired Intelligence, School of Biomedical Engineering, Southern Medical University, Guangzhou, 510515, China.
4 School of Basic Medical Sciences, Guangzhou Medical University, Guangzhou, Guangdong 511436, China
5 Key Laboratory for Molecular Genetic Mechanisms and Intervention Research on High Altitude Disease of Tibet, Autonomous Region, School of Medicine, Xizang Minzu University, Xian Yang, Shanxi 712082, China
6 Guangdong Basic Research Center of Excellence for Integrated Traditional and Western Medicine for Qingzhi Diseases, Southern Medical University, Guangzhou, 510515, China.

[#] These authors contributed equally

* Corresponding Author

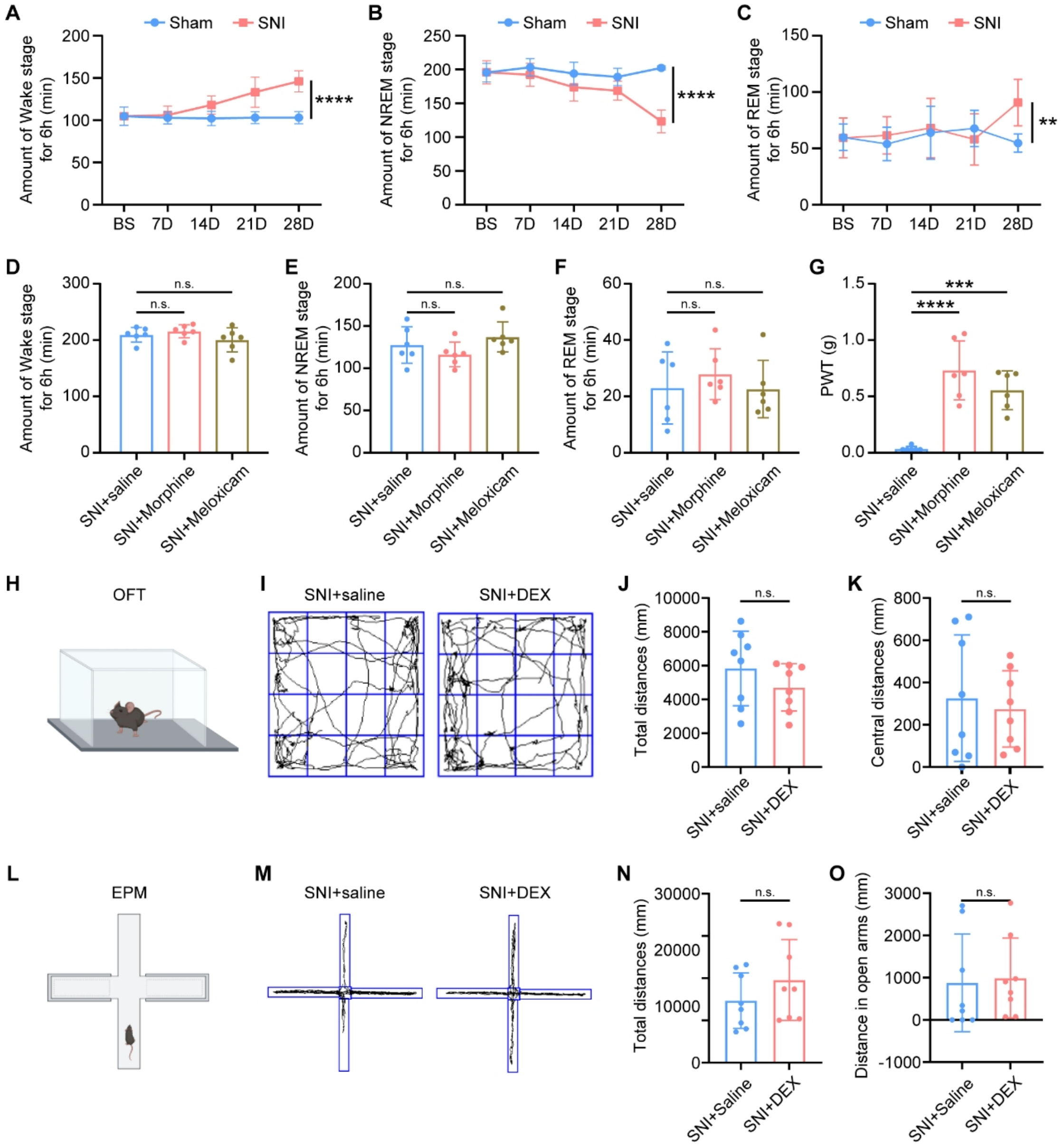

Supplementary Fig. 1 Dexmedetomidine's analgesic and sleep effects without motor impairment in SNI mice.

(A–C) Time-course of wake, NREM, and REM duration between Sham and SNI groups (Sham, n = 6; SNI, n = 6; two-way repeated-measures ANOVA with Bonferroni's test: Wake $F(4,50) = 8.378$, BS $p > 0.9999$; 7D $p > 0.9999$; 14D $p = 0.0670$; 21D ****$p < 0.0001$; 28D ****$p < 0.0001$. NREM $F(4,50) = 12.31$, BS $p > 0.9999$; 7D $p > 0.9999$; 14D $p = 0.1282$; 21D $p = 0.1202$; 28D ****$p < 0.0001$. REM $F(4,50) = 2.562$, BS $p > 0.9999$; 7D $p > 0.9999$; 14D $p > 0.9999$; 21D $p > 0.9999$; 28D **$p = 0.0077$). (D–G) Wake, NREM, REM, and PWT after saline, morphine (5 mg/kg) or meloxicam (10 mg/kg) treatment (n = 6 per group; ordinary one-way

ANOVA with Tukey's test: Wake $F(2,15) = 1.389$, Saline vs Morphine $p = 0.7848$; vs Meloxicam $p = 0.5953$. NREM $F(2,15) = 1.927$, Saline vs Morphine $p = 0.5564$; vs Meloxicam $p = 0.6434$. REM $F(2,15) = 0.4473$, Saline vs Morphine $p = 0.7180$; vs Meloxicam $p = 0.9976$. PWT $F(2,15) = 23.95$, Saline vs Morphine ****$p < 0.0001$; vs Meloxicam ***$p = 0.0005$). (H–K) Open field test (OFT) schematic (Created in BioRender. Liang, Z. (2026) https://BioRender.com/h8b4ut4 , unpaired t-test, total distance $t(14) = 1.216$, Saline, $n = 8$; DEX, $n = 8$; $p = 0.2442$. Center zone $t(14) = 0.4123$, $p = 0.6863$). (L–O) Elevated plus maze (EPM) schematic (Created in BioRender. Liang, Z. (2026) https://BioRender.com/h8b4ut4), representative trajectories, and movement analysis (Unpaired t-test, total distance $t(14) = 1.184$, Saline, $n = 8$; DEX, $n = 8$; $p = 0.2559$. Open arm $t(14) = 0.2199$, $p = 0.8291$). Data are mean ± SD. All tests two-sided. Source data are provided. BS, Baseline; OFT, open field test; EPM, elevated plus maze.

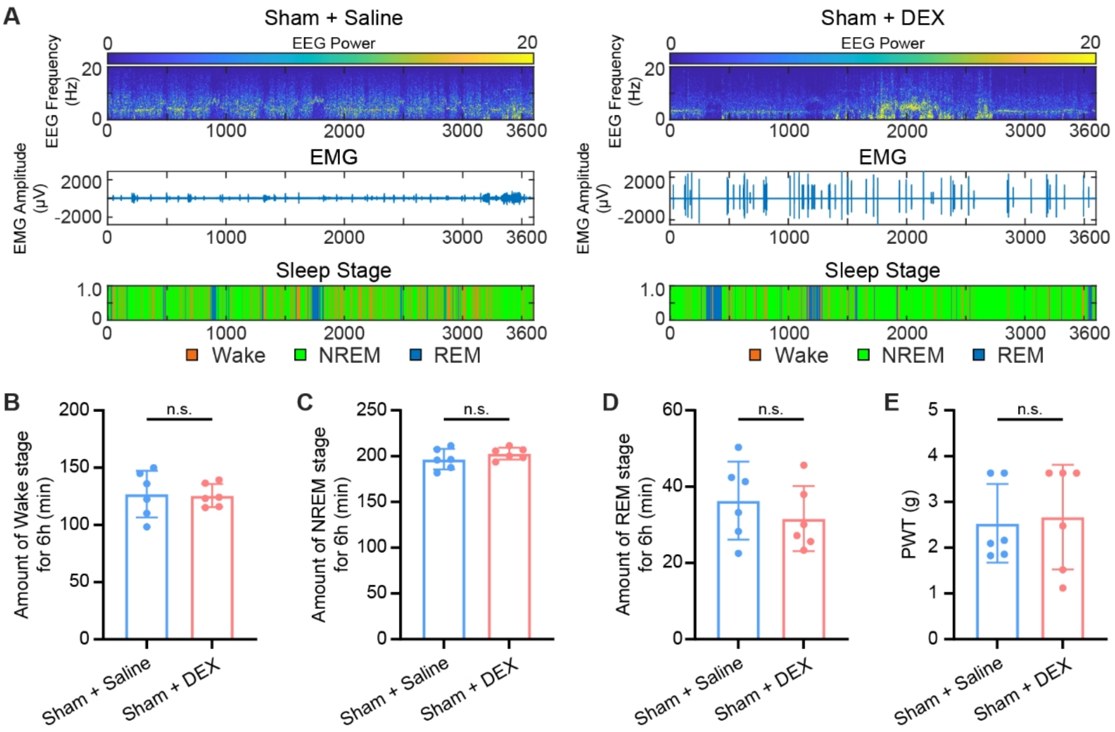

Supplementary Fig. 2 Dexmedetomidine does not alter sleep–wake architecture or mechanical pain sensitivity in sham-operated mice.

(A) Relative EEG power spectra (top) and representative EMG traces (middle) across

spontaneous sleep–wake states (bottom, color-coded, orange indicates wakefulness; green indicates NREM sleep; blue indicates REM sleep) in Sham groups treated with Saline and DEX. (B) Changes in wake duration following intraperitoneal injection of dexmedetomidine (25 µg/kg) or saline in sham-operated mice (n = 6 per group, unpaired t-test, $t(10) = 0.1413$, $p = 0.8905$). (C) Changes in NREM duration (n = 6 per group, unpaired t-test, $t(10) = 1.136$, $p = 0.2823$). (D) Changes in REM duration (n = 6 per group, unpaired t-test, $t(10) = 0.8660$, $p = 0.4068$). (E) Changes in PWT (n = 6 per group, Unpaired t-test, $t(10) = 0.2304$, $p = 0.8224$). Data are mean ± SD. All tests were two-sided. Source data are provided as a Source Data file.

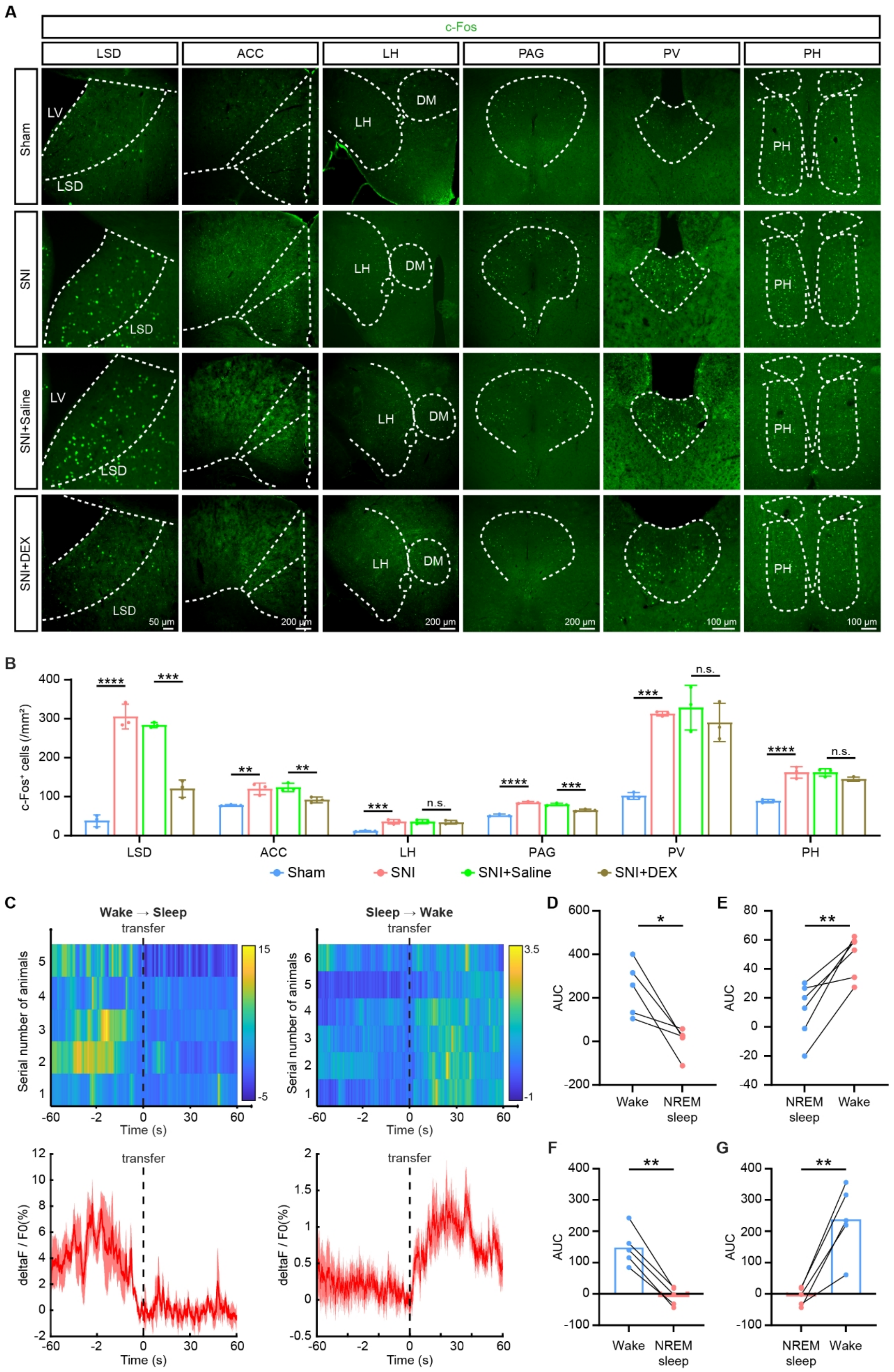

Supplementary Fig. 3 Regional c-Fos expression and LSD^{GABA} calcium transients.

(A) Representative c-Fos immunofluorescence images showing increased neuronal activation in LSD, ACC, LH, PAG, PV, PH in comorbid mice. Scale bar: 200 μm. (B) c-Fos expression was significantly increased in the SNI group compared to the Sham group in the LSD, ACC, LH, PAG, PV and PH regions (One-way ANOVA with Tukey's multiple comparisons test, LSD: $F(3, 8) = 113.9$, ****$p < 0.0001$; ACC: $F(3, 8) = 15.21$, **$p = 0.0016$; LH: $F(3, 8) = 20.02$, ***$p = 0.0004$; PAG: $F(3, 8) = 77.59$, ****$p < 0.0001$; PV: $F(3, 8) = 23.08$, ***$p = 0.0003$; PH: $F(3, 8) = 39.66$, ****$p < 0.0001$). For SNI+Saline vs SNI+DEX, significant differences were found in the LSD (***$p = 0.0006$), ACC (**$p = 0.0081$), and PAG (***$p = 0.0005$), while no significant differences were observed in the LH ($p = 0.8941$), PV ($p = 0.445$), or PH ($p = 0.1143$). (C) Left: heatmap(upper) and average Ca^{2+} transients (lower) of LSD^{GABA} neurons in naive mice during wake-to-sleep transitions (Sham, $n = 5$). Right: heatmap (upper) and average Ca^{2+} transients (lower) of LSD^{GABA} neurons in naive mice during sleep-to-wake transitions (Sham, $n = 6$). (D–E) Sham mice: AUC quantification of Ca^{2+} signals across vigilance-state transitions showed significant changes during the Wake→NREM transition (D; $n = 5$, paired t-test: $t(4) = 3.526$, *$p = 0.0243$) and the NREM→Wake transition (E; $n = 6$, paired t-test: $t(5) = 4.778$, **$p = 0.0050$). (F–G) SNI mice: AUC changed significantly during the Wake→NREM transition (F; $n = 5$, paired t-test: $t(4) = 8.474$, **$p = 0.0011$) and the NREM→Wake transition (G; $n = 5$, paired t-test: $t(4) = 5.678$, **$p = 0.0047$). Data are mean ± SD. All tests were two-sided. Source data are provided as a Source Data file. ACC, Anterior Cingulate Cortex; LH, Lateral Hypothalamus; PAG, Periaqueductal Gray; PV, Paraventricular nucleus of thalamus; PH, Posterior Hypothalamus.

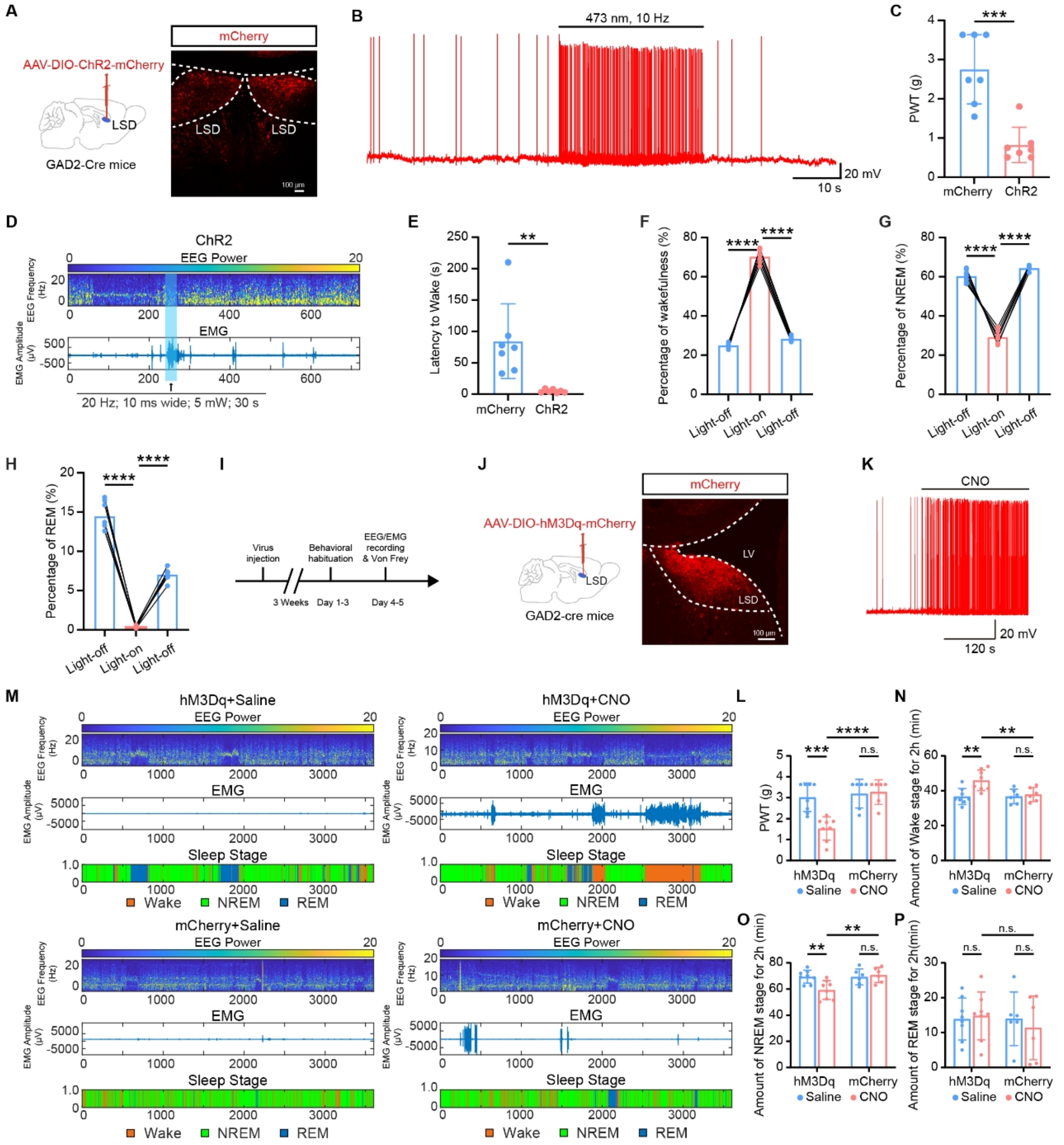

Supplementary Fig. 4 Activation of LSD^{GABA} neurons induced the mechanical allodynia and sleep disruption.

(A) ChR2 virus injection schematic and representative expression in LSD (Scale bars, 100 μm). (B) Whole-cell recordings showing increased firing during blue light (473 nm) in AAV-DIO-ChR2-expressing neurons in GAD2-Cre mice. (C) PWT after stimulation (n = 7, t(12) = 5.139, ***p = 0.0002). (D–H) Representative EEG/EMG traces, latency to Wake (E, n = 7, t(12) = 3.541, **p = 0.0041) and state occupancy quantified from EEG/EMG scoring in 4-s epochs across pre (−120–0 s), light-on (0–30 s), and post (30–150 s) windows: RM one-way ANOVA with Tukey: Wake F(1.022,6.132)=838.9; Light-off vs Light-on, ****p < 0.0001; Light-on vs post,

****$p < 0.0001$. NREM $F(1.036,6.216)=241.9$; Light-off vs Light-on, ****$p < 0.0001$; Light-on vs post, ****$p < 0.0001$. REM $F(1.219,7.314)=263.1$; Light-off vs Light-on, ****$p < 0.0001$; Light-on vs post, ****$p < 0.0001$. (I) Experimental timeline of behavioral assessments following chemogenetic activation of LSD^{GABA} neurons. (J) hM3Dq virus schematic and representative expression (Scale bars, 100 μm). (K) Whole-cell recordings showing CNO (5 μM) activation of LSD^{GABA} neurons. (L–P) PWT (L) and sleep–wake changes after saline or CNO (hM3Dq+Saline n=8; hM3Dq+CNO n=8; mCherry+Saline n=6; mCherry+CNO n=6), including EEG/EMG traces and hypnograms (M): Two-way ANOVA with Sidak: PWT $F(1,24)=10.75$, **$p=0.0032$; hM3Dq $F(1,24)=15.86$, ***$p=0.0006$; CNO $F(1,24)=8.743$, **$p=0.0069$; hM3Dq Saline vs CNO ***$p=0.0002$; mCherry Saline vs CNO $p=0.9722$; hM3Dq vs mCherry under Saline $p=0.8580$; under CNO ****$p<0.0001$. Wake $F(1,24)=4.883$; hM3Dq Saline vs CNO **$p=0.0016$; mCherry Saline vs CNO $p=0.9031$; hM3Dq vs mCherry under Saline $p=0.9995$; under CNO **$p=0.0098$. NREM $F(1,24)=6.336$; hM3Dq Saline vs CNO **$p=0.0052$; mCherry Saline vs CNO $p=0.8966$; hM3Dq vs mCherry under Saline $p=0.9989$; under CNO **$p=0.0035$. REM $F(1,24)=0.39$; hM3Dq Saline vs CNO $p=0.9630$; mCherry Saline vs CNO $p=0.7937$; hM3Dq vs mCherry under Saline $p=0.9998$; under CNO $p=0.6338$. Data are mean ± SD. All tests two-sided. Source data are provided as a Source Data file.

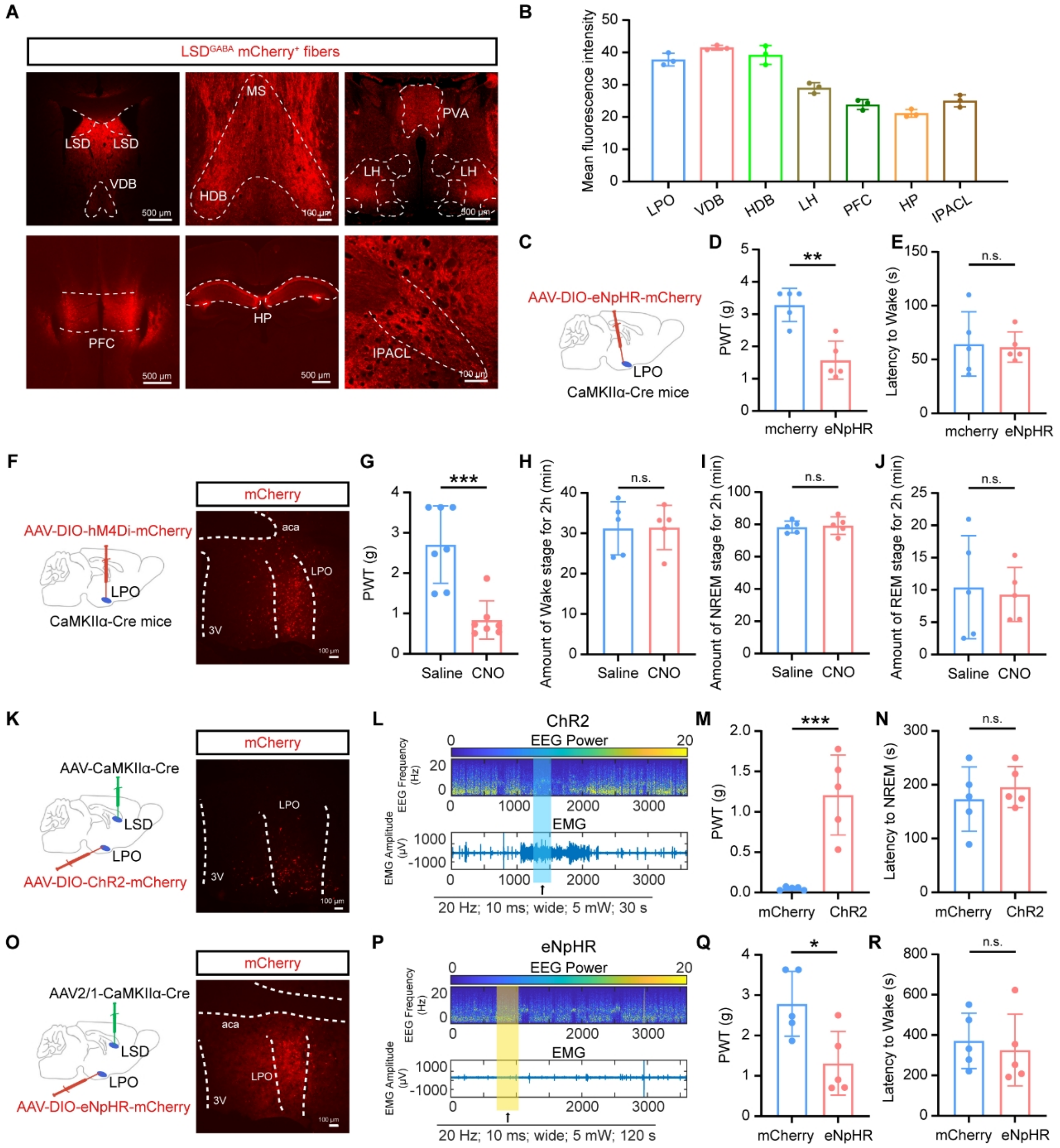

Supplementary Fig. 5 The LSD^{GABA}→LPO^{Glu} circuit contributed to the mechanical allodynia in comorbidity mice.

(A) LSD^{GABA} mCherry^{+} fiber distribution in downstream regions including VDB, HDB, PVA, LH, PFC, HP, and IPACL. (B) Quantification of fluorescence intensity of LSD^{GABA} projections. (C–E) AAV-DIO-eNpHR-mCherry in LPO schematic (C), PWT after stimulation (D, unpaired t-test, n = 5, $t(8)$ = 4.885, **p = 0.0012), and latency to Wake during NREM (E, unpaired t-test, n = 5, $t(8)$ = 0.1911, p = 0.8532). (F–H) AAV-DIO-hM4Di-mCherry injection schematic in LPO (F, Scale 100 μm), PWT after saline or CNO (G, unpaired t-test, Saline n = 7, CNO n = 7, $t(12)$ = 4.624, ***p = 0.0006), and wake/NREM/REM durations showing no significant differences between

groups (H–J, unpaired t-test, n = 5; Wake $t(8)$ = 0.05943, p = 0.9541; NREM $t(8)$ = 0.2951, p = 0.7755; REM $t(8)$ = 0.2729, p = 0.7919). (K–N) AAV2/1-CaMKIIα-Cre in LSD and AAV-DIO-ChR2-mCherry in LPO schematic and EEG/EMG traces (K–L), PWT after blue light stimulation (M, unpaired t-test, n = 5, $t(8)$ = 5.237, ***p = 0.0008), and latency to NREM during wake (N, unpaired t-test, n = 5, $t(8)$ = 0.6271, p = 0.5481). (O–R) AAV2/1-CaMKIIα-Cre in LSD and AAV-DIO-eNpHR-mCherry in LPO schematic and EEG/EMG traces (O–P), PWT after yellow light inhibition (Q, unpaired t-test, n = 5, $t(8)$ = 2.926, *p = 0.0191), and latency to wake (R, unpaired t-test, n = 5, $t(8)$ = 0.4496, p = 0.6649). Data are mean ± SD. All tests two-sided. Source data are provided as a Source Data file.

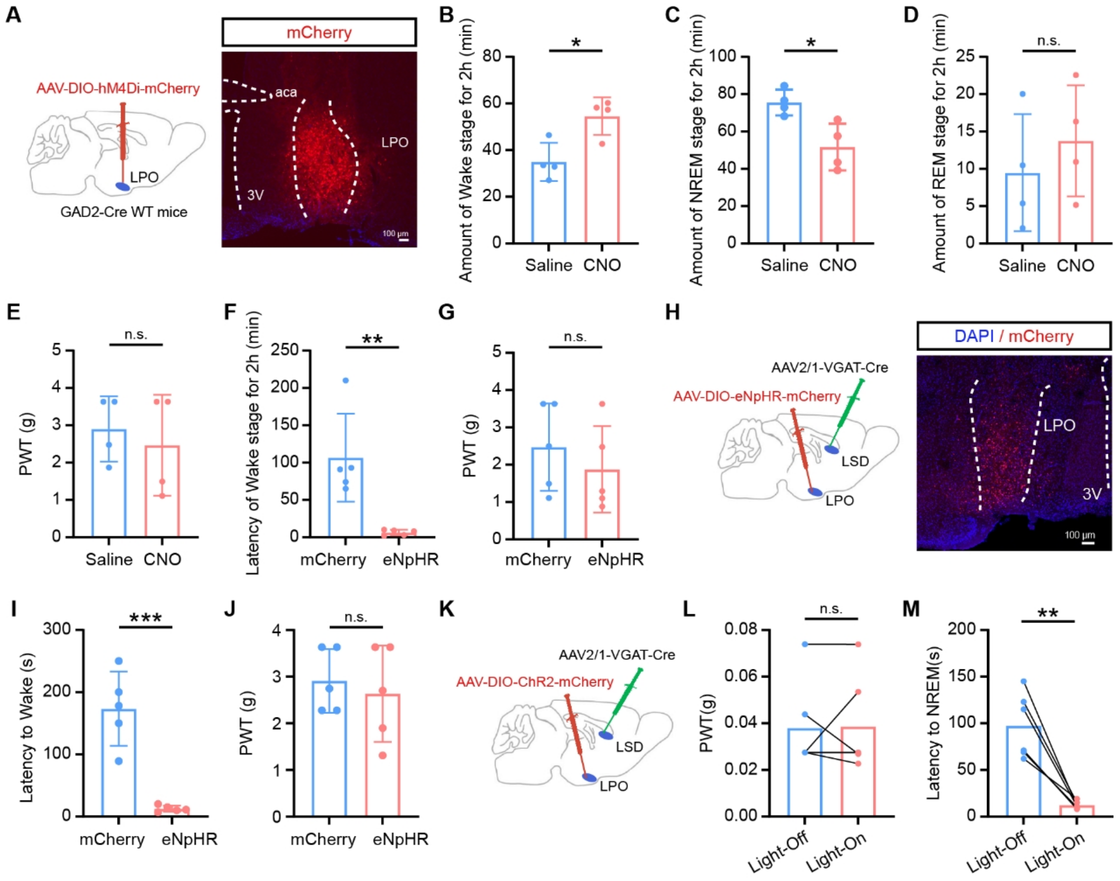

Supplementary Fig. 6 The LSD^{GABA}→LPO^{GABA} circuit contributed to the sleep disruption in comorbidity mice.

(A) Schematic and representative image of the AAV-DIO-hM4Di-mCherry injection into LPO in GAD2-Cre mice. Scale bar, 100 μm. (B)-(D) Wakefulness, NREM

durations showed significant differences between saline and CNO treatment groups (Unpaired t test, Saline, n = 4, CNO, n = 4, Wake: $t(6) = 3.429$, *$p = 0.0140$; NREM: $t(6) = 3.335$, *$p = 0.0157$; REM: $t(6) = 0.7871$, $p = 0.4612$). (E) Changes in PWT after saline or CNO injection (Unpaired t test, Saline, n = 4, CNO, n = 4, $t(6) = 0.5407$, $p = 0.6082$).(F) Changes in latency to wake stage after yellow lights inhibition (Saline, n = 5, CNO, n = 5, $t(8) = 3.792$, **$p = 0.0053$). (G) Changes in PWT after yellow lights inhibition (Unpaired t test, Saline, n = 5, CNO, n = 5, $t(8) = 0.8089$, $p = 0.4420$). (H) Schematic diagram and representative imaging (Scale bar, 100 μm) of AAV2/1-VGAT-Cre virus in LSD and AAV-DIO-eNpHR-mCherry in LPO. (I) Yellow light stimulation significantly reduced wakefulness onset latency in naive mice (Unpaired t test, n = 5, $t(8) = 5.848$, ***$p = 0.0004$). (J) No significant changes in in PWT after yellow lights inhibition (Unpaired t test, n = 5, $t(8) = 0.5008$, $p = 0.6300$). (K) Schematic diagram of AAV2/1-VGAT-Cre virus in LSD and AAV-DIO-ChR2-mCherry in LPO. (L) No significant changes in in PWT after blue lights activation (Paired t-test, n = 6, $t(5) = 0.1224$, $p = 0.9074$). (M) Blue light stimulation significantly reduced latency to NREM stage in SNI mice (Paired t test, n = 6, $t(5) = 5.917$, **$p = 0.0020$). Data are mean ± SD. All tests were two-sided. Source data are provided as a Source Data file.

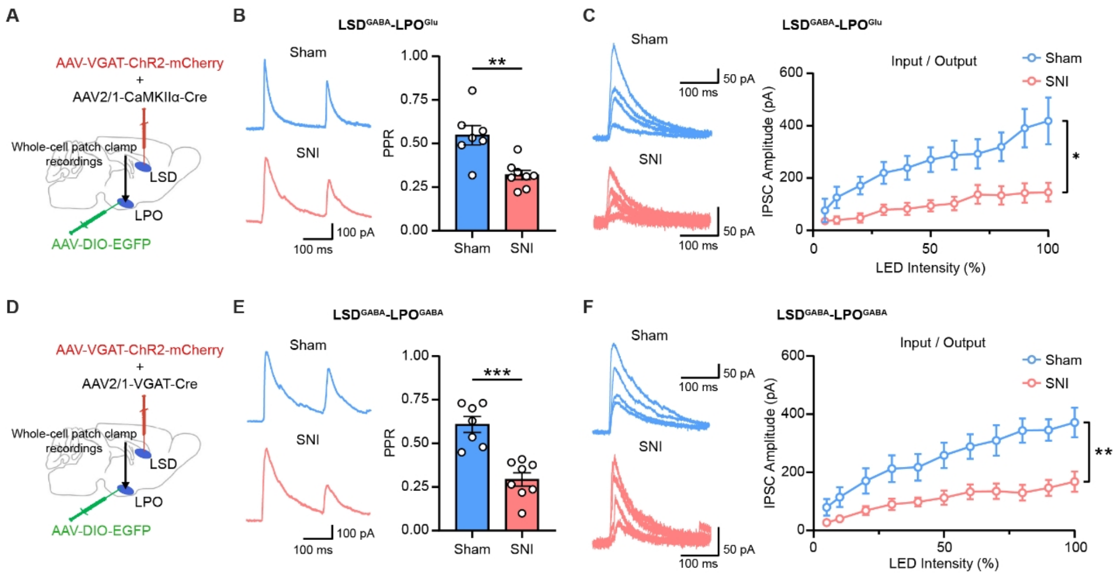

Supplementary Fig. 7 Synaptic properties of LSD GABAergic projections to LPO glutamatergic and GABAergic neurons.

(A) Schematic illustration of viral strategy targeting LSD^{GABA}–LPO^{Glu} projections and the experimental configuration for whole-cell patch-clamp recordings from LPO^{Glu} neurons. (B) Representative traces of paired-pulse ratio (PPR) of optogenetically evoked inhibitory postsynaptic currents (IPSCs) and corresponding quantification. (Sham, $n = 7$ cells; SNI, $n = 8$ cells, Unpaired t-test, $t(13) = 3.895$, $^{**}p = 0.0018$). (C) Input–output (I–O) curves and summary statistics of optogenetically evoked IPSC amplitudes. ($n = 6$ cells per group, Ordinary Two-way ANOVA, $F(1, 10) = 9.126$, $^{*}p = 0.0129$). (D) Schematic illustration of viral strategy targeting LSD^{GABA}–LPO^{GABA} projections and the experimental configuration for whole-cell patch-clamp recordings from LPO^{GABA} neurons. (E) Representative PPR traces of optogenetically evoked IPSCs and corresponding quantification. (Sham, $n = 7$ cells; SNI, $n = 8$ cells, Unpaired t-test, $t(13) = 5.362$, $^{***}p = 0.0001$). (F) Input–output (I–O) curves and summary statistics of optogenetically evoked IPSC amplitudes. (Sham, $n = 6$ cells; SNI, $n = 7$ cells, Ordinary Two-way ANOVA, $F(1, 11) = 11.84$, $^{**}p = 0.0055$). Data are mean ± SD. All tests were two-sided. Source data are provided as a Source Data file.

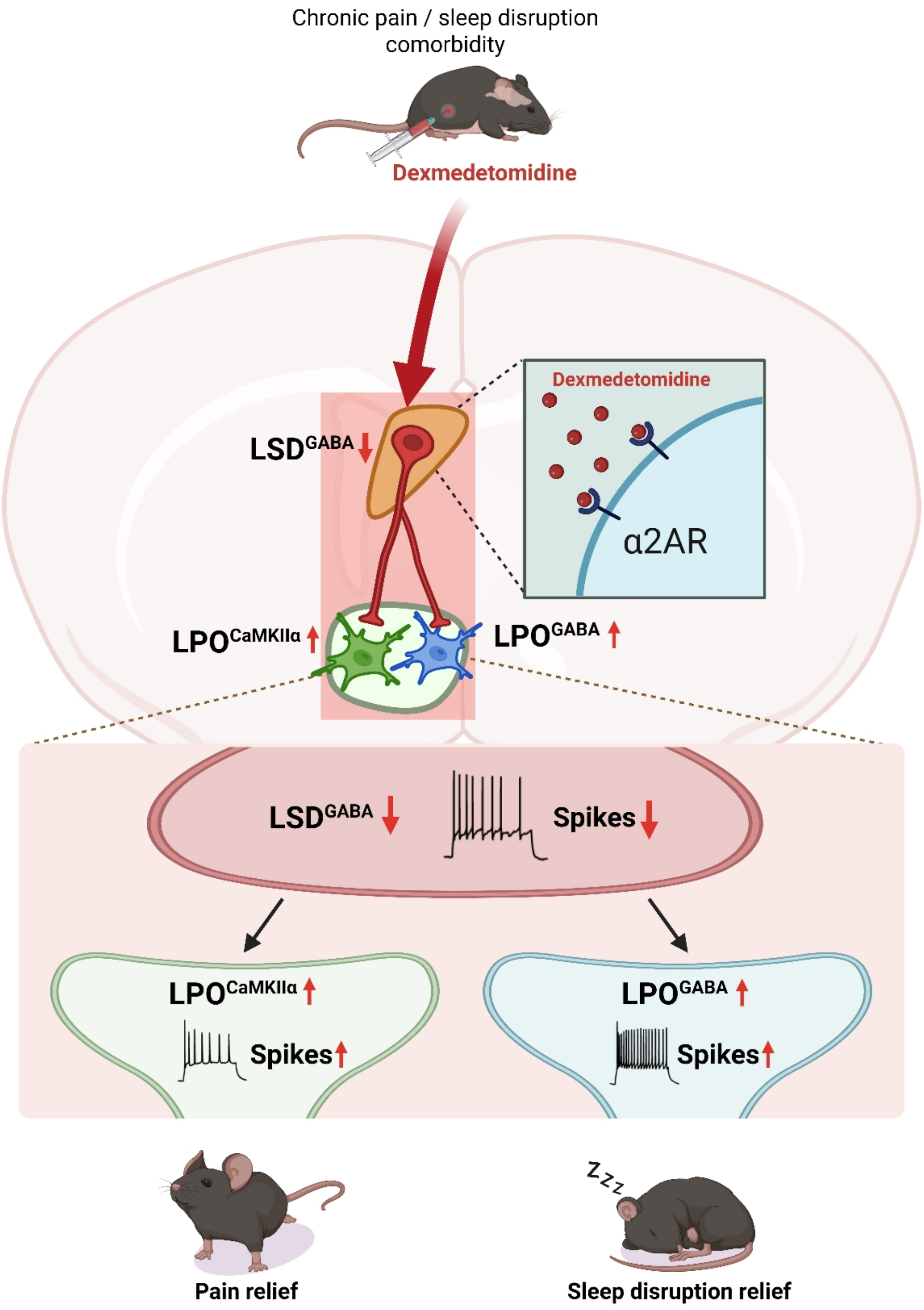

Supplementary Fig. 8 Graphical abstract

We discovered that systemic or local application of DEX reduced LSD^{GABA} hyperactivity via α_2A adrenergic receptor activation, alleviating comorbid pain and sleep disruption. Furthermore, LSD^{GABA} can project onto the glutamatergic neurons and GABAergic neurons in lateral preoptic area (LPO), respectively. The LSD^{GABA}→LPO^{Glu} circuit and the LSD^{GABA}→LPO^{GABA} circuit contributed to the

mechanical allodynia and the sleep disruption in comorbidity mice. Finally, the LSD α_2 adrenergic receptor play important role in DEX-regulated the activity of LPO^{GABA} and LPO^{Glu} (Created in BioRender. Liang, Z. (2026) https://BioRender.com/h8b4ut4).

Supplementary Table 1. Summary of Viral Vectors

No.	Virus Name	Serotype	Titer	Source
1	AAV-hSyn-Cre	AAV2/1	1.0×10^{13} vg/ml	BrainVTA
2	AAV-VGAT-Cre	AAV2/1	1.10×10^{13} vg/ml	BrainVTA
3	AAV-CaMKIIα-Cre	AAV2/1	1.0×10^{13} vg/ml	BrainVTA
4	AAV-VGAT- mCherry	AAV2/9	5.0×10^{12} vg/ml	BrainVTA
5	AAV-VGAT-GCaMP6s	AAV2/9	5.0×10^{12} vg/ml	BrainVTA
6	AAV-VGAT-ChrimsonR-mCherry	AAV2/9	5.34×10^{12} vg/ml	BrainVTA
7	AAV-VGAT-α2A-scramble	AAV2/9	5.0×10^{12} vg/ml	BrainVTA
8	AAV-VGAT-α2A-shRNA	AAV2/9	5.0×10^{12} vg/ml	BrainVTA
9	AAV-DIO-EGFP	AAV2/9	5.82×10^{12} vg/ml	BrainVTA
10	AAV-DIO-mCherry	AAV2/9	5.13×10^{12} vg/ml	BrainVTA
11	AAV-DIO-GCaMP6s	AAV2/9	5.0×10^{12} vg/ml	BrainVTA
12	AAV-DIO-ChR2-mCherry	AAV2/9	2.18×10^{13} vg/ml	OBiO
13	AAV-DIO-eNpHR-mCherry	AAV2/9	1.43×10^{13} vg/ml	OBiO
14	AAV-DIO-eNpHR-EYFP	AAV2/9	1.43×10^{13} vg/ml	OBiO
15	AAV-DIO-hM3Dq-mCherry	AAV2/9	5.0×10^{12} vg/ml	BrainVTA
16	AAV-DIO-hM4Di-mCherry	AAV2/9	5.0×10^{12} vg/ml	BrainVTA
17	CTB-555		1 μg/μ1	BrainVTA

Supplementary Table 2. Summary of Antibodies used

No.	Antibodies Name	Dilution	Identifier	Source
1	Rabbit anti-c-Fos	1:400	Cat# 2250, RRID: AB_2247211	Cell Signaling Technology
2	Mouse anti-CaMKIIα	1:100	Cat# ab22609, RRID: AB_447192	Abcam
3	Rabbit anti-GABA	1:800	Cat# A2052, RRID: AB_477652	Sigma-Aldrich
4	Rabbit anti-α2AR	1:200	Cat# PA1-048, RRID: AB_2225243	Invitrogen
5	Mouse anti-NeuN	1:200	Cat# MAB377, RRID: AB_2298772	Sigma-Aldrich
6	Alexa Fluor 488-AffiniPure Donkey Anti-Mouse IgG(H+L)	1:200	Cat# 715-545-150, RRID: AB_2340846	Jackson ImmunoResearch
7	Alexa Fluor 488-AffiniPure Donkey Anti-Rabbit IgG(H+L)	1:200	Cat# 711-545-152, RRID: AB_2313584	Jackson ImmunoResearch
8	Cy3-AffiniPure Donkey Anti-Mouse IgG(H+L)	1:400	Cat# 715-165-150, RRID: AB_2340813	Jackson ImmunoResearch
9	Cy3-AffiniPure Donkey Anti-Rabbit IgG(H+L)	1:400	Cat# 711-165-152, RRID: AB_2307443	Jackson ImmunoResearch
