# Supplementary material for: Lateral septum GABAergic neurons mediate the effects of dexmedetomidine on allodynia and sleep in a male mouse model of neuropathic pain
Source: Nat Commun. 2026 Jun 19;17:7725. doi: 10.1038/s41467-026-74217-3 (PMC13434266; doi:10.1038/s41467-026-74217-3)
Supplement: Supplementary file 2 [file 41467_2026_74217_MOESM2_ESM.pdf]

nature portfolio

Corresponding author(s): Feixue Liang,Wenjun Xin, Yan Wu, Xia Feng

Last updated by author(s): May 20, 2026

Reporting Summary

Nature Portfolio wishes to improve the reproducibility of the work that we publish. This form provides structure for consistency and transparency in reporting. For further information on Nature Portfolio policies, see our Editorial Policies and the Editorial Policy Checklist.

Statistics

For all statistical analyses, confirm that the following items are present in the figure legend, table legend, main text, or Methods section.

n/a	Confirmed	
☐	☒	The exact sample size (*n*) for each experimental group/condition, given as a discrete number and unit of measurement
☐	☒	A statement on whether measurements were taken from distinct samples or whether the same sample was measured repeatedly
☐	☒	The statistical test(s) used AND whether they are one- or two-sided<br>*Only common tests should be described solely by name; describe more complex techniques in the Methods section.*
☒	☐	A description of all covariates tested
☐	☒	A description of any assumptions or corrections, such as tests of normality and adjustment for multiple comparisons
☐	☒	A full description of the statistical parameters including central tendency (e.g. means) or other basic estimates (e.g. regression coefficient) AND variation (e.g. standard deviation) or associated estimates of uncertainty (e.g. confidence intervals)
☐	☒	For null hypothesis testing, the test statistic (e.g. *F*, *t*, *r*) with confidence intervals, effect sizes, degrees of freedom and *P* value noted<br>*Give P values as exact values whenever suitable.*
☒	☐	For Bayesian analysis, information on the choice of priors and Markov chain Monte Carlo settings
☒	☐	For hierarchical and complex designs, identification of the appropriate level for tests and full reporting of outcomes
☒	☐	Estimates of effect sizes (e.g. Cohen's *d*, Pearson's *r*), indicating how they were calculated

Our web collection on statistics for biologists contains articles on many of the points above.

Software and code

Policy information about availability of computer code

Data collection	For optical-fiber-based calcium signals recording, we used Inperstudio (Inper Ltd, China). For activity behaviors were videotaped using a video tracking system (Shanghai Xin Run. Ltd, China).EEG Recordings were used Intan RHD2000 system (USA).
Data analysis	For optical-fiber-based calcium signal recording, the data was analyzed using Inper Data Process (Inper Ltd, China). The activity behaviors were analyzed by software (Shanghai Jiliang Software Technology, Co., Ltd.).EEG signals were analyzed using standard MATLAB functions (MathWorks, R2022a). Statistical analyses were performed in GraphPad Prism.

For manuscripts utilizing custom algorithms or software that are central to the research but not yet described in published literature, software must be made available to editors and reviewers. We strongly encourage code deposition in a community repository (e.g. GitHub). See the Nature Portfolio guidelines for submitting code & software for further information.

Data

Policy information about availability of data

All manuscripts must include a data availability statement. This statement should provide the following information, where applicable:

- Accession codes, unique identifiers, or web links for publicly available datasets
- A description of any restrictions on data availability
- For clinical datasets or third party data, please ensure that the statement adheres to our policy

All the data generated in the current study are presented in the figures and supplementary materials. The relevant raw data are provided as a Source Data file. Source data are provided with this paper.

Research involving human participants, their data, or biological material

Policy information about studies with human participants or human data. See also policy information about sex, gender (identity/presentation), and sexual orientation and race, ethnicity and racism.

Reporting on sex and gender	*Use the terms sex (biological attribute) and gender (shaped by social and cultural circumstances) carefully in order to avoid confusing both terms. Indicate if findings apply to only one sex or gender; describe whether sex and gender were considered in study design; whether sex and/or gender was determined based on self-reporting or assigned and methods used. Provide in the source data disaggregated sex and gender data, where this information has been collected, and if consent has been obtained for sharing of individual-level data; provide overall numbers in this Reporting Summary. Please state if this information has not been collected. Report sex- and gender-based analyses where performed, justify reasons for lack of sex- and gender-based analysis.*
Reporting on race, ethnicity, or other socially relevant groupings	*Please specify the socially constructed or socially relevant categorization variable(s) used in your manuscript and explain why they were used. Please note that such variables should not be used as proxies for other socially constructed/relevant variables (for example, race or ethnicity should not be used as a proxy for socioeconomic status). Provide clear definitions of the relevant terms used, how they were provided (by the participants/respondents, the researchers, or third parties), and the method(s) used to classify people into the different categories (e.g. self-report, census or administrative data, social media data, etc.) Please provide details about how you controlled for confounding variables in your analyses.*
Population characteristics	*Describe the covariate-relevant population characteristics of the human research participants (e.g. age, genotypic information, past and current diagnosis and treatment categories). If you filled out the behavioural & social sciences study design questions and have nothing to add here, write "See above."*
Recruitment	*Describe how participants were recruited. Outline any potential self-selection bias or other biases that may be present and how these are likely to impact results.*
Ethics oversight	*Identify the organization(s) that approved the study protocol.*

Note that full information on the approval of the study protocol must also be provided in the manuscript.

Field-specific reporting

Please select the one below that is the best fit for your research. If you are not sure, read the appropriate sections before making your selection.

☒ Life sciences ☐ Behavioural & social sciences ☐ Ecological, evolutionary & environmental sciences

For a reference copy of the document with all sections, see nature.com/documents/nr-reporting-summary-flat.pdf

Life sciences study design

All studies must disclose on these points even when the disclosure is negative.

Sample size	Sample sizes were determined based on previous publications and common practice in animal behavior experiments.
Data exclusions	For all experiments, mice that displayed signs of infection, bleeding, or other unhealthy conditions after surgery were excluded from behavioral tests. Additionally, mice that had missed viral injections or implantation targets, as identified by brain atlas, were not included in the experimental analyses.
Replication	Experimental findings from all subjects across multiple experiments were reliably reproduced. Each subject was indicated in all figures and methods to ensure reproducibility.
Randomization	The animals in the behavioral tests were randomized assigned.
Blinding	All investigators were blinded to group allocation during data analysis.

Reporting for specific materials, systems and methods

We require information from authors about some types of materials, experimental systems and methods used in many studies. Here, indicate whether each material, system or method listed is relevant to your study. If you are not sure if a list item applies to your research, read the appropriate section before selecting a response.

Materials & experimental systems

n/a	Involved in the study
☐	☒ Antibodies
☒	☐ Eukaryotic cell lines
☒	☐ Palaeontology and archaeology
☐	☒ Animals and other organisms
☒	☐ Clinical data
☒	☐ Dual use research of concern
☒	☐ Plants

Methods

n/a	Involved in the study
☒	☐ ChIP-seq
☒	☐ Flow cytometry
☒	☐ MRI-based neuroimaging

Antibodies

Antibodies used

1 Rabbit anti-c-Fos 1:400 Cat# 2250, RRID: AB_2247211 Cell Signaling Technology,PMCID: PMC5760096
2 Mouse anti-CaMKIIα 1:100 Cat# ab22609, RRID: AB_447192 Abcam
3 Rabbit anti-GABA 1:800 Cat# A2052, RRID: AB_477652 Sigma-Aldrich
4 Rabbit anti-α2AR 1:200 Cat# PA1-048, RRID: AB_2225243 Invitrogen
5 Mouse anti-NeuN 1:200 Cat# MAB377, RRID: AB_2298772 Sigma-Aldrich
6 Alexa Fluor 488-AffiniPure Donkey Anti-Mouse IgG(H+L) 1:200 Cat# 715-545-150, RRID: AB_2340846 Jackson ImmunoResearch
7 Alexa Fluor 488-AffiniPure Donkey Anti-Rabbit IgG(H+L) 1:200 Cat# 711-545-152, RRID: AB_2313584 Jackson ImmunoResearch
8 Cy3-AffiniPure Donkey Anti-Mouse IgG(H+L) 1:400 Cat# 715-165-150, RRID: AB_2340813 Jackson ImmunoResearch
9 Cy3-AffiniPure Donkey Anti-Rabbit IgG(H+L) 1:400 Cat# 711-165-152, RRID: AB_2307443 Jackson ImmunoResearch

Validation

1 Rabbit anti-c-Fos Cat# 2250, RRID: AB_2247211 Cell Signaling Technology,PMCID: PMC5760096
2 Mouse anti-CaMKIIα Cat# ab22609, RRID: AB_447192 Abcam,PMID: 39721025
3 Rabbit anti-GABA Cat# A2052, RRID: AB_477652 Sigma-Aldrich,PMID: 39256434
4 Rabbit anti-α2AR Cat# PA1-048, RRID: AB_2225243 Invitrogen,PMID: 38242116
5 Mouse anti-NeuN Cat# MAB377, RRID: AB_2298772 Sigma-Aldrich,PMID: 28197125
6 Alexa Fluor 488-AffiniPure Donkey Anti-Mouse IgG(H+L) Cat# 715-545-150, RRID: AB_2340846 Jackson ImmunoResearch,PMID: 36778241
7 Alexa Fluor 488-AffiniPure Donkey Anti-Rabbit IgG(H+L) Cat# 711-545-152, RRID: AB_2313584 39325627
8 Cy3-AffiniPure Donkey Anti-Mouse IgG(H+L) Cat# 715-165-150, RRID: AB_2340813 Jackson ImmunoResearch,PMID: 32101754
9 Cy3-AffiniPure Donkey Anti-Rabbit IgG(H+L) Cat# 711-165-152, RRID: AB_2307443 Jackson ImmunoResearch,PMID: 36778241

Animals and other research organisms

Policy information about studies involving animals; ARRIVE guidelines recommended for reporting animal research, and Sex and Gender in Research

Laboratory animals: 7-8 weeks of age male mice C57BL/6J wild-type mice were purchased from the Institute of Experimental Animals of Sun Yat-sen University. The GAD2-Cre mice and CaMKIIα-Cre mice were graciously provided by the colleagues in Southern Medical University.

Wild animals: No wild animals were used in this study.

Reporting on sex: Only male mice were used to minimize variability related to the estrous cycle, thereby improving the robustness and interpretability of the results; this has also been clearly stated in the Abstract and Methods sections.

Field-collected samples: This study did not involve field-collected samples

Ethics oversight: All experiments were conducted according to protocols approved by The Sun Yat-sen University Institutional Animal Care and Use Committee (approval number SYSU-IACUC-2024-000851).

Note that full information on the approval of the study protocol must also be provided in the manuscript.

Plants

Seed stocks	*Report on the source of all seed stocks or other plant material used. If applicable, state the seed stock centre and catalogue number. If plant specimens were collected from the field, describe the collection location, date and sampling procedures.*
Novel plant genotypes	*Describe the methods by which all novel plant genotypes were produced. This includes those generated by transgenic approaches, gene editing, chemical/radiation-based mutagenesis and hybridization. For transgenic lines, describe the transformation method, the number of independent lines analyzed and the generation upon which experiments were performed. For gene-edited lines, describe the editor used, the endogenous sequence targeted for editing, the targeting guide RNA sequence (if applicable) and how the editor was applied.*
Authentication	*Describe any authentication procedures for each seed stock used or novel genotype generated. Describe any experiments used to assess the effect of a mutation and, where applicable, how potential secondary effects (e.g. second site T-DNA insertions, mosiacism, off-target gene editing) were examined.*
