# Supplementary material for: Lateral septum GABAergic neurons mediate the effects of dexmedetomidine on allodynia and sleep in a male mouse model of neuropathic pain
Source: Nat Commun. 2026 Jun 19;17:7725. doi: 10.1038/s41467-026-74217-3 (PMC13434266; doi:10.1038/s41467-026-74217-3)
Supplement: Supplementary file 3 [file 41467_2026_74217_MOESM3_ESM.pdf]

nature portfolio

Peer Review File

Lateral septum GABAergic neurons mediate the effects of dexmedetomidine on allodynia and sleep in a male mouse model of neuropathic pain

Corresponding Author: Professor Xia Feng

This file contains all reviewer reports in order by version, followed by all author rebuttals in order by version.

Version 0:

Reviewer comments:

Reviewer #1

(Remarks to the Author)
This study by Fan et al. reveals a shared neural substrate underlying the comorbidity of chronic pain and sleep disorders, and identifies Dexmedetomidine as a potential therapeutic agent that acts via selective modulation of LSD-GABAergic circuits. The findings are of high significance and broad relevance. The experimental design is comprehensive, featuring exemplary use of state-of-the-art molecular and genetic tools. Moreover, these approaches yielded a substantial body of high-quality data that robustly supports the authors' conclusions. The manuscript is, for the most part, clearly written. I only have minor suggestions aimed at further improving the clarity of certain data presentations.

Regarding Figure 1:
1. Please specify the phase (light or dark) during which the EEG/EMG recordings were conducted. Were any comparisons made between phases to account for potential circadian influences?
2. Does Dexmedetomidine administration alter sleep-wake patterns or paw withdrawal thresholds in sham-operated control mice?
3. In panels 1F and 1G, the Y-axis of the EEG spectrograms lacks a unit. Additionally, the top scale bar is labeled as "Power"—it should more precisely indicate "EEG Power" or similar, and the Y-axis should be clearly labeled as "EEG Frequency".
4. There is a spelling error ("stag") in the Y-axis label.
5. The legends for panels 1H and 1I are incorrect. The current label for 1H corresponds to NREM sleep, while 1G corresponds to REM sleep.
6. Please clarify when the paw withdrawal threshold test was conducted following dexmedetomidine administration. Were the mice in a sleep state during this assessment?
7. Please include a detailed description of the paw withdrawal threshold test in the Methods section.
Regarding Figure 2:
8. For the calcium imaging in panels 2C and 2D, please specify the experimental timeline and the behavioral state (e.g., wakefulness, NREM, or REM sleep) during which the recordings were analyzed.
9. In panel 2H, please clearly label the specific sleep-wake transitions being analyzed (e.g., Wake-to-NREM, NREM-to-Wake).
10. Statistical analysis for the calcium signal traces in panel 2H should be provided to support the reported dynamics.
11. In the right panel of Fig. 2H, the example trace of the sleep-to-wake transition appears suboptimal, as the wakefulness period is relatively brief.
12. Please indicate the concentration of CNO used in these experiments.
13. For the chemogenetic manipulations, inclusion of an mCherry+CNO control group would be necessary to confirm the specificity of the observed effects.

Regarding Figure 3:
14. The figure legends and corresponding panels appear to be mismatched. There are two panels labeled "J," which should be corrected for consistency.
15. In Supplementary Fig. 3, please clarify whether optogenetic activation of LSD GABAergic neurons alters the amount of NREM sleep, REM sleep, or wakefulness.

Regarding Figure 4:
16. There is a consistent misalignment between the figure legend and the corresponding panels. Specifically, the descriptions for panels G, F, H, I, and J do not match their intended figures. Similar discrepancies are evident for panels M-P and R-U.
17. The authors administered dexmedetomidine via intra-LSD injection before performing patch-clamp recordings in LSD slices. Would similar electrophysiological effects be observed if dexmedetomidine were applied directly to the ACSF?
18. The authors used AAVs to knock down the α2A adrenergic receptor. Please provide data demonstrating the efficiency of this knockdown.

Regarding Figure 5:
19. In Fig. 5C and 5D, the anatomical positions of the LSD and LPO appear to be misplaced. Please verify and correct their locations.
20. αCaMKII is not a reliable marker for glutamatergic neurons in subcortical regions. Please confirm that the αCaMKII-expressing neurons in the LPO are indeed glutamatergic.

Regarding Figure 7:
21. Lines 452–470 contain an incorrect figure citation: the reference refers to Fig. 7 rather than Fig. 6.
22. For Figs. 7G and 7H, only 4 mice were included per group. Please increase the sample size to enhance statistical power

Reviewer #2

(Remarks to the Author)
The authors have submitted a manuscript that proposes the role of LSDGABA neurons in pain and sleep disorder comorbidity. Overall, the experiments in this study are carefully designed and executed, and the results generally support the authors' conclusions. Several aspects of the evidence are not convincing, and additional experiments are required to solidify the claims.

Major comments:
1: In Figure 1G–I, the results show that 25 μg/kg DEX improve sleep, including non-rapid eye movement (NREM) sleep and rapid eye movement (REM) sleep. However, the total duration of the three brain states adds up to less than 480 minutes, which appear to be mislabeled.

2: In the figure 2A, 28 days after spared nerve injury (SNI), many brain region including the anterior cingulate cortex (ACC), lateral hypothalamus (LH), paraventricular thalamic nucleus (PVT), periaqueductal gray (PAG), posterior hypothalamus (PH), and dorsal lateral septum (LSD) show c-fos increase. The authors' justification for focusing on the LSD appears insufficient. They primarily cite a reference implicating the LSD in sleep-wake regulation. However, since several of the identified regions (e.g., the PVT (Sciences, 2018)) are classically known to regulate sleep-wake cycles, this alone is not a compelling rationale. To precisely identify the key brain regions involved, the study should have included a comparison across all four experimental groups: sham, SNI, SNI+saline, and SNI+Dex.

3. In the figure2 D, the results indicate that the AUC in the SNI group is significantly larger than that in the saline group. However, the methodology raises concerns. According to the representative traces, the authors quantified the AUC over a brief, unstimulated baseline period of approximately 200-300 seconds. Given that SNI is a chronic pain model, sustained neuronal activation in the target brain region is expected. Therefore, it is puzzling why the analysis focused on such a short, arbitrary time window without any applied stimulus.

4: In the figure 2G, only a minor difference was observed between the SNI and sham groups, which lacked statistical support and is thus not considered reliable.

5: In the figure2 H, a poor correspondence between LSD calcium activity and EEG frequency profiles during sleep-wake transitions.

6: DEX as a nonopioid analgesics previvors study had found that LC was main target brain region. Although intraperitoneal dexmedetomidine significantly suppressed the SNI-induced elevation of calcium signals in LSDGABA neurons (Fig. 4A-B), the effect size was marginal (0.12%). Notably, the observed reduction was even less pronounced than that in the saline-treated control group.

7: The calcium signaling findings are not compelling, as exemplified by Figure 6N. The reported change of 0.004% (calculated per methods as $(F - F_0)/F_0$) lacks both a control comparison and statistical testing, rendering its biological interpretation unreliable.

8: A critical control is missing across all chemogenetics experiments: a DIO-mCherry group. This group is essential to account for potential non-specific effects of the viral injection and transgene expression, thereby ensuring that the observed effects are specifically due to the activation of the designated receptor (hM3Dq/hM4Di).

Minor comments:

1: All color bars in the manuscript should be accompanied by their corresponding numerical values to allow for accurate data interpretation.

2: The use of arbitrary and inconsistent data ranges undermines the comparability of the results. A prominent example is in Figure 3 O, where the EMG activity for the two groups is displayed on vastly different scales (±200 vs. ±5000), Figure 4F (±200 vs. ±2000), making a direct visual comparison invalid.

Reviewer #3

(Remarks to the Author)
Chronic pain and sleep disorders frequently co-occur and exacerbate each other, yet the neural circuit mechanisms underlying this comorbidity remain poorly understood. This work reports that hyperactivity of GABAergic neurons in the LSD drives both chronic pain and sleep disorders through projections to glutamatergic neurons and GABAergic neurons in the LPO respectively. Moreover, systemic or local application of DEX alleviated comorbid pain and sleep disorders through blunting the LSD GABAergic neuronal hyperactivity in an α2A-receptor dependent manner. These findings reveal a shared neural substrate for chronic pain and sleep disorders and establish DEX as a potential treatment for this comorbidity through tuning the LSD-driven circuit activity.

1. Fig. 1I. It is puzzling that DEX has no effect on the REM phase, although the duration of REM stage was also significantly decreased by the SNI (Fig. 1E).

2. I am wondering why the authors chose to record the EEG/EMG for 6 h (Fig. 1F). What is the rationale for selecting 6 h instead of 12 h or 24 h? I think it better to analyze the time spent in wake, NREM and REM over a 12 h period.

3. Fig. 3H and Fig. S3A. Please provide the electrophysiological validation of the efficacy of neuronal inhibition by NpHR vs. activation by ChR2. Please revise the Fig. 3 and Fig. 4 legends. Several mistakes occurred.

4. Please draw a schematic timeline for behavioral experiments shown in Fig. 3, Fig. 6, Fig. 7 and Fig. 8.

5. Fig. S2A, the whole-brain c-Fos mapping data should be quantified to illustrate the relative differences of c-Fos+ cell numbers among each brain region. Similarly, please add the quantitative analysis of the whole-brain output tracing data shown in Fig. S4A.

6. Fig. 4A-B. It is necessary to further test whether DEX could alleviate the rise in Ca^{2+} signals when the SNI-treated animals received mechanical stimulus or experienced sleep-wake transitions (as shown in Fig. 2G and H).

7. Fig. 4K should provide a quantification of the relative expression abundance of α2A in LSD GABAergic neurons. The authors should also verify the knockdown efficiency of AAV-Vgat-α2A-shRNA in Fig. 4Q.

8. Fig. 6 and Fig. 7. It is highly recommended that the authors performed more whole-cell patch-clamp recordings to evaluate any changes in synaptic plasticity (PPR, I-O curve of light-evoked IPSC amplitude) of the LSD→LPOGlu and LSD→LPOGABA projection in the comorbidity mice.

9. Fig. 8B and C. The legend said "The c-Fos expression of LSD and LPO after intraperitoneal injection of dexmedetomidine in SNI mice", but no any c-Fos data in the LSD region was shown. In addition, compared with the SNI only group, the SNI+DEX group showed an elevated c-Fos expression, indicating the possibility that DEX blocked the LSD GABAergic neurons through α2A receptor, resulting in a disinhibition of the downstream LPO neurons. However, Fig. 8A shows the expression of α2A receptor in the LPO neurons. Then how to exclude the possibility that DEX acts directly on the α2A in LPO neurons to inhibit the LPO neuronal activity instead of indirect disinhibition?

10. The experimental design of Fig. 8F-M seems wrong. Since the LPO GABA or Glu neurons innervated by LSD have already been strongly inhibited by chemogenetic manipulations, it is reasonably deduced that intra-LSD injection of DEX could not further activate the LPO neurons to reverse CNO-induced mechanical allodynia and sleep deficits.
In contrast, to demonstrate that DEX alleviates pain hypersensitivity and comorbid sleep disorder via the LSD→LPO circuit, the authors should activate the LPOGlu-projecting LSD neurons or activate the LPOGABA-projecting LSD neurons, then check whether DEX still attenuates mechanical allodynia and sleep deficits. Or else, if DEX indeed exerts the beneficial effects through activating the LPO neurons (Fig. 8E and K), then optogenetic or chemogenetic inhibition of the LPO glu or GABA neurons should also block the analgesic or sleep-improving effect of DEX.

Version 1:

Reviewer comments:

Reviewer #1

(Remarks to the Author)
Thank you to the authors for thoroughly addressing the reviewers' questions and incorporating the suggested experiments. These revisions have strengthened both the manuscript and the conclusions drawn from the data. I recommend the current manuscript for publication.

Reviewer #2

(Remarks to the Author)
The authors have done a remarkable job to address all of my concerns about the original manuscript. The manuscript reports an impressive set of well conducted experiments supporting their claim.

Reviewer #3

(Remarks to the Author)
The resubmission from Fan et al. includes numerous additional experiments which significantly strengthen the manuscript and satisfy the reviewer's initial concerns. Some additional comments are required to be addressed before considering publication of the manuscript.

(1) Supplementary Fig. 5A. CTB should be changed into "LSDGABA mCherry+ fibers". Panel (R) should be moved to Panel (B). Panel (B) should be changed to (C), and so on.

(2) I highly recommend integrating Comment Fig. 7 (confirmation of the knock down efficacy) into the manuscript.

(3) Similarly, Comment Fig. 17 should also be included into the main manuscript.

(4) I still think that the experimental design of Fig. 8F–I and Fig. 8L–Q is problematic. The authors said that intra-LSD DEX was able to counteract CNO-evoked mechanical hypersensitivity and/or sleep disruption. But how to explain the results? How does DEX achieve this effect under the condition of LPO neuronal inhibition by CNO? In other words, what are the mechanisms underlying this reversal effect of DEX?

Instead, I would suggest that the authors delete these panels (Fig. 8F–I and Fig. 8L–Q) and replace them with the Comment Fig. 21, which straightforwardly demonstrates that CNO-mediated LPO neuronal inhibition significantly attenuates the analgesic and sleep-promoting effects of DEX. However, Comment Fig. 21 B and C should clearly show the detailed chemogenetic inhibition target (LPO GABA or LPO Glu) for each behavioral result. Moreover, for Comment Fig. 21A, it is better to perform CNO/saline i.p. administration before (not after) the intra-LSD DEX/saline microinjection?

Version 2:

Reviewer comments:

Reviewer #3

(Remarks to the Author)
The authors have further revised the manuscript and now addressed all of my original concerns. I have no more comments.

Open Access This Peer Review File is licensed under a Creative Commons Attribution 4.0 International License, which permits use, sharing, adaptation, distribution and reproduction in any medium or format, as long as you give appropriate credit to the original author(s) and the source, provide a link to the Creative Commons license, and indicate if changes were made.
In cases where reviewers are anonymous, credit should be given to 'Anonymous Referee' and the source.
The images or other third party material in this Peer Review File are included in the article's Creative Commons license, unless indicated otherwise in a credit line to the material. If material is not included in the article's Creative Commons license and your intended use is not permitted by statutory regulation or exceeds the permitted use, you will need to obtain permission directly from the copyright holder.
To view a copy of this license, visit https://creativecommons.org/licenses/by/4.0/

Dear Editor and Reviewers,

We thank you very much for the thorough and thoughtful evaluation of our manuscript. We greatly appreciate the reviewers' insightful comments and constructive suggestions, which have helped us to substantially improve the rigor, clarity, and overall quality of the work.

In response to these comments, we have carefully revised the manuscript and, where appropriate, performed additional analyses and experiments. We have addressed each comment in detail below in a point-by-point manner. All revisions have been clearly indicated in the revised manuscript.

Reviewer 1:

This study by Fan et al. reveals a shared neural substrate underlying the comorbidity of chronic pain and sleep disorders, and identifies Dexmedetomidine as a potential therapeutic agent that acts via selective modulation of LSD-GABAergic circuits. The findings are of high significance and broad relevance. The experimental design is comprehensive, featuring exemplary use of state-of-the-art molecular and genetic tools. Moreover, these approaches yielded a substantial body of high-quality data that robustly supports the authors' conclusions. The manuscript is, for the most part, clearly written. I only have minor suggestions aimed at further improving the clarity of certain data presentations.

Regarding Figure 1:

Comment 1: Please specify the phase (light or dark) during which the EEG/EMG recordings were conducted. Were any comparisons made between phases to account for potential circadian influences?

Responses: Thank you for pointing out the important issue. In normal nocturnal mice, sleep–wake behavior and EEG/EMG activity exhibit robust circadian modulation, with consolidated sleep predominating during the light phase and increased wakefulness during the dark phase[1-2]. Accordingly, all EEG/EMG recordings in the present study were conducted during the light phase from 09:00 to 15:00, corresponding to ZT2–ZT8 under a 12 h light/12 h dark cycle (lights on at 07:00). This time window encompasses

the mid-light phase, during which sleep architecture and EEG spectral characteristics are relatively stable and spontaneous locomotor activity is minimal. Such timing is commonly used in mouse EEG/EMG studies to reduce circadian-related variability[3-4]. We did not perform direct comparisons between light and dark phases in this study. However, by restricting all recordings to a fixed circadian window, we aimed to control for potential circadian confounding effects across experimental groups. We have now clarified the timing of EEG/EMG data acquisition in the Electroencephalogram/Electromyogram (EEG/EMG) Recording section of the Methods.

Comment 2: Does Dexmedetomidine administration alter sleep-wake patterns or paw withdrawal thresholds in sham-operated control mice?

Responses: We thank the reviewer for this important question. To determine whether dexmedetomidine alters baseline sleep–wake patterns or nociceptive thresholds in the absence of pathology, we examined its effects in sham-operated control mice. Compared with saline-treated sham controls, dexmedetomidine did not significantly change the total duration of wakefulness, NREM sleep, or REM sleep (Comment Fig. 1A-D, $P > 0.05$). In addition, paw withdrawal thresholds to mechanical stimulation were not significantly different between dexmedetomidine- and saline-treated sham mice (Comment Fig. 1E, $P > 0.05$).

These results indicate that dexmedetomidine does not induce detectable changes in baseline sleep architecture or mechanical pain sensitivity in sham-operated control mice. We have incorporated these sham-control results into the revised manuscript, and the data are now shown in Supplementary Fig. 2.

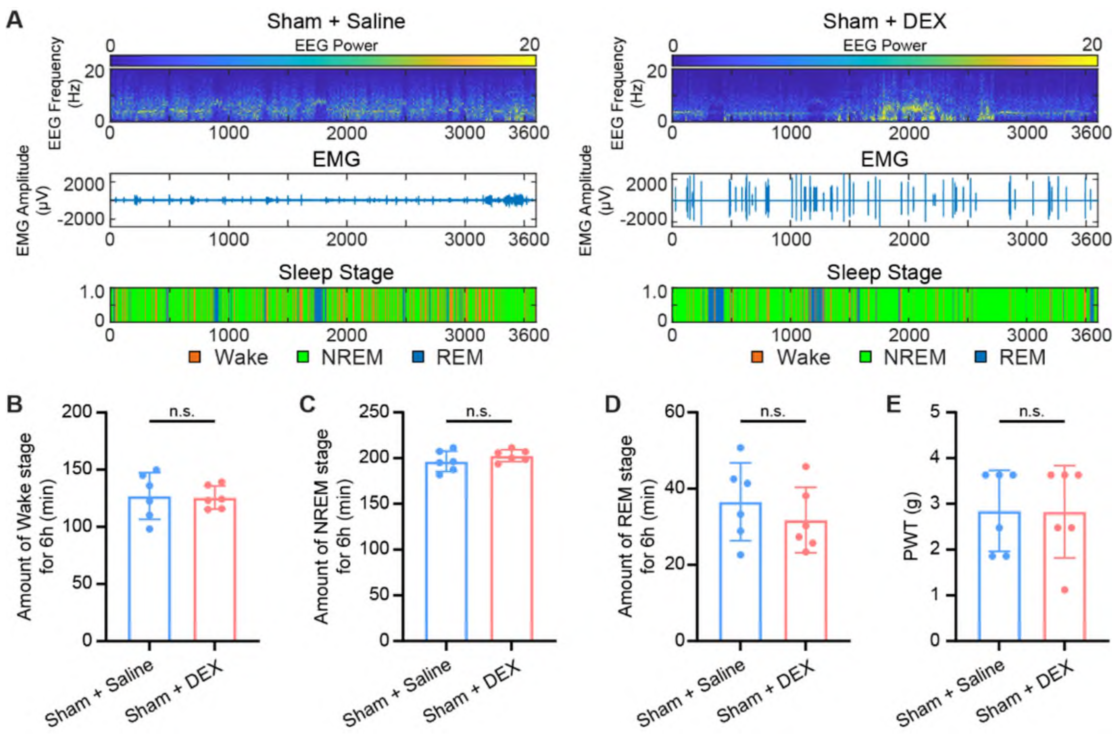

Comment Fig. 1 Dexmedetomidine does not alter sleep–wake architecture or mechanical pain sensitivity in sham-operated mice.

Comment 3. In panels 1F and 1G, the Y-axis of the EEG spectrograms lacks a unit. Additionally, the top scale bar is labeled as "Power"—it should more precisely indicate "EEG Power" or similar, and the Y-axis should be clearly labeled as "EEG Frequency".

Responses: We sincerely appreciate the reviewer's comments. In the revised manuscript, the original Fig. 1G has been renumbered as Fig. 1J. Accordingly, in Fig. 1F and Fig. 1J, as well as in all subsequent EEG/EMG figures throughout the manuscript, we have explicitly added the y-axis units (Hz for EEG frequency and μV for EMG amplitude), and the top scale has been consistently labeled as "EEG power".

Comment 4. There is a spelling error ("stag") in the Y-axis label.

Responses: We thank the reviewer for pointing out this spelling error ("stag"). The Y-axis label has now been corrected.

Comment 5. The legends for panels 1H and 1I are incorrect. The current label for 1H corresponds to NREM sleep, while 1G corresponds to REM sleep.

Responses: We sincerely appreciate the reviewer's comments. The legends for panels 1H and 1I have been corrected to accurately correspond to NREM and REM sleep, respectively.

Comment 6. Please clarify when the paw withdrawal threshold test was conducted following dexmedetomidine administration. Were the mice in a sleep state during this assessment?

Responses: We thank the reviewer for this important question. The paw withdrawal threshold (PWT) test was conducted 1 h after dexmedetomidine administration. Although dexmedetomidine can increase sleep time within this window, PWT measurements were performed only when mice were awake and behaviorally responsive; no testing was conducted during NREM or REM sleep.

The dexmedetomidine dose used in this study (25 μg/kg) is not expected to induce a sedative state or impair motor performance (Fig. 1K, Supplementary Fig. 1H-O). Our behavioral assessment strategy is consistent with previous studies on sleep–pain comorbidity, in which pain-like behaviors were assessed using mechanical sensitivity tests in awake animals while sleep states were evaluated separately[5-6]. In addition, prior preclinical studies of systemic dexmedetomidine analgesia have assessed mechanical allodynia using dosing ranges and post-administration time points similar to those used here [7-8].

The timing and behavioral criteria for PWT testing have now been clarified in the Methods section of the revised manuscript.

Comment 7. Please include a detailed description of the paw withdrawal threshold test in the Methods section.

Responses: We thank the reviewer for this suggestion. We have revised the Methods section (Behavioral Testing) to provide a more detailed description of the paw withdrawal threshold (PWT) test.

Regarding Figure 2:

Comment 8. For the calcium imaging in panels 2C and 2D, please specify the experimental timeline and the behavioral state (e.g., wakefulness, NREM, or REM sleep) during which the recordings were analyzed.

Responses: We are grateful for pointing out the important issues. The calcium imaging data in Fig. 2C–D reflect spontaneous (baseline) fiber photometry Ca^{2+} signals from LSD^{GABA} neurons that were recorded and analyzed during the wakefulness state (EEG/EMG-defined wake epochs), with NREM and REM epochs excluded. We chose the wakefulness state to provide a standardized condition for group comparisons and to avoid potential confounding effects from sleep-stage–dependent fluctuations in calcium activity, consistent with prior awake-state fiber photometry studies[9-10]. Given the chronic pain–sleep disorder comorbidity model, focusing on spontaneous activity during awake epochs—particularly within the insomnia-associated period—is most relevant for capturing the disease-related neural state. This clarification has been added to the revised manuscript in the Fig. 2D legend.

Comment 9. In panel 2H, please clearly label the specific sleep-wake transitions being analyzed (e.g., Wake-to-NREM, NREM-to-Wake).

Responses: We thank the reviewer for this comment. In response, we have revised Fig. 2H to clearly label the specific sleep–wake transitions being analyzed as Wake-to-NREM and NREM-to-Wake, consistent with prior studies that annotate sleep–wake transitions in this manner [11-12]. These labels have been added to the revised figure, now presented as Fig. 2I in the updated manuscript.

Comment 10. Statistical analysis for the calcium signal traces in panel 2H should be provided to support the reported dynamics.

Responses: We appreciate the reviewer's thoughtful comment. To provide statistical support for the calcium-signal dynamics, we have added a quantitative analysis of the photometry traces by calculating the area under the curve (AUC) within defined time windows around the labeled sleep–wake transitions. These results are now presented in the revised figure, which has been renumbered from Fig. 2H to Fig. 2I, and the

corresponding statistical comparisons are included in Supplementary Fig. 3F, G and the figure legend.

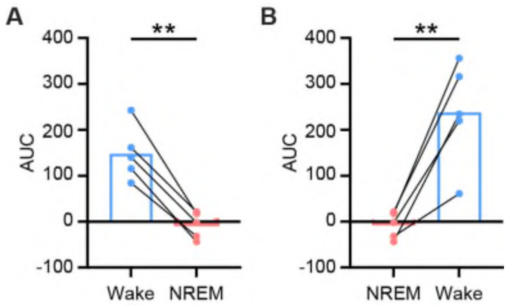

Comment Fig. 2 Wake–NREM and NREM–Wake transition–associated Ca^{2+} AUC in LSD^{GABA} neurons

Comment 11. In the right panel of Fig. 2H, the example trace of the sleep-to-wake transition appears suboptimal, as the wakefulness period is relatively brief.

Responses: We thank the reviewer for pointing this out. We agree that the correspondence may appear weak in the originally shown example. We have therefore replaced the EEG/EMG example with a more representative trace to better match the transition-aligned average Ca^{2+} dynamics. Importantly, our conclusions are based on the transition-aligned population averages across animals and events, and the results text and figure legend have been updated accordingly in the revised manuscript (the original Fig. 2H is now presented as Fig. 2I).

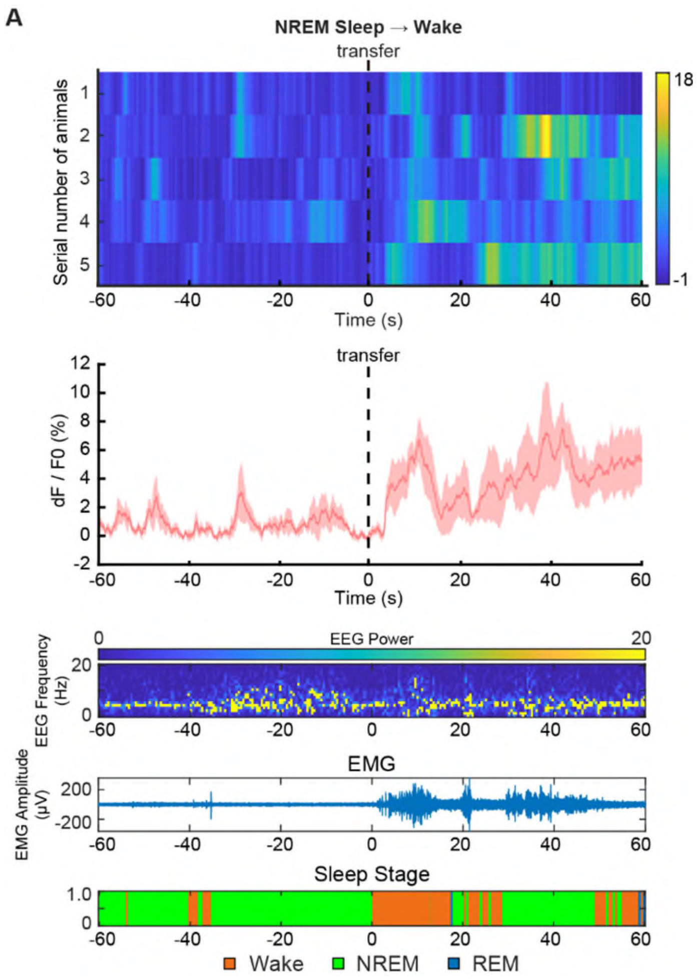

Comment Fig. 3 Ca^{2+} activity of LSD^{GABA} neurons across the NREM–Wake transition

Comment 12. Please indicate the concentration of CNO used in these experiments.

Responses: We thank the reviewer for raising this point. The CNO dose used in the in vivo chemogenetic experiments was 1 mg/kg (i.p.), which is consistent with commonly used dosing in prior DREADD studies[13-14]. In addition, for ex vivo whole-cell patch-clamp validation, CNO was applied at 5 μM (bath application) to confirm inhibition of hM4Di-expressing neurons, consistent with prior electrophysiological DREADD validation protocols [15-16]. Although the in vivo CNO dose was already provided in the chemogenetic manipulation section of the Methods, to make this information more visible we have now also added the in vivo CNO dose (and the ex vivo CNO concentration where applicable) to the figure legend(s) and the corresponding Results descriptions in the revised manuscript.

Comment 13. For the chemogenetic manipulations, inclusion of an mCherry+CNO control group would be necessary to confirm the specificity of the observed effects.

Responses: We thank the reviewer for this important suggestion. To exclude potential off-target effects of CNO and confirm specificity, we added an mCherry control cohort receiving CNO (with the corresponding saline control) and analyzed Wake, NREM, REM and PWT using two-way ANOVA (virus × treatment) followed by Sidak's multiple-comparisons test. In mCherry controls, CNO did not affect any readout (mCherry saline vs CNO: M4 Wake $p=0.9631$, NREM $p=0.9330$, REM $p=0.9605$, PWT $p=0.9998$; M3 Wake $p=0.8998$, NREM $p=0.8945$, REM $p=0.7889$, PWT $p=0.9722$). By contrast, under CNO conditions, DREADD-expressing mice differed significantly from mCherry controls for Wake/NREM and PWT (M4: Wake $p=0.0002$, NREM $p=0.0002$, PWT $p<0.0001$; M3: Wake $p=0.0099$, NREM $p=0.0035$, PWT $p<0.0001$), supporting that the observed phenotypes are DREADD-dependent rather than attributable to CNO alone. The updated Fig. 3 incorporating these control data has been included in the revised manuscript (Fig. 3D, F–H; Supplementary Fig. 4L–P).

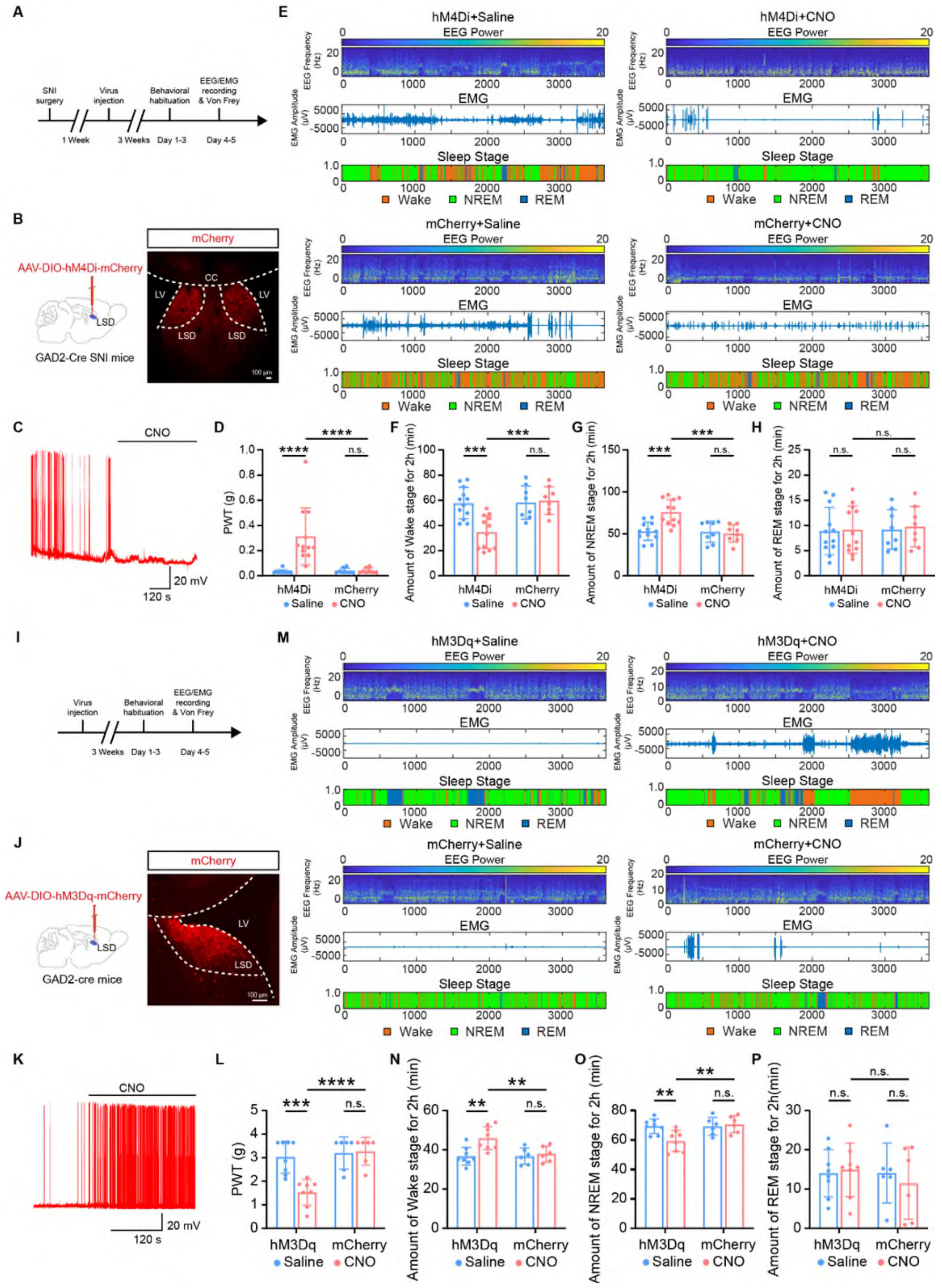

Comment Fig. 4 Chemogenetic manipulation of LSDGABA neurons modulates the comorbidity of chronic pain and sleep disorders

Regarding Figure 3:

Comment 14. The figure legends and corresponding panels appear to be mismatched.

There are two panels labeled “J,” which should be corrected for consistency.

Responses: We appreciate the reviewer’s comment. We have corrected the duplicated panel label (two panels labeled “J”) and updated the Fig. 3 panel lettering and corresponding legend accordingly in the revised manuscript.

Comment 15. In Supplementary Fig. 3, please clarify whether optogenetic activation of LSD GABAergic neurons alters the amount of NREM sleep, REM sleep, or wakefulness.

Responses: We thank the reviewer for raising this important point. In the revised Supplementary Fig. 4 (renumbered from the original Supplementary Fig. 3), we now quantify the amount of time spent in Wake, NREM, and REM during optogenetic activation of LSD GABAergic neurons based on EEG/EMG scoring. This quantification approach follows previously established methods in the sleep/optogenetics literature[17-18]. Specifically, sleep–wake states were scored in 4-s epochs and expressed as the percentage of time spent in each state across consecutive light-off (−120–0 s), light-on (0–30 s), and light-off (30–150 s) windows aligned to laser onset (new Supplementary Fig. 4E–G). These analyses show that optogenetic activation increases wakefulness and decreases NREM and REM sleep during the light-on period. This clarification has been incorporated into the revised Results text, figure legend, and the Methods section (Optogenetic manipulation).

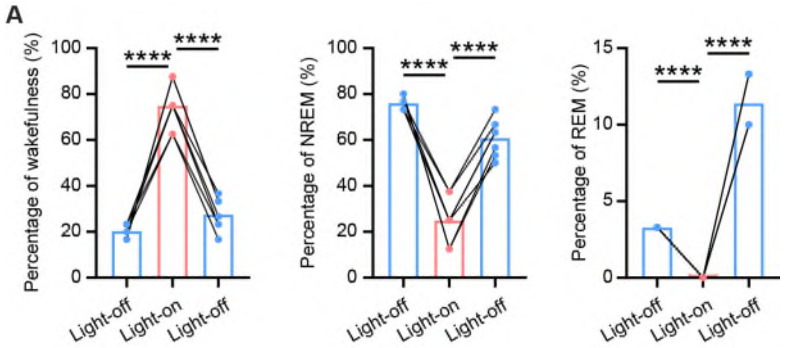

Comment Fig. 5 Optogenetic activation of LSD^{GABA} neurons increases wakefulness and suppresses NREM/REM sleep

Regarding Figure 4:

Comment 16. There is a consistent misalignment between the figure legend and the corresponding panels. Specifically, the descriptions for panels G, F, H, I, and J do not match their intended figures. Similar discrepancies are evident for panels M-P and R-U.

Responses: We apologize for the mismatches between the Fig. 4 legend and the corresponding panels. We have reassigned the descriptions to the correct subpanels, particularly for F–J, M–P, and R–U, and updated the revised manuscript accordingly.

Comment 17. The authors administered dexmedetomidine via intra-LSD injection before performing patch-clamp recordings in LSD slices. Would similar electrophysiological effects be observed if dexmedetomidine were applied directly to the ACSF?

Responses: We thank the reviewer for raising this point. To test whether dexmedetomidine exerts a direct effect on LSD neurons, we performed additional ex vivo whole-cell recordings in which dexmedetomidine was applied directly in the ACSF (bath application; ACSF, n = 15 cells; DEX, n = 14 cells). Consistent with the intra-LSD microinjection paradigm, acute dexmedetomidine exposure in slices similarly reduced neuronal excitability, reflected by a downward shift of the input–output firing curve (two-way ANOVA: $F(1,27) = 9.811$, $p = 0.0041$; Comment Fig.6). These effects were consistent with those obtained following intra-LSD dexmedetomidine administration (Fig. 4C; n = 17 cells; two-way ANOVA: $F(1,32) = 4.779$, $p = 0.0362$).

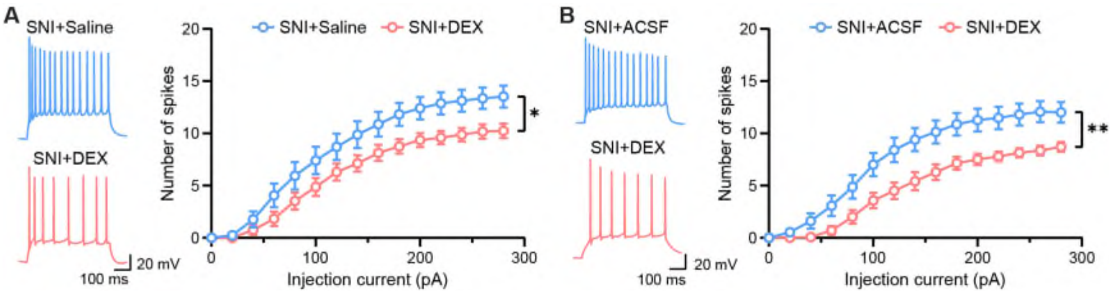

Comment Fig. 6 Bath-applied dexmedetomidine directly suppresses LSD neuronal excitability ex vivo

Comment 18. The authors used AAVs to knock down the α2A adrenergic receptor. Please provide data demonstrating the efficiency of this knockdown.

Responses: We thank the reviewer for this request. To validate the efficiency of the AAV-VGAT-α2A-shRNA knockdown, we quantified α2A adrenergic receptor (Adra2a) mRNA in LSD tissue from SNI mice injected with AAV-VGAT-α2A-shRNA or AAV-VGAT-α2A-scramble. This approach is consistent with prior in vivo AAV-shRNA studies that validate knockdown by RT-qPCR from AAV-infected brain tissue punches[19]. Our results showed a significant reduction of Adra2a transcript levels in the shRNA group compared with scramble controls, confirming effective knockdown (Comment Fig.7).

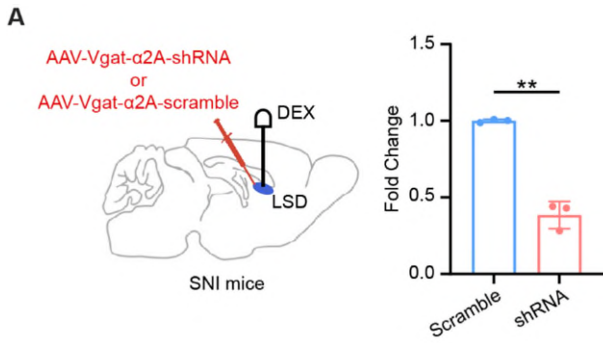

Comment Fig. 7 RT–qPCR confirms efficient AAV-VGAT-α2A-shRNA knockdown of Adra2a in the LSD

Regarding Figure 5:

Comment 19. In Fig. 5C and 5D, the anatomical positions of the LSD and LPO appear to be misplaced. Please verify and correct their locations.

Responses: We thank the reviewer for pointing this out. We confirmed that the anatomical labels for the LSD and LPO were incorrectly labeled in the original Fig. 5C and 5D. This labeling error has been corrected, and the two panels have been combined and are now presented together as Fig. 5C in the revised manuscript.

Comment 20. αCaMKII is not a reliable marker for glutamatergic neurons in subcortical regions. Please confirm that the αCaMKII-expressing neurons in the LPO are indeed glutamatergic.

Responses: We appreciate the reviewer's thoughtful comment. We agree that αCaMKII alone is not a definitive or exclusive marker of glutamatergic neurons in subcortical regions, and therefore it should not be used as the sole criterion for transmitter identity. Nonetheless, CaMKIIα expression is present in the hypothalamus, including the LPO region, as supported by the Allen Mouse Brain Atlas[20]. Moreover, CaMKIIα promoter–driven AAV expression has been successfully used in hypothalamic circuits (e.g., lateral hypothalamus) to preferentially target excitatory neuronal populations[21]. To directly address the reviewer's concern, we performed additional validation experiments and confirmed that CaMKIIα-expressing LPO neurons are glutamatergic by co-immunostaining with the canonical glutamatergic marker Vglut2, which showed robust colocalization (Comment Fig. 8).

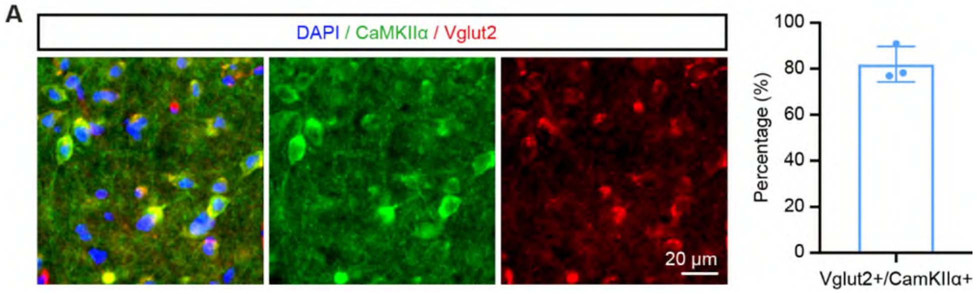

Comment Fig. 8 Vglut2 colocalization validates CaMKIIα-targeted LPO neurons as glutamatergic

Regarding Figure 7:

Comment 21. Lines 452–470 contain an incorrect figure citation: the reference refers to Fig. 7 rather than Fig. 6.

Responses: We thank the reviewer for pointing out this incorrect figure citation. We have corrected the figure reference from Fig. 6 to Fig. 7 in the Results section of the revised manuscript.

Comment 22. For Figs. 7G and 7H, only 4 mice were included per group. Please increase the sample size to enhance statistical power

Responses: We appreciate the reviewer's concern regarding statistical power. To address this, we expanded the cohort from 4 to 8 mice per group, re-analyzed the data,

and updated the corresponding figure panels. With the increased sample size, the effects remained consistent: CNO reduced Wake time (unpaired t-test, t(14)=8.014, p<0.0001) and increased NREM time (t(14)=8.234, p<0.0001), while REM time (t(14)=0.4017, p=0.6940) and PWT (t(14)=0.6788, p=0.5084) were unchanged. The revised panels are now presented as Fig. 7G–L (expanded from the original Figs. 7G and 7H).

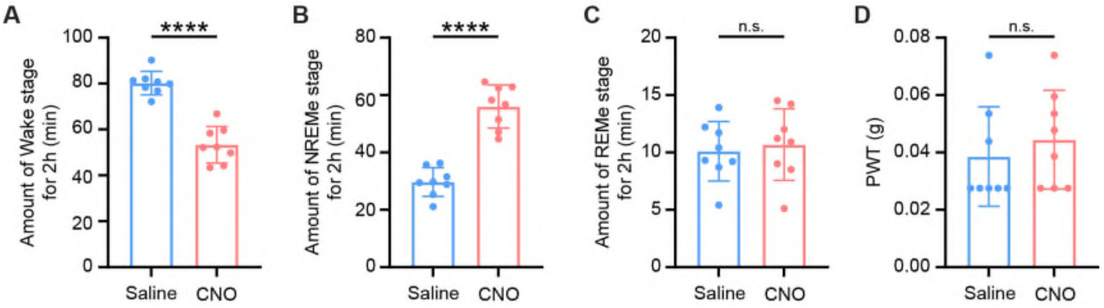

Comment Fig. 9 Chemogenetic activation of LPO GABA neurons promotes sleep without altering pain sensitivity

Reviewer 2:

The authors have submitted a manuscript that proposes the role of LSDGABA neurons in pain and sleep disorder comorbidity. Overall, the experiments in this study are carefully designed and executed, and the results generally support the authors' conclusions. Several aspects of the evidence are not convincing, and additional experiments are required to solidify the claims.

Major comments:

Comment 1: In Figure 1G–I, the results show that 25 µg/kg DEX improve sleep, including non-rapid eye movement (NREM) sleep and rapid eye movement (REM) sleep. However, the total duration of the three brain states adds up to less than 480 minutes, which appear to be mislabeled.

Responses: We thank the reviewer for pointing this out. We confirm that the EEG/EMG recording and sleep staging in this experiment were performed over 6 h (360 min). Therefore, the total time spent in Wake/NREM/REM should sum to 360 min, rather than 480 min. We have clarified this 6-h recording window in the revised manuscript by explicitly stating it in the figure legend and the corresponding text for Fig. 1G–I, and

we also specified the recording duration in the Methods section ("Electroencephalogram/Electromyogram (EEG/EMG) Recording").

Comment 2: In the figure 2A, 28 days after spared nerve injury (SNI), many brain region including the anterior cingulate cortex (ACC), lateral hypothalamus (LH), paraventricular thalamic nucleus (PVT), periaqueductal gray (PAG), posterior hypothalamus (PH), and dorsal lateral septum (LSD) show c-fos increase. The authors' justification for focusing on the LSD appears insufficient. They primarily cite a reference implicating the LSD in sleep-wake regulation. However, since several of the identified regions (e.g., the PVT (Sciences, 2018)) are classically known to regulate sleep-wake cycles, this alone is not a compelling rationale. To precisely identify the key brain regions involved, the study should have included a comparison across all four experimental groups: sham, SNI, SNI+saline, and SNI+Dex.

Responses: We thank the reviewer for this thoughtful comment. To address the concern, we have now quantified c-Fos across all four experimental groups (Sham, SNI, SNI+Saline, and SNI+DEX) for each surveyed region and provided these data in Comment Fig. 10. Importantly, this four-group comparison provides an additional, data-driven rationale for focusing on the LSD: among the candidate regions, the LSD showed the most pronounced SNI-associated increase in c-Fos and the strongest suppression by DEX (SNI+DEX vs SNI+Saline), whereas several other regions that also exhibited elevated c-Fos after SNI (including classic sleep–wake-related nuclei such as the PVT) showed relatively limited or non-significant modulation by DEX in our dataset. Thus, our selection of the LSD was based not only on prior literature linking it to sleep–wake regulation, but also on its maximal sensitivity to both SNI and DEX in the same screening assay, consistent with the hypothesis that the LSD is a key node through which DEX impacts the pain–sleep comorbidity phenotype.

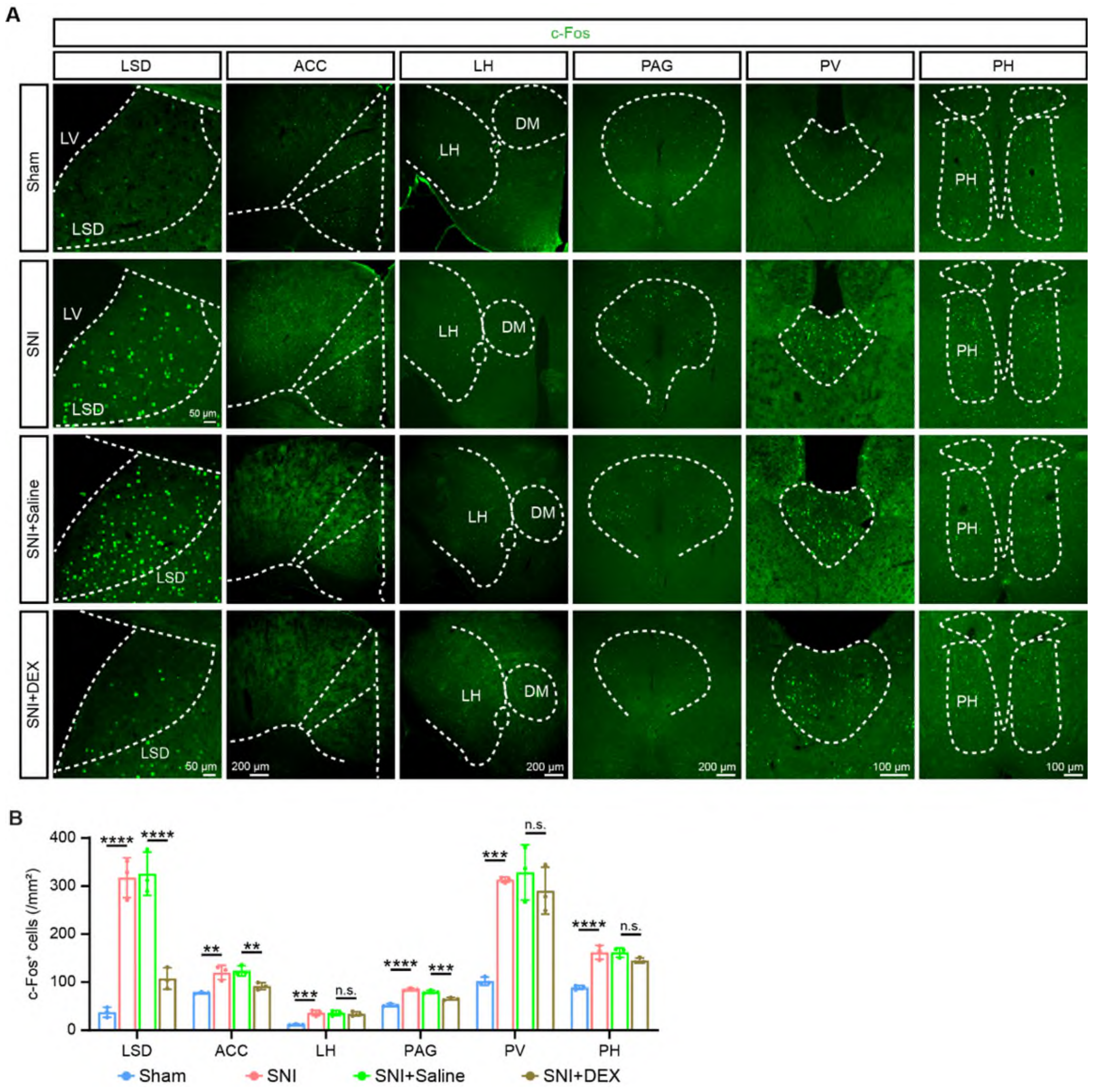

Comment Fig. 10 Quantification of c-Fos expression across Sham, SNI, SNI+Saline, and SNI+DEX groups

Comment 3. In the figure2 D, the results indicate that the AUC in the SNI group is significantly larger than that in the saline group. However, the methodology raises concerns. According to the representative traces, the authors quantified the AUC over a brief, unstimulated baseline period of approximately 200-300 seconds. Given that SNI is a chronic pain model, sustained neuronal activation in the target brain region is expected. Therefore, it is puzzling why the analysis focused on such a short, arbitrary time window without any applied stimulus.

Responses: We appreciate the reviewer’s thoughtful comment. In Fig. 2D, our goal was

to quantify spontaneous (baseline) Ca^{2+} activity of LSD^{GABA} neurons during EEG/EMG-defined stable wakefulness, rather than stimulus-evoked responses. Quantification of baseline calcium activity in vivo (resting-state, without external stimulation) is a commonly used approach to compare intrinsic activity differences across conditions[22-23].

Regarding the duration, we analyzed a continuous ~200–300 s segment because it allows selection of a state-stable wake epoch while minimizing contamination by brief micro-arousals or sleep transitions, and it reduces slow confounds such as photobleaching, baseline drift, and motion-related artifacts that can accumulate over longer recordings. Importantly, the same window length and selection criteria were applied across all animals and groups.

Comment 4: In the figure 2G, only a minor difference was observed between the SNI and sham groups, which lacked statistical support and is thus not considered reliable.

Responses: We thank the reviewer for this comment. To quantitatively evaluate the effect in Fig. 2G, we computed the AUC and analyzed the data using a two-way repeated-measures ANOVA (group × time; before vs after). This analysis revealed a significant main effect of time ($F(1,8)=18.67$, $P=0.0025$). Post hoc Šídák's multiple-comparisons tests further showed that AUC increased significantly from before to after in the SNI group (adjusted $P=0.0030$), whereas no significant change was detected in the sham group (adjusted $P=0.2343$). In addition, between-group comparisons indicated no baseline difference (adjusted $P=0.7316$), but a significant difference emerged after stimulation (adjusted $P=0.036$).

To further strengthen robustness, we repeated the experiment and increased the sham sample size (the original sham dataset included only four mice), and the overall trend remained consistent. In the replication experiment with an increased sham sample size, the AUC analysis revealed a significant group × time interaction ($F(1,14)=45.85$, $P<0.0001$) and a significant SNI–sham difference after stimulation (Šídák adjusted $P<0.0001$), with no difference before stimulation (adjusted $P=0.9752$). The results from the replication experiment have now been used to update Fig. 2G-H and the

corresponding figure legend in the revised manuscript.

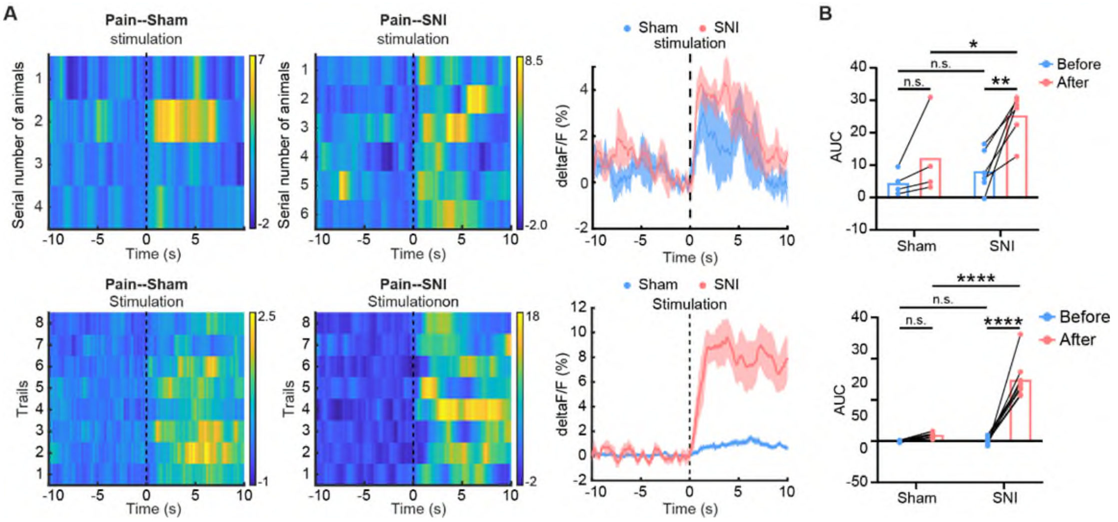

Comment Fig. 11 AUC analysis confirms pain stimulus–evoked Ca^{2+} activity in LSD^{GABA} neurons

Comment 5: In the figure2 H, a poor correspondence between LSD calcium activity and EEG frequency profiles during sleep-wake transitions.

Responses: We appreciate the reviewer's comment. We agree that the original EEG/EMG example may not have fully captured the transition-aligned Ca^{2+} dynamics. To address this, we have replaced the previous example with a more representative trace that better aligns with the average Ca^{2+} dynamics across transitions. Importantly, our conclusions rely on population averages aligned to the transitions across animals and events. As a result, both the results text and figure legend have been updated in the revised manuscript, with the original Fig. 2H now shown as Fig. 2I.

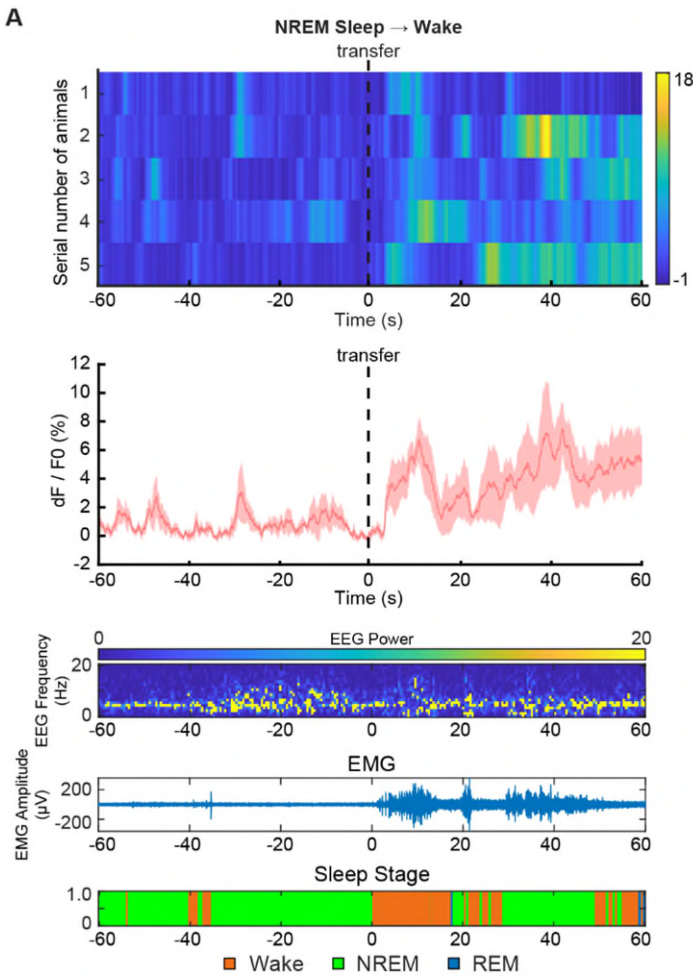

Comment Fig. 12 Ca^{2+} activity of LSD^{GABA} neurons across the NREM–Wake transition

Comment 6: DEX as a nonopioid analgesics previvors study had found that LC was main target brain region. Although intraperitoneal dexmedetomidine significantly suppressed the SNI-induced elevation of calcium signals in LSDGABA neurons (Fig. 4A-B), the effect size was marginal (0.12%). Notably, the observed reduction was even less pronounced than that in the saline-treated control group.

Responses: We appreciate the reviewer's thoughtful comment. Classically, dexmedetomidine (DEX)–induced sedation has been attributed to α2-adrenergic suppression of locus coeruleus (LC) noradrenergic output, leading to disinhibition of preoptic sleep-promoting circuits such as the ventrolateral preoptic nucleus (VLPO). Recent studies, however, demonstrate that intact LC function is not essential for DEX-induced sedative or NREM-like brain states, indicating that other brain regions beyond

the LC can mediate these effects [24-25].

Beyond sedation, the analgesic actions of DEX have been most extensively characterized at the peripheral and spinal levels, where α2-adrenergic receptors on primary afferents and dorsal horn neurons suppress nociceptive transmission. Central analgesic mechanisms are less well defined but emerging, with evidence that DEX can modulate supraspinal pain-related circuits, such as reducing excitatory neuronal activity in the anterior cingulate cortex and attenuating glial activation, thereby alleviating pain-related behaviors [26-27]. Together, these findings suggest that DEX engages multiple brain regions to coordinate analgesia and sleep promotion, which is particularly relevant to chronic pain–sleep comorbidity.

In this context, we observed that intraperitoneal DEX significantly suppressed the SNI-induced elevation of calcium activity in LSD^{GABA} neurons (Fig. 4A–B). Although the peak amplitude change was modest, area-under-the-curve (AUC) analysis revealed a significant reduction following DEX administration ($P = 0.0023$). To further strengthen this observation and address reviewer concerns regarding appropriate controls, we conducted an independent replication experiment including a saline-treated control group. Using a 3000-s analysis window ($n = 7$ mice), DEX again robustly reduced calcium-signal AUC ($P = 0.0024$), whereas saline did not produce a significant effect ($P = 0.0749$), confirming that DEX reproducibly attenuates SNI-evoked LSD^{GABA} activity. These results have been incorporated into the revised manuscript and are presented in Fig. 4A-B.

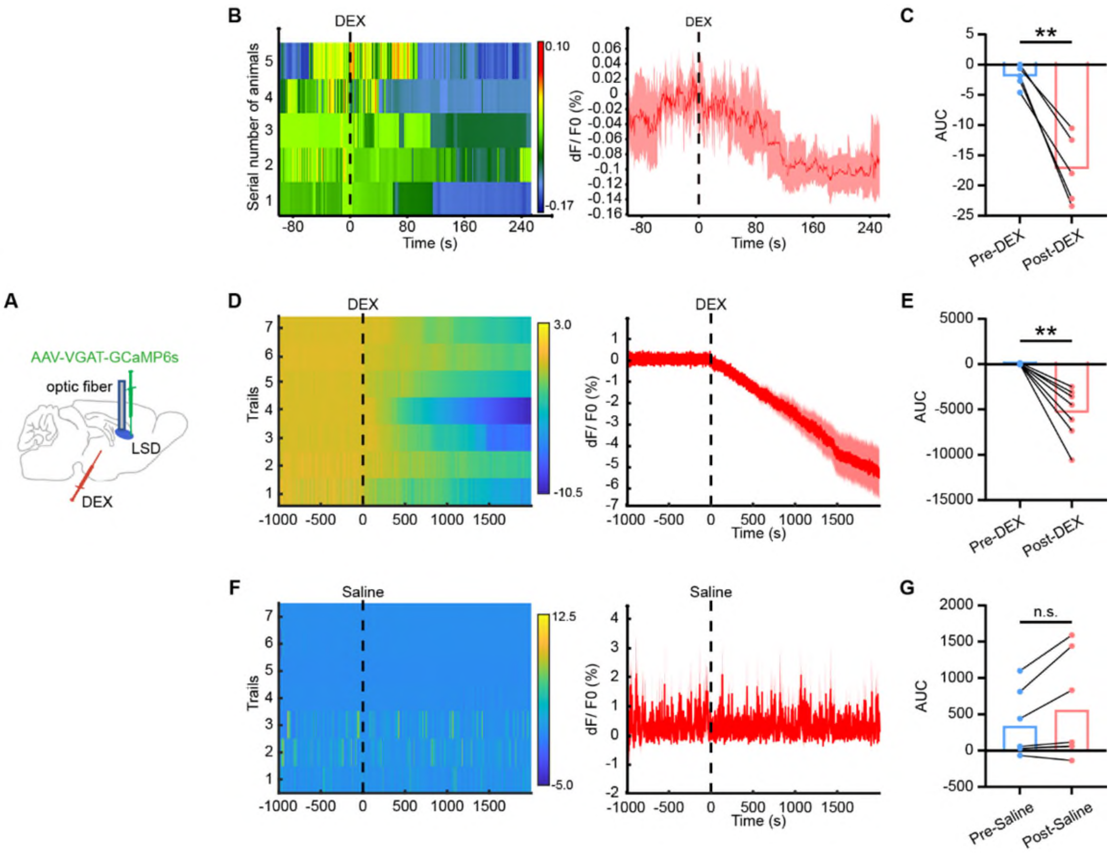

Comment Fig. 13 DEX suppressed the SNI-induced elevation of calcium activity in LSD^{GABA} neurons

Comment 7: The calcium signaling findings are not compelling, as exemplified by Figure 6N. The reported change of 0.004% (calculated per methods as $(F - F_0)/F_0$) lacks both a control comparison and statistical testing, rendering its biological interpretation unreliable.

Responses: We thank the reviewer for this important comment. We agree that the original presentation of Fig. 6N, which highlighted a small mean $\Delta F/F_0$ change, did not adequately demonstrate statistical robustness.

To address this, we re-analyzed the original dataset using an area-under-the-curve (AUC) metric, comparing light-off (−5 to 0 s) and light-on (0 to 5 s) epochs within the same animals, which suggested a suppressive effect during light stimulation. Given the reviewer's concern and the limited power of the initial cohort, we then performed an independent replication with an expanded sample size to increase statistical power and

reduce inter-animal variability, and confirm reproducibility across independent recordings.

In the replication experiment (n = 9 mice), AUC was again significantly reduced during the light-on period relative to light-off (paired t-test, two-tailed: $t(8) = 4.859$, $P = 0.0013$), demonstrating a robust and reproducible suppression of calcium activity by light stimulation. These updated results are now presented in Fig. 6P–R in the revised manuscript.

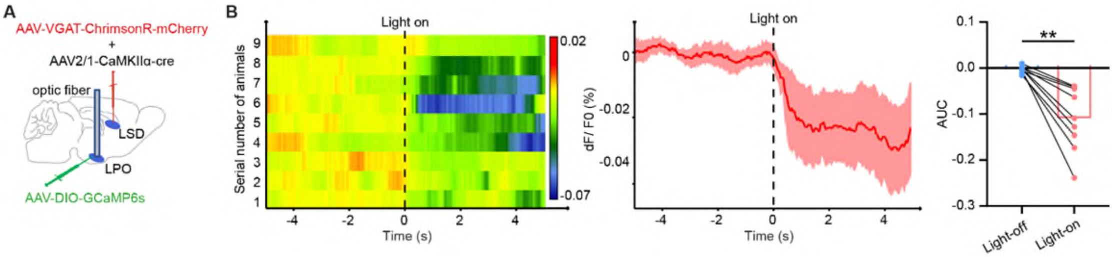

Comment Fig. 14 Optogenetic activation of LSD^{GABA} terminals suppresses Ca^{2+} activity in LPO glutamatergic neurons

Comment 8: A critical control is missing across all chemogenetics experiments: a DIO-mCherry group. This group is essential to account for potential non-specific effects of the viral injection and transgene expression, thereby ensuring that the observed effects are specifically due to the activation of the designated receptor (hM3Dq/hM4Di).

Responses: We thank the reviewer for this important suggestion. To exclude potential off-target effects of CNO and confirm specificity, we added an mCherry control cohort receiving CNO (with the corresponding saline control) and analyzed Wake, NREM, REM and PWT using two-way ANOVA (virus × treatment) followed by Sidak's multiple-comparisons test. In mCherry controls, CNO did not affect any readout (mCherry saline vs CNO: M4 Wake p=0.9631, NREM p=0.9330, REM p=0.9605, PWT p=0.9998; M3 Wake p=0.8998, NREM p=0.8945, REM p=0.7889, PWT p=0.9722). By contrast, under CNO conditions, DREADD-expressing mice differed significantly from mCherry controls for Wake/NREM and PWT (M4: Wake p=0.0002, NREM p=0.0002, PWT p<0.0001; M3: Wake p=0.0099, NREM p=0.0035, PWT p<0.0001), supporting that the observed phenotypes are DREADD-dependent rather than

attributable to CNO alone. The updated Fig. 3 incorporating these control data has been included in the revised manuscript (Fig. 3D, F–H; Supplementary Fig. 4L–P).

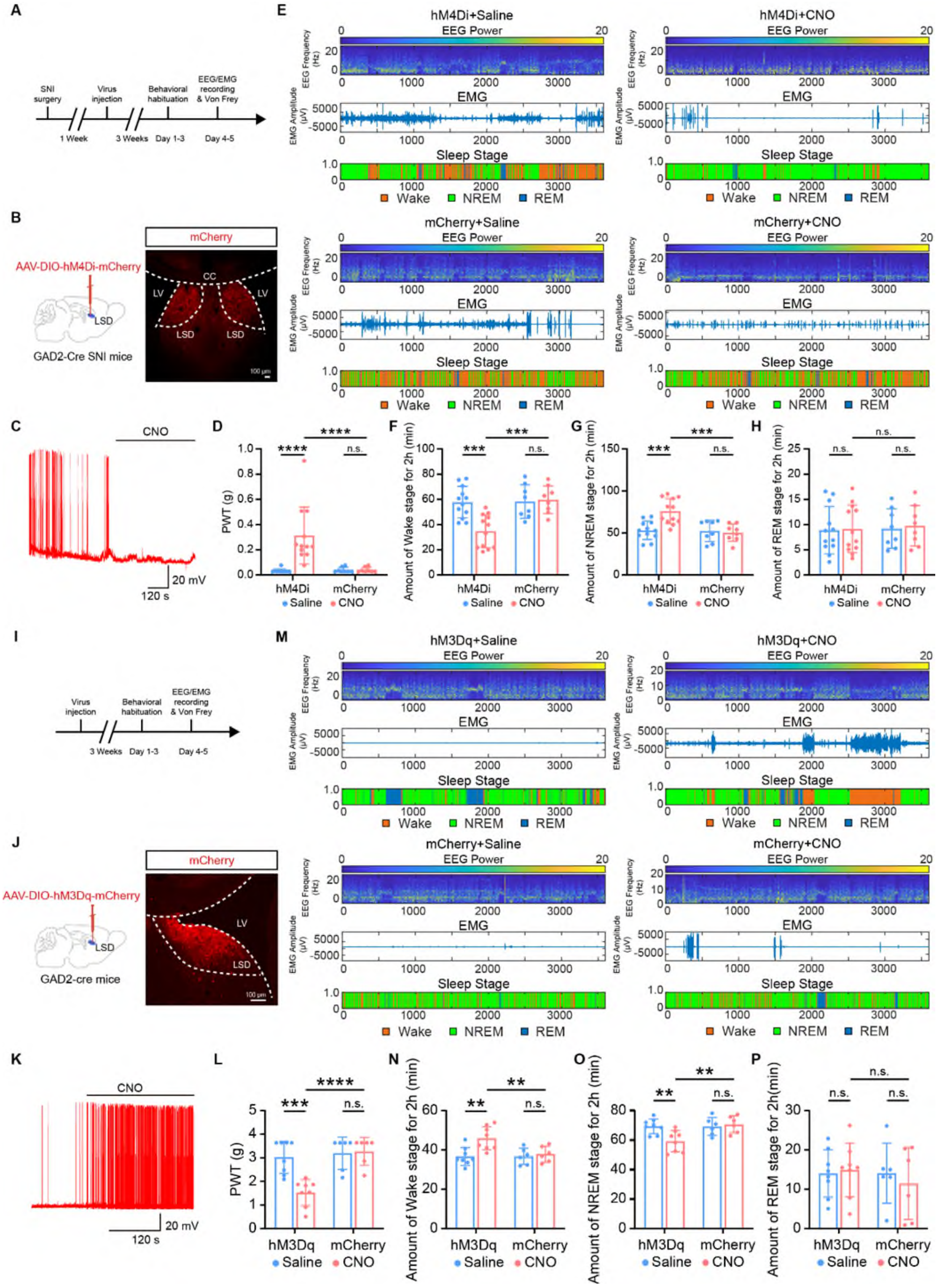

Comment Fig. 4 Chemogenetic manipulation of LSDGABA neurons modulates the comorbidity of chronic pain and sleep disorders

Minor comments:

Comment 1: All color bars in the manuscript should be accompanied by their corresponding numerical values to allow for accurate data interpretation.

Responses: We thank the reviewer for this suggestion. All color bars in the revised manuscript have been updated to include their corresponding numerical values, allowing for accurate interpretation of the data.

Comment 2: The use of arbitrary and inconsistent data ranges undermines the comparability of the results. A prominent example is in Figure 3 O, where the EMG activity for the two groups is displayed on vastly different scales (±200 vs. ±5000), Figure 4F (±200 vs. ±2000), making a direct visual comparison invalid.

Responses: We thank the reviewer for pointing out this important issue. We agree that the use of inconsistent data ranges can compromise visual comparability across groups. In the revised manuscript, we have re-plotted the EMG traces in Fig. 3Q and Fig. 4F using identical y-axis scales for all groups, enabling direct and unbiased visual comparison. The quantitative analyses were not affected by this change and remain unchanged. These revisions improve the clarity and consistency of data presentation (the original Fig. 3O is now presented as Fig. 3E).

Reviewer 3

Chronic pain and sleep disorders frequently co-occur and exacerbate each other, yet the neural circuit mechanisms underlying this comorbidity remain poorly understood. This work reports that hyperactivity of GABAergic neurons in the LSD drives both chronic pain and sleep disorders through projections to glutamatergic neurons and GABAergic neurons in the LPO respectively. Moreover, systemic or local application of DEX alleviated comorbid pain and sleep disorders through blunting the LSD GABAergic neuronal hyperactivity in an α2A-receptor dependent manner. These findings reveal a shared neural substrate for chronic pain and sleep disorders and establish DEX as a potential treatment for this comorbidity through tuning the LSD-driven circuit activity.

Comment 1. Fig. 1I. It is puzzling that DEX has no effect on the REM phase, although the duration of REM stage was also significantly decreased by the SNI (Fig. 1E).

Responses: We thank the reviewer for raising this important point. We agree that it may initially appear puzzling that dexmedetomidine (DEX) does not significantly affect the REM phase, despite the fact that REM duration is reduced in the SNI model.

From a pharmacological perspective, this finding is consistent with the known mechanism of action of DEX. DEX is a highly selective α2-adrenergic receptor agonist that induces a sleep-like state resembling non–rapid eye movement (NREM) sleep. Numerous studies have shown that DEX predominantly promotes NREM sleep and reduces wakefulness, while exerting minimal or inconsistent effects on REM sleep architecture[28-30].

In line with this pharmacological profile, our results indicate that DEX does not significantly restore REM sleep duration in SNI mice. Instead, DEX markedly reduces excessive wakefulness and enhances NREM sleep, thereby improving overall sleep continuity and structure in the SNI model. Thus, although SNI leads to impairments across multiple sleep stages, the therapeutic effects of DEX are mainly mediated through normalization of the Wake–NREM balance rather than direct modulation of REM sleep.

Comment 2. I am wondering why the authors chose to record the EEG/EMG for 6 h (Fig. 1F). What is the rationale for selecting 6 h instead of 12 h or 24 h? I think it better to analyze the time spent in wake, NREM and REM over a 12 h period.

Responses: We thank the reviewer for this thoughtful suggestion regarding the duration of EEG/EMG recordings. The choice of a 6 h recording period was based on pharmacological evidence and the experimental focus of the present study. Previous studies have shown that the sleep-modulating effects of dexmedetomidine (DEX) are most prominent within the first several hours following administration. For example, neural and behavioral effects of DEX have been reported to peak approximately 2 h after dosing[31]. In addition, an oral DEX study showed that dexmedetomidine increased NREM sleep and decreased REM sleep for 6 h (light cycle), and sleep architecture was

therefore analyzed within a 6 h window [32].

Pharmacokinetically, dexmedetomidine exhibits a rapid distribution phase and a relatively short elimination half-life. In humans, the distribution half-life is on the order of ~6–9 min, and the terminal elimination half-life is approximately ~2–3 h. In rodents, the elimination half-life is generally shorter; for example, intravenous dexmedetomidine in rats has been reported to have an elimination half-life of approximately ~1 h. These kinetics support focusing EEG/EMG analyses on an early post-dosing window, such as the 6 h period used here, to capture the dominant pharmacodynamic effects while avoiding additional circadian and recording-stability confounds that arise in 12–24 h recordings.

Based on this established literature, we selected a 6 h EEG/EMG recording window to encompass the period during which DEX exerts its strongest effects on sleep–wake regulation, while providing sufficient temporal coverage to reliably quantify changes in wake, NREM, and REM sleep. This approach also helps minimize confounding influences related to prolonged recording durations, such as variability in behavioral state and recording stability. Within this recording window, we observed consistent and stage-specific changes in wakefulness and NREM sleep in SNI mice, which form the central findings of the study. Although analysis over longer periods (e.g., 12 h or 24 h) may offer additional information regarding longer-term or circadian aspects of sleep regulation, such analyses are beyond the scope of the present work. Future studies examining longer recording periods may further elucidate the long-term and circadian aspects of sleep regulation under chronic pain conditions.

Comment 3. Fig. 3H and Fig. S3A. Please provide the electrophysiological validation of the efficacy of neuronal inhibition by NpHR vs. activation by ChR2. Please revise the Fig. 3 and Fig. 4 legends. Several mistakes occurred.

Responses: We thank the reviewer for this important suggestion. We have now provided electrophysiological validation of the optogenetic efficacy, confirming robust neuronal inhibition by NpHR and reliable neuronal activation by ChR2. These validation data have been added to the revised manuscript (corresponding to Fig. 3K

and Fig. S4B). In addition, we carefully checked and corrected the figure legends and have revised the legends for Fig. 3 and Fig. 4 to address the errors and improve clarity.

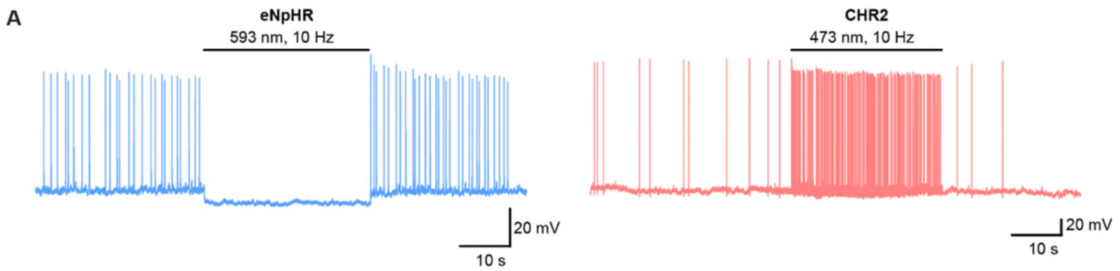

Comment Fig. 15 Electrophysiological validation confirms NpHR inhibition and ChR2 activation

Comment 4. Please draw a schematic timeline for behavioral experiments shown in Fig. 3, Fig. 6, Fig. 7 and Fig. 8.

Responses: We thank the reviewer for this suggestion. A schematic timeline summarizing the behavioral experiments shown in Fig. 3, Fig. 6, Fig. 7, and Fig. 8 has now been created and included in the revised manuscript to clearly illustrate the experimental procedures and timing.

Comment 5. Fig. S2A, the whole-brain c-Fos mapping data should be quantified to illustrate the relative differences of c-Fos+ cell numbers among each brain region. Similarly, please add the quantitative analysis of the whole-brain output tracing data shown in Fig. S4A.

Responses: We thank the reviewer for this constructive suggestion. We have now quantified the whole-brain c-Fos mapping data to illustrate the relative differences in c-Fos–positive cell numbers across brain regions (Comment Fig. 16 A). These quantitative results have been added to the revised manuscript as Supplementary Fig. 3B.

Similarly, we have performed quantitative analysis of the whole-brain output tracing data shown in Fig. S4A (Comment Fig. 16 B), and the corresponding results have been included in Supplementary Fig. 5R of the revised manuscript.

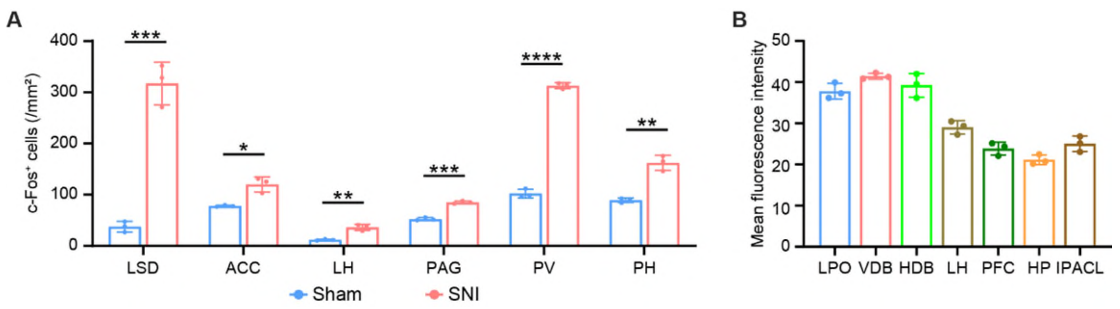

Comment Fig. 16 Quantitative mapping of whole-brain c-Fos and LSD^{GABA} outputs

Comment 6. Fig. 4A-B. It is necessary to further test whether DEX could alleviate the rise in Ca2+ signals when the SNI-treated animals received mechanical stimulus or experienced sleep-wake transitions (as shown in Fig. 2G and H).

Responses: We thank the reviewer for this constructive suggestion to further examine whether dexmedetomidine (DEX) could attenuate the rise in Ca^{2+} signals in SNI-treated animals during mechanical stimulation or across sleep–wake transitions, as shown in Fig. 2G–H. In response, we performed additional experiments addressing both conditions. For the mechanical-stimulus condition, Ca^{2+} responses in the saline-treated SNI group showed a robust increase after stimulation (paired t test, two-tailed: t = 5.472, df = 7, P = 0.0009; n = 8). In contrast, in SNI mice receiving DEX, the stimulus-evoked increase was markedly blunted and did not reach statistical significance (paired t test, two-tailed: t = 2.272, df = 6, P = 0.0635; n = 7), indicating that DEX attenuates the stimulation-associated elevation of Ca^{2+} activity (Comment Fig. 17A-B).

For sleep–wake transitions, saline-treated SNI mice exhibited a clear state-dependent difference in Ca^{2+} activity between wake and NREM (paired t test, two-tailed: t = 9.029, df = 5, P = 0.0003; n = 6). Notably, this wake–NREM difference was abolished after DEX treatment (paired t test, two-tailed: t = 0.07036, df = 5, P = 0.9466; n = 6), supporting that DEX dampens sleep–wake transition–associated Ca^{2+} fluctuations in the SNI comorbidity model (Comment Fig. 17C-D).

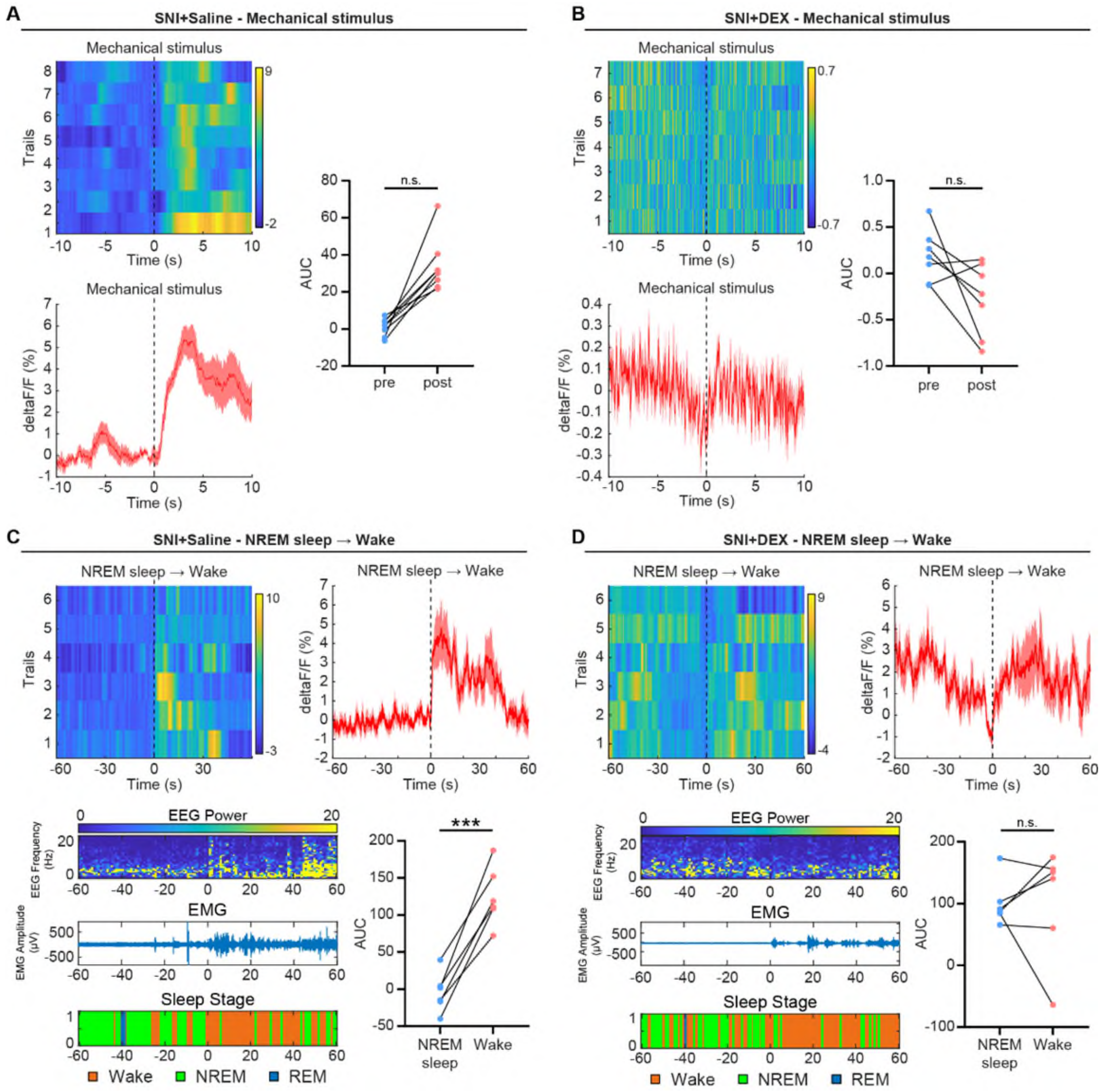

Comment Fig. 17 DEX attenuates mechanical stimulus– and sleep–wake transition–associated Ca^{2+} elevations in SNI mice

Comment 7. Fig. 4K should provide a quantification of the relative expression abundance of α2A in LSD GABAergic neurons. The authors should also verify the knockdown efficiency of AAV-Vgat-α2A-shRNA in Fig. 4Q.

Responses: We thank the reviewer for this request. We have now quantified the relative abundance of α2A expression in LSD GABAergic neurons, showing that 96.4% of LSD GABA ($VGAT^{+}$) neurons were α2A-positive (Fig. 4K; Comment Fig. 18-b). To validate the efficiency of the AAV-VGAT-α2A-shRNA knockdown, we quantified α2A adrenergic receptor (Adra2a) mRNA in LSD tissue from SNI mice injected with AAV-VGAT-α2A-shRNA or AAV-VGAT-α2A-scramble. This approach is consistent with

prior in vivo AAV-shRNA studies that validate knockdown by RT-qPCR from AAV-infected brain tissue punches[33]. Our results showed a significant reduction of Adra2a transcript levels in the shRNA group compared with scramble controls, confirming effective knockdown (Comment Fig. 18C).

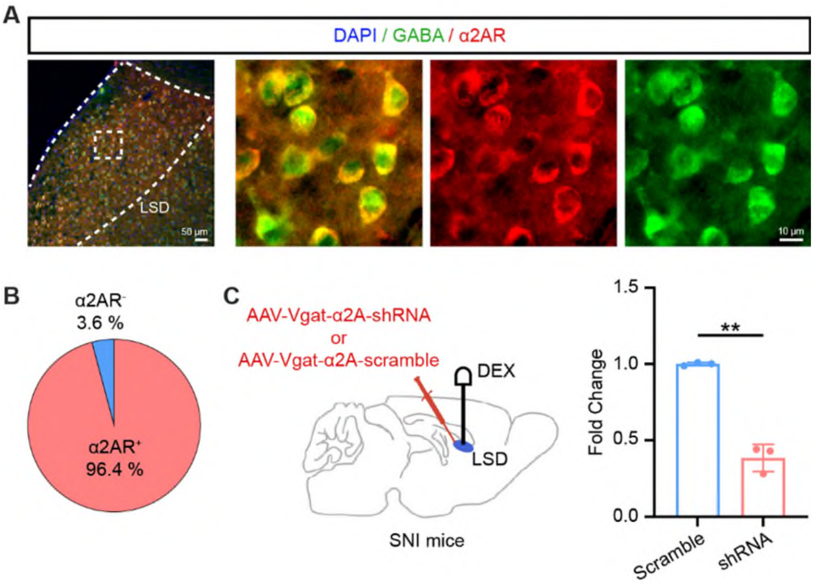

Comment Fig. 18 Quantification of α2A enrichment in LSD^{GABA} neurons and validation of AAV-shRNA knockdown efficiency

Comment 8. Fig. 6 and Fig. 7. It is highly recommended that the authors performed more whole-cell patch-clamp recordings to evaluate any changes in synaptic plasticity (PPR, I-O curve of light-evoked IPSC amplitude) of the LSD→LPOGlu and LSD→LPOGABA projection in the comorbidity mice.

Responses: We thank the reviewer for this valuable suggestion. In response, we have performed additional whole-cell patch-clamp recordings to further evaluate synaptic plasticity of both the LSD^{GABA}→LPO^{Glu} and LSD^{GABA}→LPO^{GABA} projections in the comorbidity model.

Specifically, we quantified the paired-pulse ratio (PPR) and constructed input–output (I–O) curves of light-evoked IPSC amplitudes to assess potential changes in presynaptic release probability and synaptic strength. The results demonstrate significant alterations in synaptic transmission in SNI mice compared with sham controls, as reflected by changes in PPR and reduced I–O relationships.

These new electrophysiological data have been incorporated into the revised manuscript and are now presented in Supplementary Fig. 7, providing a more comprehensive characterization of synaptic plasticity within the LSD→LPO circuit.

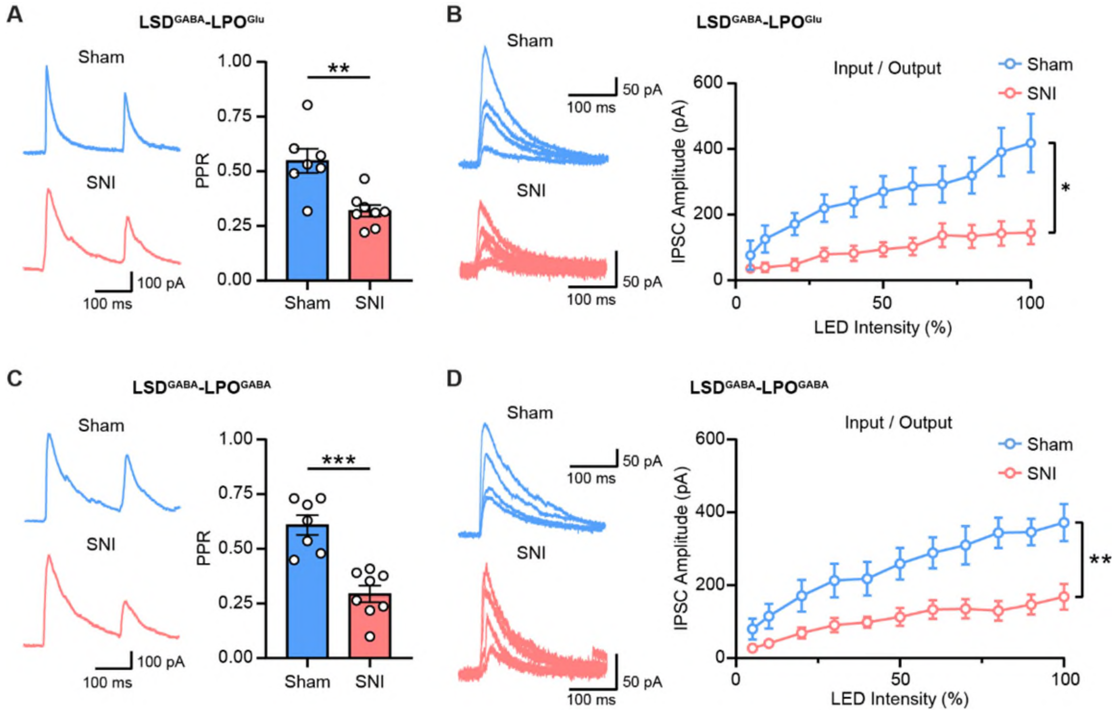

Comment Fig. 19 Electrophysiological analyses reveal synaptic plasticity changes in LSD→LPO projections in comorbidity mice

Comment 9. Fig. 8B and C. The legend said “The c-Fos expression of LSD and LPO after intraperitoneal injection of dexmedetomidine in SNI mice”, but no any c-Fos data in the LSD region was shown. In addition, compared with the SNI only group, the SNI+DEX group showed an elevated c-Fos expression, indicating the possibility that DEX blocked the LSD GABAergic neurons through α2A receptor, resulting in a disinhibition of the downstream LPO neurons. However, Fig. 8A shows the expression of α2A receptor in the LPO neurons. Then how to exclude the possibility that DEX acts directly on the α2A in LPO neurons to inhibit the LPO neuronal activity instead of indirect disinhibition?

Responses: We thank the reviewer for this important comment. First, we agree that the original legend of Fig. 8B–C was inaccurate. Although the legend stated “c-Fos

expression of LSD and LPO after intraperitoneal injection of dexmedetomidine (DEX) in SNI mice", Fig. 8B–C only presents c-Fos signals quantified in the LPO. The corresponding LSD c-Fos data are shown in Fig. 2A and Fig. 4D. We have therefore corrected the legend and related text to clearly indicate that Fig. 8B–C displays LPO c-Fos across four groups (Sham, SNI, SNI+Saline, and SNI+DEX).

Second, the reviewer proposed that DEX might inhibit LSD^{GABA} neurons via α2A receptors and thereby disinhibit downstream LPO neurons, and asked how we can exclude a direct action of DEX on α2A receptors expressed in LPO neurons. Indeed, both prior studies and our data indicate that α2A adrenergic receptors are expressed in LPO neurons (Fig. 8A). α2A receptors are generally known to couple to inhibitory Gi/o proteins and thereby inhibit adenylate cyclase (AC) activity and downstream cAMP signaling[34-35].

However, Specifically, systemic DEX (i.p.) counteracted the SNI-associated decrease in LPO c-Fos (Fig. 8B–C), indicating restoration/enhancement of LPO activity. Moreover, whole-cell patch-clamp recordings in GAD2-Cre and CaMKIIα-Cre mice (AAV-DIO-EGFP in LPO) revealed that significantly increased firing rates in both LPO^{GABA} and LPO^{Glu} neurons (Fig. 8D–E, J-K), which is inconsistent with a dominant direct suppression of LPO neuronal activity.

In addition, prior work reported that selective disruption of α2A receptors in the LPO does not alter DEX-induced sedation[36], arguing against LPO α2A receptors being necessary for canonical DEX effects. To further strengthen this point in our study, we performed an in which DEX was locally infused into the LPO via cannulation (2 μM) and sleep–wake states were quantified over a 2-h recording period together with mechanical sensitivity assessment. Local LPO DEX produced no significant effects on PWT (unpaired two-tailed t test, t(8)=0.2079, P=0.8405), wake duration (t(8)=0.1020, P=0.9213), NREM sleep (t(8)=0.1090, P=0.9159), or REM sleep (t(8)=0.4733, P=0.6487) compared with saline controls (Comment Fig. 20). Together, these results argue against a direct action of DEX on LPO α2A receptors being sufficient to account for the observed analgesic and sleep-promoting effects, and instead support an indirect mechanism whereby DEX regulates LPO activity predominantly via upstream circuitry

(including the LSD→LPO pathways).

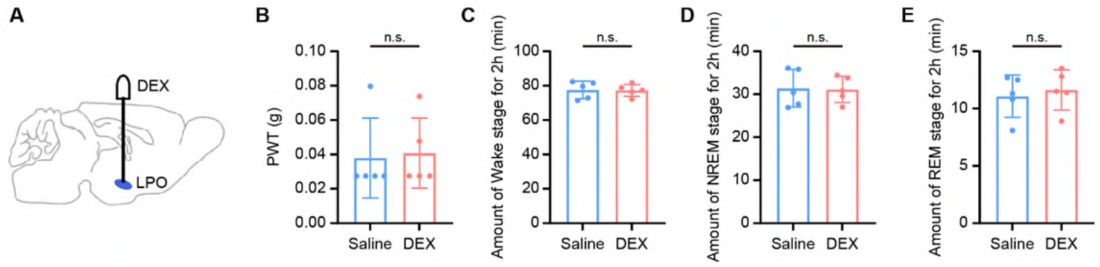

Comment Fig. 20 Intra-LPO dexmedetomidine administration does not alter sleep architecture or pain behaviors.

Comment 10. The experimental design of Fig. 8F-M seems wrong. Since the LPO GABA or Glu neurons innervated by LSD have already been strongly inhibited by chemogenetic manipulations, it is reasonably deduced that intra-LSD injection of DEX could not further activate the LPO neurons to reverse CNO-induced mechanical allodynia and sleep deficits.

In contrast, to demonstrate that DEX alleviates pain hypersensitivity and comorbid sleep disorder via the LSD→LPO circuit, the authors should activate the LPOGlu-projecting LSD neurons or activate the LPOGABA-projecting LSD neurons, then check whether DEX still attenuates mechanical allodynia and sleep deficits. Or else, if DEX indeed exerts the beneficial effects through activating the LPO neurons (Fig. 8E and K), then optogenetic or chemogenetic inhibition of the LPO glu or GABA neurons should also block the analgesic or sleep-improving effect of DEX.

Responses: We appreciate the reviewer’s careful evaluation of the experimental logic in Fig. 8F–M. In the revised manuscript, the original Fig. 8F–M has been renumbered as Fig. 8F–I and Fig. 8L–Q. We would like to clarify that the experiments in Fig. 8F–M were designed to test the necessity of downstream LPO neuronal activity for the behavioral effects of intra-LSD dexmedetomidine (DEX), rather than to assess whether DEX could further activate LPO neurons following chemogenetic inhibition. Importantly, hM4Di/CNO-mediated inhibition is generally understood to reduce neuronal excitability and synaptic output, rather than to completely silence neuronal activity. Under this framework, inhibition of LPO neurons is expected to attenuate, but

not necessarily abolish, the effects of upstream modulation. Our results show that, in naive mice, chemogenetic inhibition of LPO GABAergic or glutamatergic neurons was first used to induce mechanical hypersensitivity and/or sleep disruption, and intra-LSD DEX was then able to counteract these CNO-evoked deficits. Notably, chemogenetic inhibition significantly weakened the magnitude of DEX-induced analgesia and sleep promotion, while leaving REM sleep largely unaffected, supporting the necessity of LPO neuronal activity for DEX-induced analgesia and sleep promotion. Consistent with our experimental logic, previous work delivered CNO before dexmedetomidine to place the targeted neurons under chemogenetic manipulation and then evaluated dexmedetomidine-evoked outcomes resulting from activation of the targeted brain region[37].

Following the reviewer's suggestion, we performed additional experiments to directly test whether suppressing downstream LPO activity diminishes the efficacy of intra-LSD DEX under pathological conditions. Specifically, in the SNI comorbidity model, we first administered intra-LSD DEX to establish its analgesic and sleep-promoting effects, and then delivered CNO to chemogenetically inhibit downstream LPO GABAergic or glutamatergic neurons, thereby assessing whether inhibition could attenuate the already engaged DEX effects. Two-way mixed-design (repeated-measures) ANOVA with Treatment (LSD-saline vs LSD-DEX) and CNO (saline vs CNO, i.p.) revealed significant Treatment × CNO interactions for mechanical sensitivity (PWT: $F(1,10)=10.29$, $P=0.0094$, Comment Fig. 21B), wake duration ($F(1,10)=14.88$, $P=0.0032$, Comment Fig. 21C), and NREM sleep ($F(1,10)=6.48$, $P=0.0291$, Comment Fig. 21D), demonstrating that CNO-mediated inhibition significantly attenuated the analgesic and sleep-promoting effects of DEX. Interpreting these outcomes in a pathway-specific manner, the attenuation of the DEX-induced increase in PWT supports a key role for the LSD^{GABA}→LPO^{Glu} pathway in mediating analgesia, whereas the attenuation of DEX-induced wake suppression and NREM promotion supports a predominant contribution of the LSD^{GABA}→LPO^{GABA} pathway to sleep–wake regulation. Notably, CNO alone had no effect in saline-treated animals, whereas it reduced the magnitude of DEX-induced changes in these measures. In contrast, REM

sleep was not affected (Treatment × CNO interaction: P=0.6946, Comment Fig. 21E). Together, these results provide convergent evidence that intact downstream LPO neuronal activity is required for the full expression of intra-LSD DEX benefits, supporting the involvement of LSD→LPO circuitry in coordinating pain relief and sleep regulation.

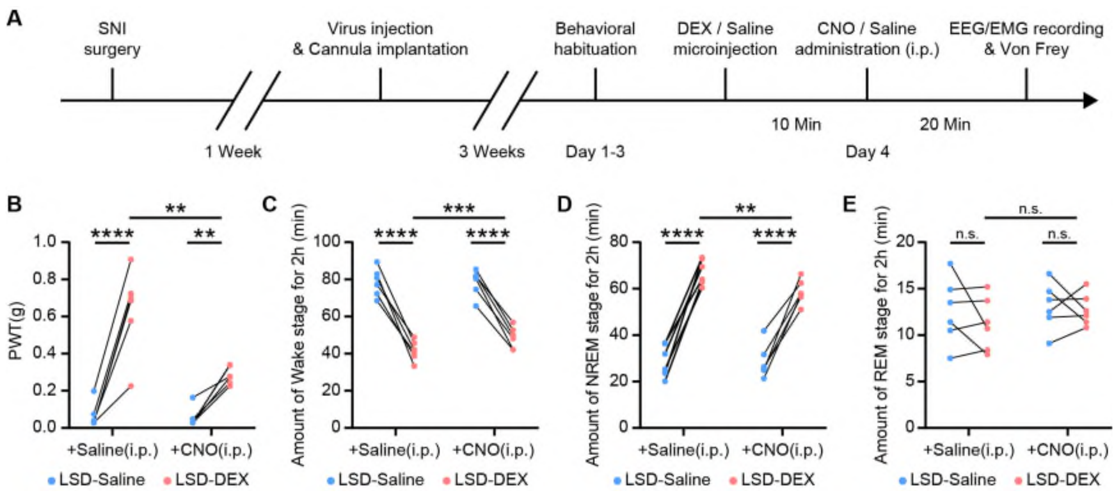

Comment Fig. 21 Downstream LPO inhibition blunts intra-LSD DEX–induced analgesic and sleep-promoting effects in SNI mice.

Reference

[1]Roman Yasenkov, Tom Deboer. Circadian regulation of sleep and the sleep EEG under constant sleep pressure in the rat. Sleep，2010，33（5）：631-641

[2]Rick van Dorp, Elisa Rolleri, Tom Deboer. Sleep and the sleep electroencephalogram in C57BL/6 and C3H/HeN mice. Journal of sleep research，2024，33（2）：e14062

[3]Fengfei Ding, John O'Donnell, Qiwu Xuet al. Changes in the composition of brain interstitial ions control the sleep-wake cycle. Science (New York, N.Y.)，2016，352（6285）：550-555

[4]Shohei Nishimon, Noriaki Sakai, Seiji Nishino. Sake yeast induces the sleep-promoting effects under the stress-induced acute insomnia in mice. Scientific reports，2021，11（1）：20816

[5]Haiyan Sun, Zhilin Li, Zhentong Qiuet al. A common neuronal ensemble in nucleus accumbens regulates pain-like behaviour and sleep. Nature communications，2023，14（1）：4700

[6]Ya-Dong Li, Yan-Jia Luo, Wei-Kun Suet al. Anterior cingulate cortex projections to the dorsal medial striatum underlie insomnia associated with chronic pain. Neuron，2024，112（8）：1328-1341

[7]Hue Jung Park, Young Hoon Kim, Hyun Jung Kohet al. Analgesic effects of dexmedetomidine in vincristine-evoked painful neuropathic rats. Journal of Korean medical science，2012，27（11）：1411-1417

[8]Hui Chen, Xiang Xu, Xiao-Yu Yanget al. Systemic dexmedetomidine attenuates mechanical allodynia through extracellular sign db type 2 diabetic mice. Neuroscience letters，2017，657：126-133

[9]Jessie Muir, Zachary S. Lorsch, Charu Ramakrishnanet al. In Vivo Fiber Photometry Reveals Signature of Future Stress Susceptibility in Nucleus Accumbens. Neuropsychopharmacology : official publication of the American College of Neuropsychopharmacology，2018，43（2）：255-263

[10]Ya-Dong Li, Yan-Jia Luo, Wei-Kun Suet al. Anterior cingulate cortex projections to the dorsal medial striatum underlie insomnia associated with chronic pain. Neuron，2024，112（8）：1328-1341

[11]Claire Gao, Yan Leng, Jun Maet al. Two genetically, anatomically and functionally distinct cell types segregate across anteroposterior axis of paraventricular thalamus. Nature neuroscience，2020，23（2）：217-228

[12]Sasa Teng, Fenghua Zhen, Li Wanget al. Control of non-REM sleep by ventrolateral medulla glutamatergic neurons projecting to the preoptic area. Nature communications，2022，13（1）：4748

[13]Eyal Vardy, J. Elliott Robinson, Chia Liet al. A New DREADD Facilitates the Multiplexed Chemogenetic Interrogation of Behavior. Neuron，2015，86（4）：936-946

[14]Natale R. Sciolino, Nicholas W. Plummer, Yu-Wei Chenet al. Recombinase-Dependent Mouse Lines for Chemogenetic Activation of Genetically Defined Cell?Types. Cell reports，2016，15（11）：2563-2573

[15]Stephen V. Mahler, Elena M. Vazey, Jacob T. Beckleyet al. Designer receptors show role for ventral pallidum input to ventral tegmental area in cocaine seeking. Nature neuroscience，2014，17（4）：577-585

[16]Hai-Ying Liang, Zhi-Jin Chen, Hui Xiaoet al. nNOS-expressing neurons in the vmPFC transform

pPVT-derived chronic pain signals into anxiety behaviors. Nature communications，2020，11（1）：2501

[17]Shinjae Chung, Franz Weber, Peng Zhonget al. Identification of preoptic sleep neurons using retrograde labelling and gene profiling. Nature，2017，545（7655）：477-481

[18]Jennifer Smith, Adam Honig-Frand, Hanna Antilaet al. Regulation of stress-induced sleep fragmentation by preoptic glutamatergic neurons. Current biology : CB，2024，34（1）：12-23

[19]Matthew B. Pomrenze, Simone M. Giovanetti, Rajani Maiyaet al. Dissecting the Roles of GABA and Neuropeptides from Rat Central Amygdala CRF Neurons in Anxiety and Fear Learning. Cell reports，2019，29（1）：13-21

[20]Ed S. Lein, Michael J. Hawrylycz, Nancy Aoet al. Genome-wide atlas of gene expression in the adult mouse brain. Nature，2007，445（7124）：168-176

[21]Edward H. Nieh, Gillian A. Matthews, Stephen A. Allsopet al. Decoding neural circuits that control compulsive sucrose seeking. Cell，2015，160（3）：528-541

[22]Jessie Muir, Zachary S. Lorsch, Charu Ramakrishnanet al. In Vivo Fiber Photometry Reveals Signature of Future Stress Susceptibility in Nucleus Accumbens. Neuropsychopharmacology : official publication of the American College of Neuropsychopharmacology，2018，43（2）：255-263

[23]Jie Deng, Li Chen, Cui-Cui Liuet al. Distinct Thalamo-Subcortical Circuits Underlie Painful Behavior and Depression-Like Behavior Following Nerve Injury. Advanced science (Weinheim, Baden-Wurttemberg, Germany)，2024，11（34）：e2401855

[24]Berta Anuncibay Soto, Ying Ma, Mathieu Nolletet al. The locus coeruleus maintains core body temperature and protects against hypothermia during dexmedetomidine-induced sedation. Proceedings of the National Academy of Sciences of the United States of America，2025，122（41）：e1872089174

[25]Zhe Zhang, Valentina Ferretti, Lke Güntanet al. Neuronal ensembles sufficient for recovery sleep and the sedative actions of α2 adrenergic agonists. Nature neuroscience，2015，18（4）：553-561

[26]Ling Liu, Zhihao Luo, Yuanying Maiet al. Dexmedetomidine relieves inflammatory pain by enhancing GABAergic synaptic activity in pyramidal neurons of the anterior cingulate cortex. Neuropharmacology，2023，240：109710

[27]Yaohua Chen, Yuping Li, Chunxiang Liet al. Dexmedetomidine alleviates pain in MPTP-treated mice by activating the AMPK/mTOR/NF-κB pathways in astrocytes. Neuroscience letters，2022，791：136933

[28]Berta Anuncibay Soto, Ying Ma, Mathieu Nolletet al. The locus coeruleus maintains core body temperature and protects against hypothermia during dexmedetomidine-induced sedation. Proceedings of the National Academy of Sciences of the United States of America，2025，122（41）：e1872089174

[29]Ying Zhang, Jiaxin Li, Yan Liet al. Dexmedetomidine Promotes NREM Sleep by Depressing Oxytocin Neurons in the Paraventricular Nucleus in Mice. Neurochemical research，2024，49（10）：2926-2939

[30]Huizi Liu, Hanwei Wei, Shaojie Qianet al. Effects of dexmedetomidine on postoperative sleep quality: a systematic review and meta-analysis of randomized controlled trials. BMC anesthesiology，2023，23（1）：88

[31]Laura E. Nelson, Jun Lu, Tianzhi Guoet al. The alpha2-adrenoceptor agonist dexmedetomidine converges on an endogenous sleep-promoting pathway to exert its sedative effects. Anesthesiology，2003，98（2）：428-436

[32]Zhen-Xin Feng, Hui Dong, Wei-Min Quet al. Oral Delivered Dexmedetomidine Promotes and Consolidates Non-rapid Eye Movement Sleep via Sleep-Wake Regulation Systems in Mice. Frontiers in pharmacology，2018，9：1196

[33]M. B. Pomrenze, S. M. Giovanetti, R. Maiyaet al. Dissecting the Roles of GABA and Neuropeptides from Rat Central Amygdala CRF Neurons in Anxiety and Fear Learning. Cell Rep，2019，29（1）：13-21

[34]Xi-Yao Gu, Ben-Long Liu, Kai-Kai Zanget al. Dexmedetomidine inhibits Tetrodotoxin-resistant Nav1.8 sodium channel activity through Gi/o-dependent pathway in rat dorsal root ganglion neurons. Molecular brain，2015，8：15

[35]Brian P. Ramos, David Stark, Luis Verduzcoet al. Alpha2A-adrenoceptor stimulation improves prefrontal cortical regulation of behavior through inhibition of cAMP signaling in aging animals. Learning & memory (Cold Spring Harbor, N.Y.)，2006，13（6）：770-776

[36]Zhe Zhang, Valentina Ferretti, Lke Güntanet al. Neuronal ensembles sufficient for recovery sleep and the sedative actions of α2 adrenergic agonists. Nature neuroscience，2015，18（4）：553-561

[37]Gaolin Qiu, Ying Wu, Zeyong Yanget al. Dexmedetomidine Activation of Dopamine Neurons in the Ventral Tegmental Area Attenuates the Depth of Sedation in Mice. Anesthesiology，2020，133（2）：377-392

Dear Editor and Reviewers:

Thank you for the opportunity to revise our manuscript entitled "**LSD GABAergic neurons participate in dexmedetomidine-improving the chronic pain/sleep disorders comorbidities by regulating different types of LPO neurons" (NCOMMS-25-62717B)**. We have carefully addressed all comments from the reviewers and revised the manuscript accordingly.

REVIEWER COMMENTS

Reviewer #1 (Remarks to the Author):

Thank you to the authors for thoroughly addressing the reviewers' questions and incorporating the suggested experiments. These revisions have strengthened both the manuscript and the conclusions drawn from the data. I recommend the current manuscript for publication.

Responses: We are grateful to the reviewer for the encouraging comments and for recommending acceptance of our manuscript.

Reviewer #2 (Remarks to the Author):

The authors have done a remarkable job to address all of my concerns about the original manuscript. The manuscript reports an impressive set of well conducted experiments supporting their claim.

Responses: We thank the reviewer for the positive comments and recognition of our revisions.

Reviewer #3 (Remarks to the Author):

The resubmission from Fan et al. includes numerous additional experiments which significantly strengthen the manuscript and satisfy the reviewer's initial concerns. Some additional comments are required to be addressed before considering publication of the manuscript.

(1) Supplementary Fig. 5A. CTB should be changed into "LSDGABA mCherry+ fibers". Panel (R) should be moved to Panel (B). Panel (B) should be changed to (C), and so on.

Responses: We thank the reviewer for this helpful suggestion. In Supplementary Fig. 5A, "CTB" has been changed to "LSD^{GABA} $mCherry^{+}$ fibers". In addition, panel (R) has been moved to panel (B), and the subsequent panels have been relabeled accordingly.

(2) I highly recommend integrating Comment Fig. 7 (confirmation of the knock down efficacy) into the manuscript.

Responses: We appreciate the reviewer's valuable suggestion. We agree that confirmation of the knockdown efficacy is important. Accordingly, the data originally shown in Comment Fig. 7 have now been incorporated into the revised manuscript and are presented in Fig. 5N.

(3) Similarly, Comment Fig. 17 should also be included into the main manuscript.

Responses: We thank the reviewer for pointing this out. Accordingly, the data originally shown in Comment Fig. 17 have now been incorporated into the main manuscript and are presented in Fig. 4E–H.

(4) I still think that the experimental design of Fig. 8F–I and Fig. 8L–Q is problematic. The authors said that intra-LSD DEX was able to counteract CNO-evoked mechanical hypersensitivity and/or sleep disruption. But how to explain the results? How does DEX

achieve this effect under the condition of LPO neuronal inhibition by CNO? In other words, what are the mechanisms underlying this reversal effect of DEX?

Instead, I would suggest that the authors delete these panels (Fig. 8F–I and Fig. 8L–Q) and replace them with the Comment Fig. 21, which straightforwardly demonstrates that CNO-mediated LPO neuronal inhibition significantly attenuates the analgesic and sleep-promoting effects of DEX. However, Comment Fig. 21 B and C should clearly show the detailed chemogenetic inhibition target (LPO GABA or LPO Glu) for each behavioral result. Moreover, for Comment Fig. 21A, it is better to perform CNO/saline i.p. administration before (not after) the intra-LSD DEX/saline microinjection?

Responses: We sincerely appreciate the reviewer's insightful and rigorous evaluation of this experiment. We fully agree that the original presentation of Fig. 8F–I and Fig. 8L–Q could lead to confusion in terms of experimental logic and interpretation. Following the reviewer's suggestion, we have removed these panels from the revised manuscript and replaced them with the new data originally shown as Comment Fig. 21, which were obtained in SNI comorbidity mice and more directly demonstrate that chemogenetic inhibition of downstream LPO neurons significantly attenuates the analgesic and sleep-promoting effects of intra-LSD DEX. These data are now presented in Fig. 9F–H (for the LSD^{GABA}→LPO^{Glu} circuit) and Fig. 9K–O (for the LSD^{GABA}→LPO^{GABA} circuit).We have also updated the figure to explicitly indicate whether chemogenetic inhibition was performed in the LSD^{GABA}→LPO^{Glu} or LSD^{GABA}→LPO^{GABA} circuit, with the corresponding viral strategy and representative immunofluorescence images now shown in Fig. 9G and Fig. 9L, respectively. For the LSD^{GABA}→LPO^{Glu} circuit, AAV2/1-CaMKIIα-Cre was injected into the LSD, and Cre-dependent AAV-DIO-hM4Di-mCherry was injected into the LPO. For the LSD^{GABA}→LPO^{GABA} circuit, AAV2/1-VGAT-Cre was injected into the LSD, and Cre-dependent AAV-DIO-hM4Di-mCherry was injected into the LPO. In both experiments, a cannula was implanted into the LSD for local dexmedetomidine administration.

We also appreciate the reviewer's important suggestion regarding the sequence of

drug administration. In the revised experiments, we adopted a DEX-first, CNO-later paradigm. This design allowed us to test whether the maintenance of established DEX-induced analgesia and sleep promotion requires ongoing activity of specific LPO neuronal subpopulations. Consistent with our findings that intra-LSD dexmedetomidine administration significantly increases the firing rates of both LPO^{GABA} and LPO^{Glu} neurons (Fig. 9D-E, I-J), we next asked whether this sustained LPO activity is necessary for the persistence of DEX effects. Our new data show that chemogenetic inhibition of either LSD^{GABA}→LPO^{Glu} or LSD^{GABA}→LPO^{GABA} circuits, after DEX had already exerted its effects, significantly attenuated these effects. These findings demonstrate that sustained activity in downstream LPO neurons is required not only for the initiation but also for the full expression and maintenance of DEX-induced behavioral effects.

Regarding the choice of administration sequence, we acknowledge that administering CNO before DEX represents a more classical blockade design. However, our post-treatment paradigm was intentionally designed to address a distinct but equally important question: whether the continuous integrity of the LSD→LPO circuit is necessary for the persistence of DEX's effects. This approach is supported by precedent. Zhou et al. and Smith et al. both employed a DEX-first, CNO-later paradigm in neural circuit studies[1] [2]. Moreover, Iqbal et al. used a similar post-treatment chemogenetic inhibition design to demonstrate that previously established aversive behavior could be suppressed by subsequent circuit-specific modulation[3]. These studies collectively support our approach of administering DEX first, followed by CNO, to assess whether the established effects of DEX require the integrity of the LSD→LPO circuit.
Importantly, our revised data now show that CNO-mediated inhibition significantly attenuates both the analgesic and sleep-promoting effects of DEX, providing strong evidence that the LSD→LPO circuitry is essential for the sustained behavioral effects of DEX in the context of chronic pain-related sleep disruption.

We thank the reviewer again for the constructive feedback, which has substantially improved the clarity and rigor of our manuscript.

Reference

1. Zhang, Y., et al., Dexmedetomidine Promotes NREM Sleep by Depressing Oxytocin Neurons in the Paraventricular Nucleus in Mice. Neurochem Res, 2024. **49**(10): p. 2926-2939.
2. Zhang, Z., et al., Neuronal ensembles sufficient for recovery sleep and the sedative actions of alpha2 adrenergic agonists. Nat Neurosci, 2015. **18**(4): p. 553-561.
3. Iqbal, Z., et al., Adrenergic signalling to astrocytes in anterior cingulate cortex contributes to pain-related aversive memory in rats. Commun Biol, 2023. **6**(1): p. 10.
